# From Tea to Functional Foods: Exploring *Caryopteris mongolica* Bunge for Anti-Rheumatoid Arthritis and Unraveling Its Potential Mechanisms

**DOI:** 10.3390/nu16244311

**Published:** 2024-12-13

**Authors:** Xin Dong, Zhi Wang, Yao Fu, Yuxin Tian, Peifeng Xue, Yuewu Wang, Feiyun Yang, Guojing Li, Ruigang Wang

**Affiliations:** 1 Key Laboratory of Plants Adversity Adaptation and Genetic Improvement in Cold and Arid Regions of Inner Mongolia, Inner Mongolia Agricultural University, Hohhot 010018, China; dongx1128@163.com (X.D.); yangfeiyun@imau.edu.cn (F.Y.); liguojing@imau.edu.cn (G.L.); 2 Department of Pharmacy, Inner Mongolia Medical University, Jinshan Development Zone, Hohhot 010110, China; 15184717757@163.com (Z.W.); fy15147172014@163.com (Y.F.); tyx199856@163.com (Y.T.); xpfdc153@163.com (P.X.); wywimmu@163.com (Y.W.)

**Keywords:** *Caryopteris mongolica* Bunge, rheumatoid arthritis, inflammatory response, mechanism, metabolomics, transcriptomics

## Abstract

Background: *Caryopteris mongolica* Bunge (CM) shows promising potential for managing rheumatoid arthritis (RA) and digestive disorders, attributed to its rich content of bioactive compounds such as polyphenols and flavonoids. Despite its common use in herbal tea, the specific mechanisms underlying CM’s anti-inflammatory and joint-protective effects remain unclear, limiting its development as a functional food. This study investigated the effects of aqueous CM extract on RA in collagen-induced arthritis (CIA) rats and explored the underlying mechanisms. Methods: Forty-eight female Sprague-Dawley rats were randomly assigned to six groups (*n* = 8): normal control, CIA model, methotrexate (MTX), and CM high-, middle-, and low-dose groups. Anti-inflammatory and joint-protective effects were evaluated using biochemical and histological analyses. To elucidate the mechanisms, we applied metabolomics, network pharmacology, and transcriptomics approaches. Results: The results demonstrated that CM extract effectively suppressed synovial inflammation in CIA rats, reducing joint degradation. CM’s anti-inflammatory effects were mediated through the TNF signaling pathway, modulating glycerophospholipid and amino acid metabolism, including reduced levels of tryptophan, LysoPC, and asparagine. Molecular docking identified scutellarin and apigenin as key bioactive compounds. Additionally, immunofluorescence analysis revealed CM’s therapeutic effects via TNF signaling inhibition and suppression of M1 macrophage polarization. Conclusions: These findings highlight the therapeutic potential of CM for RA and support its development as a functional food or pharmaceutical product.

## 1. Introduction

Rheumatoid arthritis (RA) is a systemic autoimmune disease characterized by alterations in the microenvironment of the articular cavity and the infiltration of B cells, T cells and macrophages [1]. Collectively, these effects result in bone destruction, cartilage erosion, and joint dysfunction. Studies have also indicated that patients with RA are at an increased risk of metabolic syndrome, cardiovascular disease, respiratory conditions, infections, and malignancies compared to the general population [2], significantly reducing their quality of life. Non-steroidal anti-inflammatory drugs (NSAIDs), disease-modifying anti-rheumatic drugs (DMARDs), and glucocorticoids (GCs) are routinely used to prevent the progression of RA [3,4]. However, these strategies are associated with serious side effects, limited efficacy in preventing bone destruction, and the emergence of drug resistance [5]. Consequently, there is an urgent need for more effective and less toxic treatment modalities for RA. Functional foods and herbal supplements have demonstrated efficacy in managing complex diseases with fewer side effects [6], highlighting their potential in RA treatment. Determining the therapeutic effects and mechanisms of herbal supplements could open a new avenue for developing prevention and treatment strategies for RA.

One promising candidate is *Caryopteris mongolica* Bunge (Lamiaceae), an aromatic shrub that grows in the deserts, hill slopes, steppes, and mountainous areas of Mongolia and Inner Mongolia, China [7,8]. Traditionally, the aerial parts of CM have been used to alleviate rheumatoid arthritis (RA), edema, and dyspepsia. It is especially valued by populations in cold and windy regions for its reputed benefits in reducing rheumatic pain when consumed as herbal tea. CM is rich in a variety of biologically active substances, including proteins, flavonoids, phenolic acids and oils, which collectively contribute to its multifaceted nutritional effects. Among its active constituents, flavone glycosides, iridoid glycosides, and diterpenoid glycosides have been isolated [9,10,11]. Studies have highlighted their cholinesterase-inhibitory [12,13], anti-inflammatory, and antioxidant effects [14]. These activities highlight the potential of CM for treating RA. Reports [15,16] indicate that cholinesterase inhibitors increase acetylcholine levels, activating the α7-nicotinic acetylcholine receptor and suppressing pro-inflammatory cytokines such as TNF-α and IL-6, thereby reducing systemic inflammation and joint damage. Concurrently, their antioxidant properties mitigate oxidative stress, a critical driver of RA-related inflammation and tissue damage [17]. In summary, these effects address key pathological features of RA, suggesting that combining cholinesterase-inhibitory activity with anti-inflammatory and antioxidant actions is particularly effective in managing the disease. However, despite its long-standing use in traditional medicine, rigorous scientific investigations into its efficacy and underlying mechanisms in RA management remain scarce. This represents a significant gap in our understanding and highlights the urgent need for research to substantiate its therapeutic potential. As a result, its development potential as a functional food has not been adequately assessed, necessitating further evaluation of its pharmacological constituents and the mechanisms underlying its anti-RA activity.

The complexity of the pathogenesis and contributing factors of RA make it difficult to fully replicate in animal models. The collagen-induced arthritis (CIA) model offers notable advantages for studying RA, as it replicates many key features of the disease, such as symmetrical joint involvement, synovial inflammation, pannus formation, and cartilage and bone destruction [18]. While it may not capture all aspects of human RA, it remains a useful model for understanding the disease. Additionally, the CIA model provides valuable insights into the immune mechanisms underlying RA, particularly the roles of T cells, B cells, and cytokines in the inflammatory process [19]. Given these advantages, the CIA rat model was selected in this study to investigate the therapeutic effects and underlying mechanisms of the treatment, providing a solid platform for evaluating potential RA therapies.

Macrophages play a critical role in the pathogenesis of RA. Over the past decades, studies have shown that macrophages drive the inflammatory response and joint damage in RA by releasing pro-inflammatory factors such as TNF-α, IL-1, IL-6, and matrix metalloproteinases [20]. Macrophages can polarize into M1 (pro-inflammatory) and M2 (anti-inflammatory and repair) phenotypes. In RA, M1 macrophages dominate, leading to persistent inflammation and tissue damage. Therefore, promoting macrophage polarization towards the M2 phenotype has emerged as a potential therapeutic strategy.

To provide new insights into the potential of CM as a functional food or medicinal agent, this study explored the efficacy of CM in CIA rats. Observational parameters included inflammatory cytokine production, organ indices, histological features, and immunofluorescence characteristics. Additionally, the mechanisms of CM’s effects on CIA were investigated using an integrated approach that combined metabolomics, network pharmacology, and transcriptomics analyses, validated through biological techniques. We believe that this approach could significantly enhance our understanding of the mechanisms underlying the CM’s therapeutic effects on RA, thus helping to promote its functional and medicinal applications.

## 2. Materials and Methods

### 2.1. Materials and Reagents

CM was collected from Liang Cheng (Wulanchabu, China) on 8 August 2023, in Mongolia and and identified by Professor Guojing Li. A voucher specimen was deposited at the herbarium of Inner Mongolia Medical University, No. 20230016. Methanol, acetonitrile and formic acid (MS grade) were acquired from CNW Technologies (Shanghai, China). Ammonium acetate and bovine type II collagen solution (CII) was obtained from Sigma Aldrich Co., Ltd. Incomplete Freund’s Adjuvant (IFA) was purchased from Chondrex, Inc (Washington, DC, USA). Methotrexate (MTX) was obtained from Shanghai Shangyao Xinyi Pharmaceutical Co., Ltd. (Shanghai, China) and used as the positive control. Enzyme-linked immunosorbent assay (ELISA) kits for IL-10, IL-17, and TNF-α were obtained from the Servicebio Biotechnology Institute (Wuhan, China).

### 2.2. Network Pharmacology Analysis

The putative targets of components contained in CM was collected from Swiss Target Prediction database (http://www.swisstargetprediction.ch/, accessed on 10 November 2023) (Probability > 0.1) [21], PharmMapper database (http://www.lilab-ecust.cn/pharmmapper/, accessed on 10 November 2023) [22] and SuperPred [23] database (https://prediction.charite.de/, accessed on 10 November 2023) (Probability ≥ 0.9). Before that, the components were prescreened according to pharmacokinetic principles with Swiss ADME database (http://www.swissadme.ch/, accessed on 10 November 2023). “Rheumatoid arthritis” as the search term acquire related targets of RA in Dis Ge NET (https://www.disgenet.org/, accessed on 10 November 2023) (Correlation score > 0.1), Gene Cards (https://www.genecards.org/, accessed on 10 November 2023) (Correlation score ≥ 5), Therapeutic Target Database (TTD, http://db.idrblab.net/ttd/, accessed on 10 November 2023) [24]. Potential targets of CM anti-RA and a compound-target network were then analyzed and constructed with the shared targets of CM and RA. Cytoscape software (https://cytoscape.org/, accessed on 12 December 2023, version 3.9.0) was employed for network visualization. Moreover, enrichment analyses were carried out using DAVID database (https://DAVID.ncifcrf.gov/, accessed on 6 January 2024, version 6.8) and KEGG database to predict the key pathways of CM anti-RA.

### 2.3. Molecular Docking

The crystal structures of NFKBIA (PDB:6y1j), MAP3K7 (PDB:2WWZ), JUN (PDB:6osn), IL-15 (PDB:2z3q), FOS (PDB:6yqn), EDN1 (PDB:1t7h), CXCL12 (PDB:6xds), CXCL10 (PDB:7c7p), CXCL1 (PDB:5n9b), and CCL4 (PDB:2bnu) were retrieved from the PDB database (https://www.rcsb.org/, accessed on 15 November 2023), while the 3D structure of the top 10 components in CM was prepared via PubChem database. Prior to docking, ligand and receptor preparations were conducted with Auto Dock Tools software (Version 4.2.6). The induced-fit docking simulation was performed between the structure of components and targets via Vina procedure (Version 1.2.0) and visualized with PyMOL software (Version 2.6.0). The docked complexes were analyzed for interaction affinities as expressed in kcal/mol.

### 2.4. CM Extraction Preparation and Determination

After collection, the air-dried aerial parts of CM were ground to a fine powder (over 40 mesh screens) and stored at −80 °C until extracted. Distilled water was used to extract CM powder (1:12 *w*/*v*) under refluxed condition for 2 h twice. The extract was freeze-dried to obtain dried powders, giving an extraction yield of 19.2%. The main ingredients of CM extracts were identified by UHPLC-Q-Exactive MS/MS (Thermo Fisher Scientific Inc., Waltham, MA, USA) and listed as Supporting Information Table S1. The stability of the extract was analyzed by using HPLC, as shown in Supporting Information Figure S1, the chemical components in the aqueous extract remained stable for up to 12 h at 4 °C.

### 2.5. Animals

A total of 48 female Sprague-Dawley rats, 6 weeks old with 180–220 g in weight, were purchased from SiPeiFu (Beijing) Biotechnology Co., Ltd. (Approval No. SCXK (Jing) 2019–0008, Beijing, China). All rats were housed two per cage under specific pathogen-free conditions with controlled temperature (20 ± 2 °C), humidity (55 ± 5%) and light/dark cycle (12 h/12 h). The housing cages were equipped with a stainless-steel wire lid, and a bedded floor lined with wood shavings to provide a comfortable and hygienic environment, and were regularly cleaned and sanitized to maintain optimal hygiene conditions. All rats were fed with unlimited water and regular pellet feed. This study were conducted in adherence to the National Institutes of Health Guide for Care and Use of Laboratory Animals and the ARRIVE guidelines for reporting animal research, and project received approval by the Animal Ethics Committee of Inner Mongolia Medical University (approval number: YKD202301198) on 1 March 2023.

### 2.6. Induction, Treatment, and Evaluation of Collagen-Induced Arthritis (CIA) in Rats

**Figure 1:**
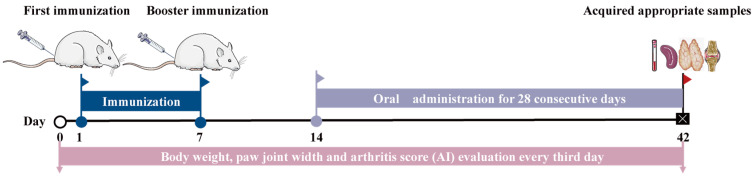
Induction protocol for collagen-induced arthritis (CIA) in SD rats and the drug treatment schedule employed during the experiments.

After a 7-day acclimation period, 48 rats were allocated to six groups (normal control (NC) group, model group (CIA), methotrexate (MTX) group and CM high dose/middle dose/low dose rat groups using a stratified random sampling method based on body weight to reduce bias and ensuring group comparability [25]. Previous research supports that eight rats per group provides adequate statistical power to detect significant effects [26]. As shown in Figure 1, all rats, except those in the control group, were sensitized twice via subcutaneous injection of 0.2 mL Freund’s adjuvant–collagen type II mixture (*v*:*v* = 1:1) on days 0 and 7 to develop the CIA model, following a previously reported protocol [18]. Fourteen days after the first immunization, MTX group rats received treatment with 1 mg/kg/3d of methotrexate, rats in the CM high-dose group (CM-H) received treatment with 5.40 g/kg/d of lyophilized CM extract (equivalent to 28.13 g·kg^−1^·d^−1^ of raw CM), while those in the CM middle-dose group (CM-M) received 2.70 g/kg/d of lyophilized CM extract (equivalent to 14.06 g·kg^−1^·d^−1^ of raw CM). The CM low-dose group (CM-L) received 1.35 g/kg/d of lyophilized CM extract (equivalent to 7.03 g·kg^−1^·d^−1^ of raw CM). The dosages for the high-, middle-, and low-dose groups were established based on 4 times, 2 times, and 1 time the clinical human dose, respectively. During the same period, NC group animals were administered with the same volume of saline. All treatments were performed once daily for 28 consecutive days.

Body weight, paw joint width and arthritis score (AI) were then evaluated by two independent blinded operators every 3 days from day 0. Arthritis severity was scored using a comprehensive scoring system [27], including inflammation, pannus, synovial vessel density (angiogenesis), cartilage four aspects. Each aspect was scored on a scale ranging from 0 (indicating no lesion) to 3 (indicating severe involvement). The total score represents the cumulative sum of these individual categories, thereby providing an extensive measure of joint damage and inflammation in CIA rats. To control for confounding factors in observational data, methods include randomization, blinding, counterbalancing treatment order, environmental control to adjust for potential confounders.

At the termination date (day 42), the rats were fasted overnight and sacrificed by administering an overdose of pentobarbital via intraperitoneal injection. Appropriate samples were then acquired for further analyses.

Any animal exhibiting signs of severe distress or illness or rapid weight loss of more than 20% compared to the rat’s normal body weight would have been euthanized according to the guidelines set by the Institutional Animal Care and Use Committee (IACUC). However, no animals in this study required early euthanasia.

### 2.7. Measurement of Inflammatory Cytokine Production and Organ Index

Blood samples were collected from all rats at sacrifice, as described earlier. Serum was separated by centrifugation at 3500 rpm for 10 min at 4 °C and frozen at −80 °C to await further analysis. The serum levels of inflammatory cytokine, including IL-10, IL-17 and TNF-α, were then determined by ELISA in accordance with the manufacturer’s instructions.

After blood samples were acquired, the spleen and thymus were excised, followed by rinse with normal saline and weighing. Organ index was calculated based on the ratio of organ weight to body weight.

### 2.8. Histological and Immunofluorescence Analyses

Prior to paraffin embedding, all ankle joints were separated and fixed overnight in 4% paraformaldehyde at 4 °C. The samples were decalcified in 14% EDTA for 14 days and subsequently embedded in paraffin. Paraffin sections (4 µm) were prepared and stained with hematoxylin and eosin (H&E) dye and safranin O dye to evaluate lesions. Histopathological score [28] was assessed blindly based on the severity of arthritis in four aspects, including cell infiltration, synovial hyperplasia, pannus formation, cartilage erosion. The graded score for each feature was evaluated using a range of 0–3, and histopathological score was the sum of the four points.

Immunofluorescence microscopy was performed to analyze CD206 and iNOS expression. Briefly, the bone sections were incubated with antibodies specific for CD206 (1:200), and iNOS (1:200) at 4 °C overnight. Afterwards, bone sections were then probed with secondary antibodies conjugated with fluorescence at roomtemperature for 50 min, stained using DAPI for 10 min. The sections were then visualized using a Leica TCS SP8 confocal microscope (West Hollywood, CA, USA).

### 2.9. Metabonomic Analysis

Methanol-acetonitrile (1:1 (*v*/*v*)) mixture with deuterated internal standards (200 μL) was added to 50 μL serum. After vortexed for 0.5 min, all samples were sonicated in 4 °C water bath for 10 min, and incubated at −40 °C for 1 h. Subsequently, the samples were centrifuged at 12,000 rpm (RCF = 13,800 × g, R = 8.6 cm) for 15 min at 4 °C to precipitate proteins. The supernatant was collected for UHPLC-Q-Exactive MS/MS analysis. To ensure the accuracy and consistency of our analysis, quality control (QC) sample was prepared and analyzed according to previous report [29].

UHPLC-Q-Exactive MS/MS analyses were performed on a Vanquish UHPLC system (Thermo, USA) coupled with an Orbitrap Exploris 120 mass spectrometer (Thermo, USA). A Waters ACQUITY UPLC BEH Amide column (2.1 mm × 50 mm, 1.7 μm) was used for the separation with a flow of 0.3 mL/min. The mobile phase consisted of 25 mmol/L ammonium acetate and 25 mmol/L ammonia hydroxide in water (pH = 9.75) (A) and acetonitrile (B). For each sample, we injected 2 µL for analysis. MS/MS analysis was performed in information-dependent acquisition mode. Sheath gas flow rate was set to 50 Arb, Aux gas flow rate was 15 Arb. Spray voltage was 3.8 kV for positive ion mode and −3.4 kV for negative ion mode. The capillary temperature was maintained at 320 °C for both ion modes. The spray voltage was set at 300 V, and the collision energy was SNCE 20/30/40 for both ion modes. Data were collected in centroid mode and the mass range was 100–1100 *m*/*z* for full MS with resolution of 60,000, 100–1100 *m*/*z* for MS/MS with resolution of 15,000.

Original data files were independently converted to mzXML format using ProteoWizard software (Alto, Cumberland, RI, USA, Version 3.0.4472). The data were then pre-processed using an in-house program for peak extraction, peak alignment, and automatic integration. Then an in-house MS^2^ database (BiotreeDB, Version 2.1) was applied to identify feature metabolites. A multi-dimensional peak tables containing sample name, peak number, and normalized peak area, were then put into the SIMCA 16.0.2 software (MKS Data Analytics Solutions, Sweden) for multivariate statistical analysis. Before analyzing, the data were scaled and log-transformed based on previously established methods [30,31]. Here, the potential outliers in the dataset were identified with principal component analysis (PCA), significantly changed metabolites between two groups were underlying by orthogonal partial least squares discriminant analysis (OPLS-DA) [32]. We then identified differential metabolites according to Variable importance in projection (VIP > 1), and *p*-value (*p* < 0.05). In addition, commercial databases including KEGG (http://www.genome.jp/kegg/, accessed on 15 November 2023, Version 2.6.0) and MetaboAnalyst (http://www.metaboanalyst.ca/, accessed on 15 November 2023, Version 4.0) were used to construct the comprehensive metabolic network [33].

### 2.10. Transcriptional Sequencing and Bioinformatics Analysis

According to the manufacturer’s instructions, after ankle joints were grinded into powder under liquid nitrogen, Trizol reagent (Invitrogen) was added to each sample to extract total RNA. After purity was checked with kaiaoK5500^®^Spectrophotometer (Kaiao, Beijing, China), RNA was quantified using using the RNA Nano 6000 Assay Kit of the Bioanalyzer 2100 system (Agilent Technologies, Santa Clara, CA, USA). VAHTS Universal V6 RNA-seq Library Prep Kit for Illumina were used and then sequenced on the Illumina Hiseq4000 platform to obtain 150-bp paired-end reads. The sequencing data were filtered based on the length, purity, quality and N base percent of reads with Perl script. Then, HISAT2 (Version 2.1.0) was applied to align the clean reads. Differential genes were screened and analyzed with the DESeq with threshold of FC value > 1.5, and *p* < 0.005. KEGG and GO enrichment analysis were used to identify the anti-RA related signaling pathways.

### 2.11. RNA Isolation and Real-Time Polymerase Chain Reaction (PCR)

The total rat ankle joint RNA were extracted using TRIzol^TM^ reagent according to the manufacturer’s instructions. Extracted RNA was then reversely transcribed into cDNA with SweScript All-in-One RT SuperMix. Real-time PCR was then performed using Universal Blue SYBR Green qPCR Master Mix and run on a Real-Time PCR System (CFX Connect, Bio-Rad, Hercules, CA, USA) with the primers listed in the online Supplemental Table S2. RNA levels were normalised with GAPDH.

### 2.12. Statistical Analysis

Sample size calculation: Previous and preliminary data [34,35] indicate that to obtain significance at the α = 0.05 at statistical power 0.9, n = 8 animals pre group are necessary to detect difference in vocalizations using GraphPad Prism 8 (GraphPad Software, SanDiego, CA, USA). Data were described as the mean ± standard deviation (SD) unless indicated elsewhere. Data conforming to Gaussian distribution were statistically analyzed with two-tailed Student’s t-test or one-way analysis of variance (ANOVA) followed by Bonferroni post hoc test; otherwise, the nonparametric Kruskal–Wallis one-way rank-sum was employed. All statistical analysis were performed with SPSS 20.0 software and a *p* value ≤ 0.05 was regarded as statistically significant.

## 3. Results

### 3.1. Inflammatory-Response-Related Biological Processes Involved in CM on Anti-RA Process

**Figure 2:**
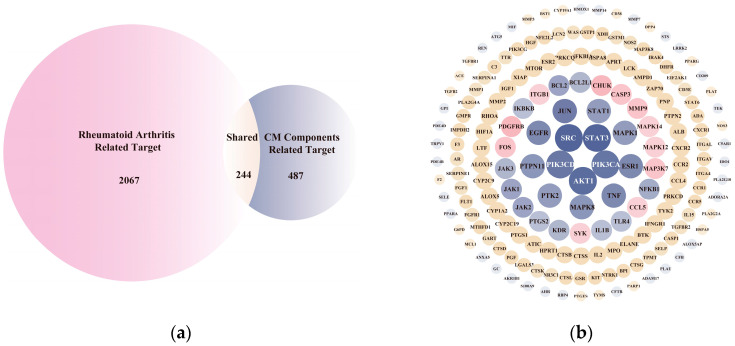

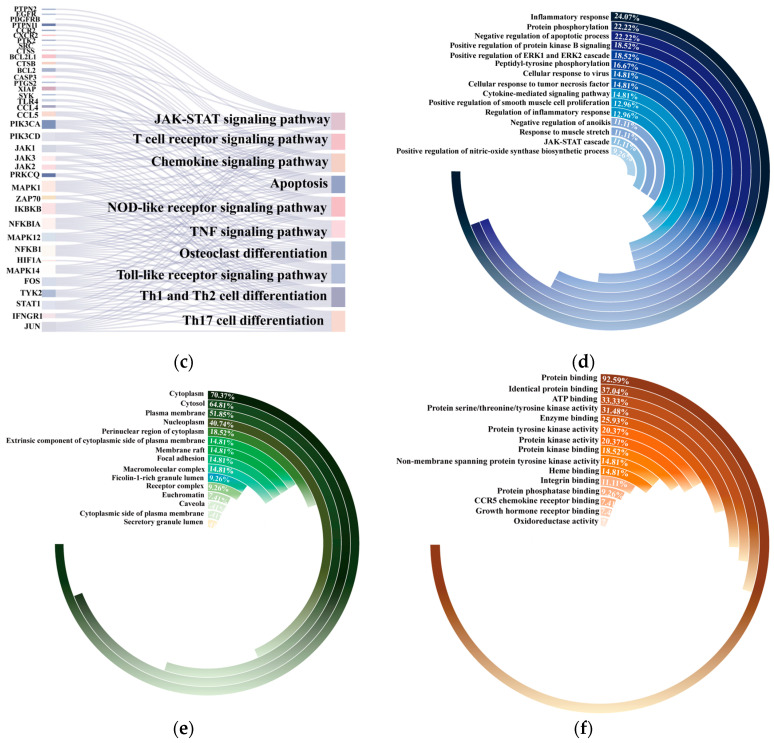
Network analysis of CM in treating rheumatoid arthritis. (**a**) Venn diagram showing 244 common targets between CM and rheumatoid arthritis. (**b**) STRING network visualization of the 244 common targets with topological analysis. (**c**) The top 10 significantly enriched terms in KEGG pathways. (**d**) Top 15 significantly enriched terms in biological processes. (**e**) Top 15 significantly enriched terms in cellular components (**e**). (**f**) Top 15 significantly enriched terms in molecular functions.

Network pharmacology is a multidimensional pharmacological field, interpreting the interrelation of targets and plant phytochemicals to body disorders [36]. Hence, we acquired the active targets and components from CM in its positive effect on anti-RA using network pharmacology. Prior to prediction of potential anti-RA targets and pathways of CM, compounds in CM needed to be identified first. A UHPLC-Q-Exactive MS/MS was employed to analyze the ingredients of CM, where a total of 183 components were found (Table S1). A total of 77 components were chosen for further investigation based on the ADME principles, and 731 putative gene targets related to these 77 phytochemicals were identified using the Swiss Target Prediction, PharmMapper, and SuperPred databases. Then, 2311 unique RA-related gene targets were predicted after removal of duplicates from disease target database. A total of 244 potential targets for CM against RA were performed with the intersection of CM-related targets and RA-related predicted targets (Figure 2a). Detailed information on the above putative targets is provided in Tables S3–S5. After protein–protein interaction (PPI) construction and hub network analysis with Cytoscape software (Figure 2b), 54 core candidate nodes, whose NCC, DMNC, MNC and degree were greater than the median, were identified as key targets for CM anti-RA (Table S6). The enrichment of KEGG signaling pathways demonstrated that Th17 cell differentiation, Th1 and Th2 cell differentiation, Toll-like receptor signaling pathway, TNF signaling pathway, ect, were involved in the CM treatment of RA (Table S7 and Figure 2c). Furthermore, GLUE GO analysis identified the top biological features of our interaction network as peptidyl-tyrosine phosphorylation, negative regulation of anoikis, and inflammatory response (Table S7 and Figure 2d). The molecular functions were associated with protein serine/threonine/tyrosine kinase activity, protein tyrosine kinase activity, enzyme binding, etc., (Table S7 and Figure 2e). Additionally, the cellular components showed correlation with cytoplasm, extrinsic component of cytoplasmic side of plasma membrane, and cytosol (Table S7 and Figure 2f). The above results lay the foundation for further mechanism verification.

**Figure 3:**
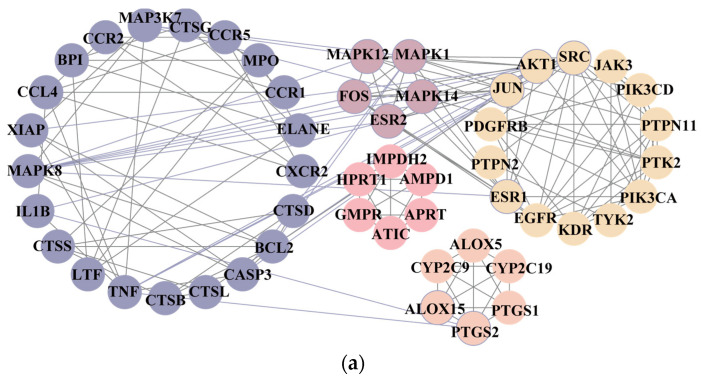

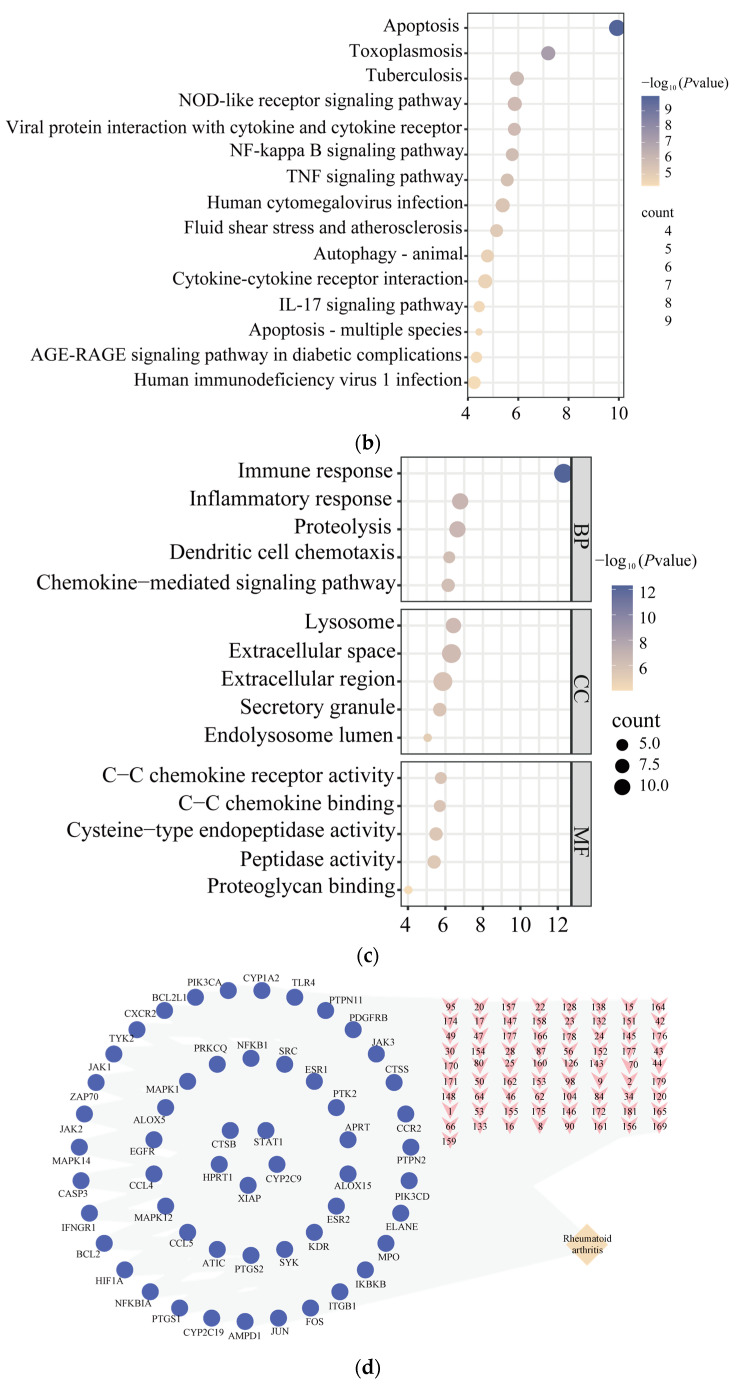
(**a**) Derived different MCODE clusters: Light yellow nodes were kernel genes in MCODE cluster1; Light orange nodes were kernel genes in MCODE cluster2; Pink nodes were kernel genes in MCODE cluster3; Light purple nodes were kernel genes in MCODE cluster4; Light blue nodes were kernel genes in MCODE cluster5. (**b**) The top 15 significantly enriched terms of MCODE cluster5 in KEGG pathways. (**c**) The top 5 enriched terms of MCODE cluster5 in biological processes (BP parts), cellular components (CC parts), and molecular functions (MF parts). (**d**) Illustration of the relevance among components of CM, the key targets, and diseases. Yellow nodes refer to RA; Pink nodes refer to the components contained in CM; Blue nodes refer to the key targets.

To further explore the underlying mechanism, we employed the MCODE algorithm to construct a modular network and identify the core therapeutic targets. With this algorithm, five highly correlated clusters were generated (Table S8 and Figure 3a). In order to elucidate the functional roles of these targets within different clusters, we performed functional enrichment analyses using DAVID database. The enrichment analyses showed that these clusters were associated with diverse biological processes, cellular components, molecular functions, and KEGG signaling pathways (Table S9). Moreover, Cluster 5 exhibited specific enrichment related to immune and inflammatory response (particularly inflammatory response) (Table S9 and Figure 3b,c). The interaction network diagram (Figure 3d) between components and 54 core candidate was constructed using Cytoscape software to identify the main active compound related to anti-RA. Caryopincaolide L, scutellarein, rhamnetin, apigenin, diosmetin shared most common target genes with RA, leading to their selections for further analysis. The structural information of these main active components is listed in Table 1.

### 3.2. CM Alleviated Symptomatic Progression in CIA Rats

**Figure 4:**
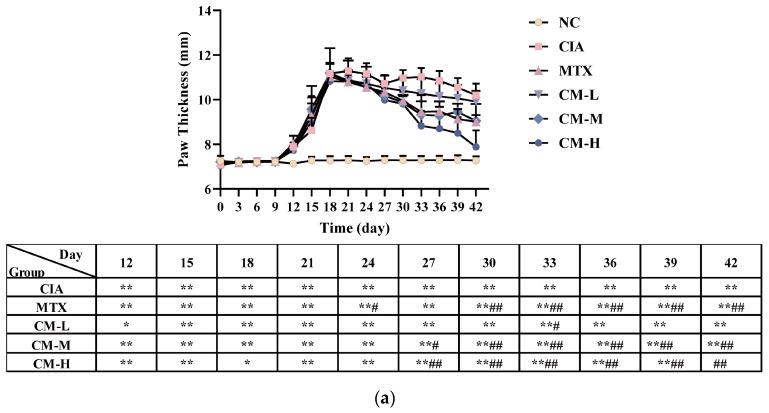

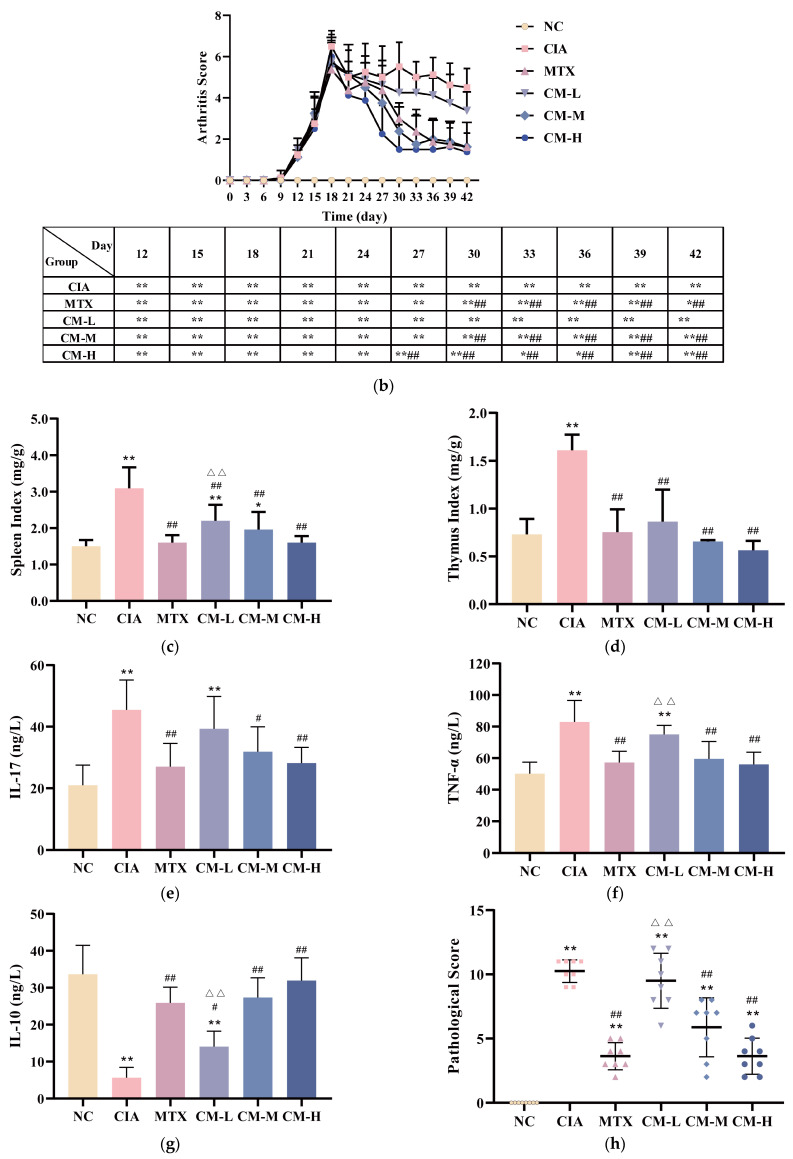

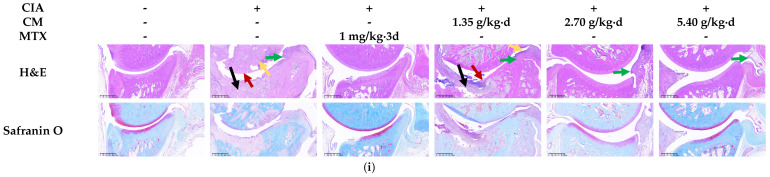
Alleviating effects of CM on CIA rats. (**a**) paw thickness, (**b**) AI scores (**c**) spleen index, (**d**) thymus index, (**e**) IL-17, (**f**) TNF-α, (**g**) IL-10, (**h**) pathological scores of H&E and safranin O/fast green staining, (**i**) H&E staining and safranin O/fast green staining. multiple comparisons test (The red arrow shows cartilage erosion, the black arrow shows inflammatory infiltration, the green arrow shows synovial proliferation, and the orange arrow shows pannus formation). All values were presented as mean ± SD, *n* = 8 per group. * *p* < 0.05 and ** *p* < 0.01 vs. NC group, ^#^*p* < 0.05 and ^##^*p* < 0.01 vs. CIA group, ^∆^ *p* < 0.05 and ^∆∆^ *p* < 0.01 vs. MTX group.

Based on the experimental conditions shown in Figure 4a, the initial symptoms of hind paw swelling and elevated AI scores were observed on Day 12 in the CIA group (Figure 4a,b). Both indices continued to increase, reaching their peak on Day 18. As shown in Figure 4c,d, the immune organ in the CIA group exhibited severe damage for their index increasing. Analysis also revealed that there was a significant increase in IL-17 (*p* = 0.003), and TNF-α (*p* = 0.011), along with a significant reduction in IL-10 (*p* = 0.000) in the CIA group (Figure 4e,f). Apparently, the overall histological and pathological scores demonstrated significant arthritic manifestations-for example, inflammatory cell infiltration, synovial cell hyperplasia, cartilage erosion and pannus formation-were concomitantly observed in CIA rats when compared to NC rats (Figure 4h,i). Collectively, these results indicate that Freund’s adjuvant-collagen type II mixture may promote inflammation and bone erosion in CIA rats.

Encouragingly, significant reductions in paw swelling, immune organ indices, inflammatory cytokine levels, and articular cartilage damage were observed in the treatment groups (Figure 4a–i). All treatment groups, except for the CM-L group, showed remarkable improvements in autoimmune and inflammatory joint parameters. Among them, significant decline in paw swelling, AI scores, immune organ index, IL-17, and TNF-α levels, increase in value of IL-10 was documented. Histological and pathological parameters, regarding cell infiltration, synovial hyperplasia, pannus formation, and cartilage erosion, were turned over in these treatment groups as shown in Figure 4h–i. Moreover, the marked reductions in pathological scores further indicated a significant reversal of arthritic conditions in the CM and MTX groups compared to the CIA rats. One delightful indication arising from our findings was that CM may provide a beneficial therapeutic effect in CIA. At the end of the experiment, histopathological observations of the heart, liver, and kidney tissues in all groups revealed no abnormal changes, confirming the safety and non-toxicity of CM during the treatment period. Representative pathological morphologies are provided in Figure S2.

### 3.3. CM Rebalanced Metabolism by Promoting Glycerophospholipid and Amino Acid Turnover in CIA Rats

The composition and concentration of metabolites may change during RA progression. To screen the targeted therapeutic pathways that are closely related to RA, we used metabolomics to uncover the metabolic change in this study. Prior to analysis, the quality and reliability of metabolomics data were assessed using IS’s retention time and peak area deviations, PCA analysis, and correlation analysis. As shown in Table S9, the *RSD*s of six ISs were 0.68~3.30% for peak area, suggesting precision could be satisfactory with the acceptable range. Points aggregation of PCA model and correlation coefficient > 0.9 for QC samples also indicated a good reproducibility and reliability of the process (Figure S3).

**Figure 5:**
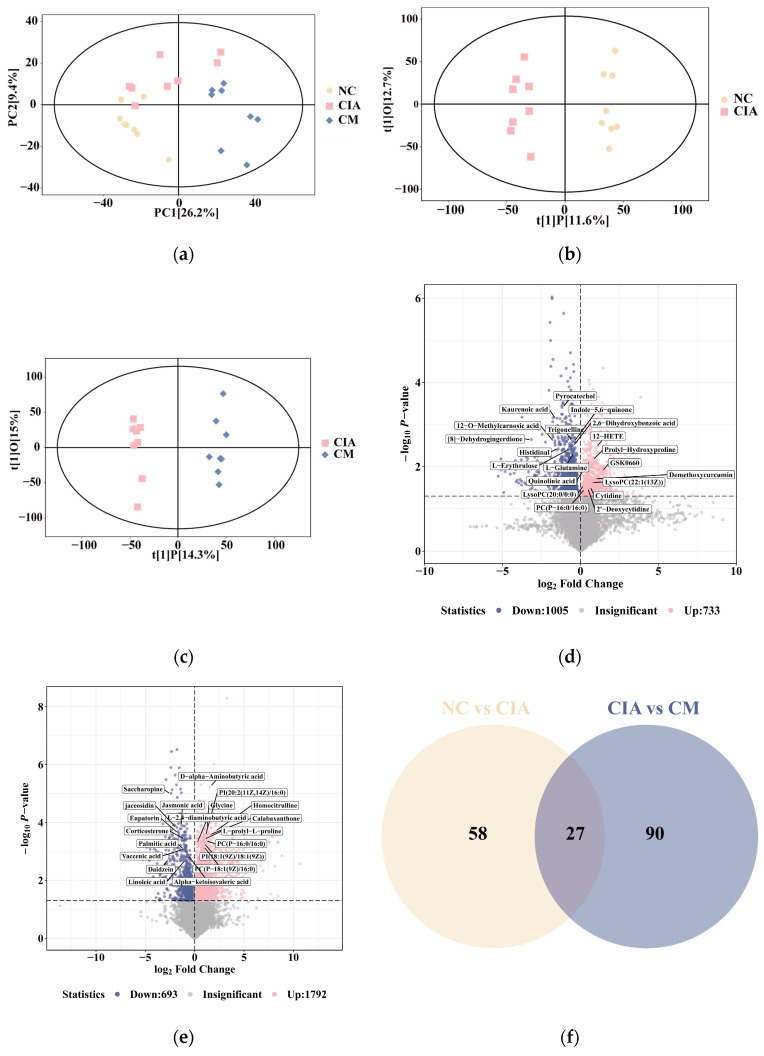

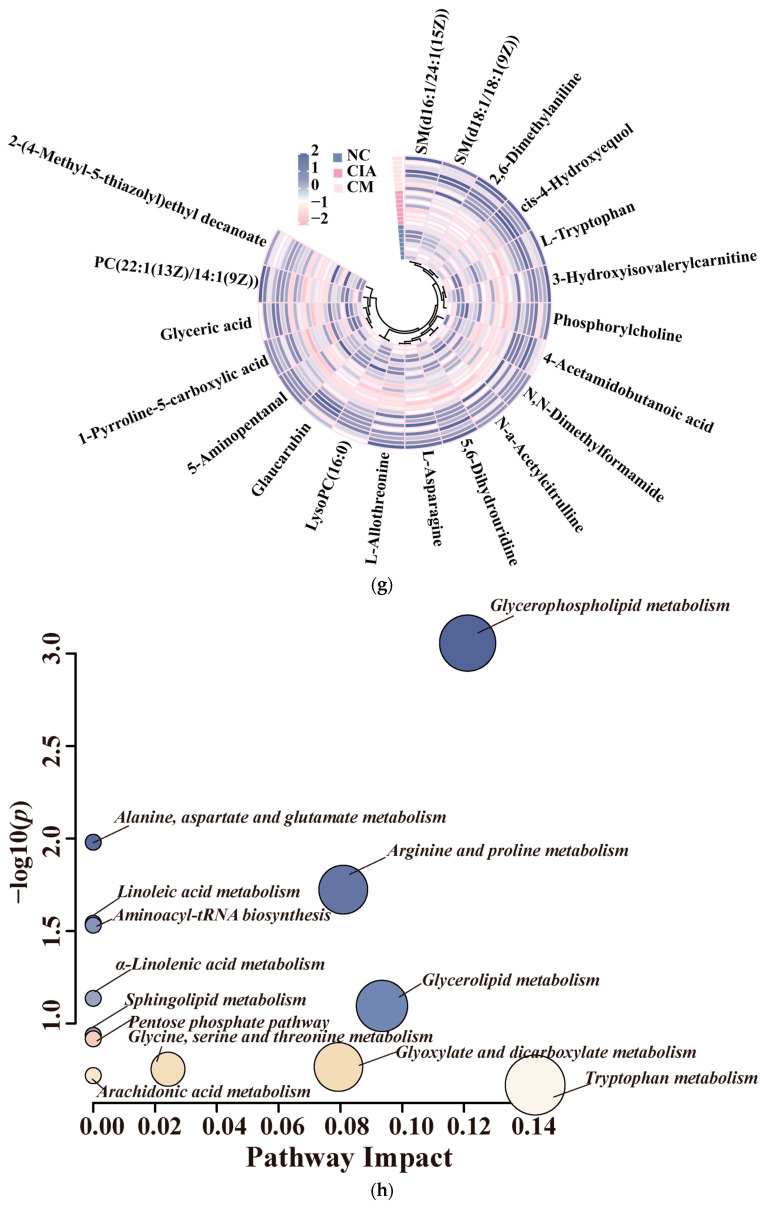

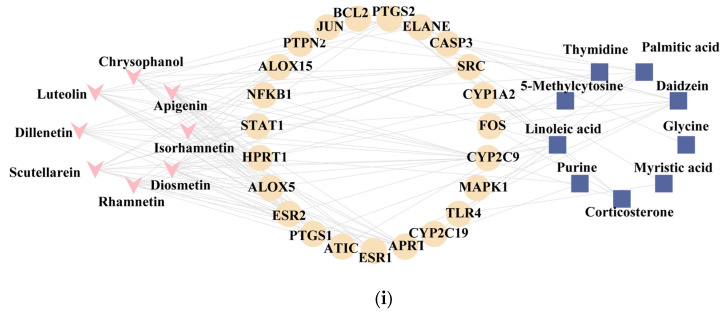
Multivariate analysis based on the UHPLC-Q-Exactive MS/MS profiling data: (**a**) Score scatter plot of PCA model for three different group; (**b**) OPLS-DA score plot for group NC vs. CIA; (**c**) OPLS-DA score plot for group CIA vs. CM; (**d**) Volcano plot for group NC vs. CIA; (**e**) Volcano plot for group CIA vs. CM; (**f**) Venn diagram of 27 common metabolites of CIA vs. CM and NC vs. CIA. (**g**) Heatmap of hierarchical clustering analysis for three different group. (**h**) Summary of ingenuity pathway analysis with MetaboAnalyst. (**i**) Illustration of the relevance among components of CM, the key targets, and metabolites. Pink nodes refer to the components contained in CM; Yellow nodes refer to the key targets; Blue nodes refer to metabolites.

The metabolic profile of the PCA analysis for the CIA group was separated from that of the other groups, showing that metabolites in rats were affected by sensitizer agent or dietary supplement (Figure 5a). The observed intragroup variability in the PCA results was likely attributed to individual differences among the rats. To further explore the differences between groups, an OPLS-DA model was used for pairwise comparisons, and the score scatter plots for the respective group pairs are illustrated in Figure 5b,c. To avoid overfitting, 200 permutation tests were performed during the modeling process. The models demonstrated appropriate goodness-of-fit values with high cross-validation predictability (Table S10 and Figure S4). Specifically, the NC vs. CIA mode achieved cumulative R^2^Y values of 0.977 and cumulative Q^2^ values of 0.591, while the CIA vs. CM mode achieved an R^2^Y value of 0.991 and a cumulative Q^2^ value of 0.753, indicating reliable differentiation between the groups. After filtering with VIP > 1 and *t* < 0.05, 1738 candidate differential metabolites (733 up-regulated and 1005 down-regulated metabolites) were obtained between NC group and CIA group (Figure 5d), otherwise, 2485 potential differential biomarkers (1792 up-regulated and 693 down-regulated metabolites) were identified in the CM group compared to CIA group (Figure 5e). Based on the above candidate differential metabolites data, we used HMBD online database and the self-established database to determine the endogenous differential metabolites.

To further investigate the disease-associated metabolites reversed with CM, a Venn diagram of NC vs. CIA and CIA vs. CM was constructed. A total of 27 metabolites were identified as being related to CM anti-RA (Figure 5f). Information on 20 of these rebalanced metabolites following CM treatment is listed in Table S11. Similarly, the clustering heatmap in Figure 5g shows that the elevation of 20 metabolites in CIA group were rebalanced to normal with CM administration. Notably, these metabolites involved in RA-associated metabolic pathways (Figure 5h), including glycerophospholipid metabolism, alanine, aspartate and glutamate metabolism, glycine, serine and threonine metabolism, etc., which was consistent with the previous observation. Collectively, these findings indicate that CM might play a suppressive role in anti-RA via ameliorating the glycerophospholipid and amino acid metabolism. To further investigate the interaction between core target illustrated in network pharmacology research and metabolites changed during CM treatment, we established the construction of a “component-gene-metabolite” network comprising 8 components, 23 genes, and 9 differential metabolites. The integrated analysis revealed that CM regulates metabolic processes through *ALOX15*, *FOS*, *JUN*, and other 20 key targets. (Figure 5i).

### 3.4. Inflammatory-Related Transcriptional Profiles Respond to CM Treatment in CIA Rats

**Figure 6:**
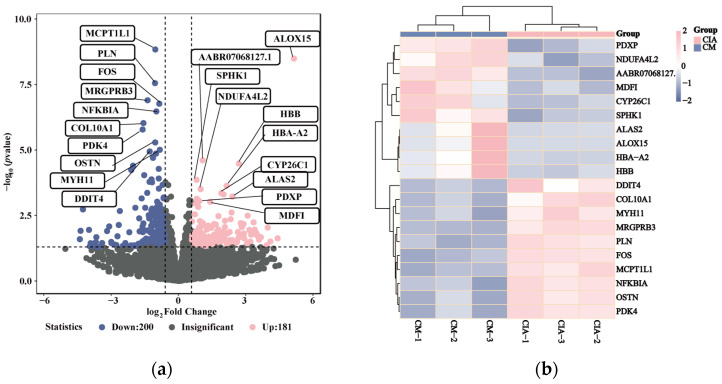

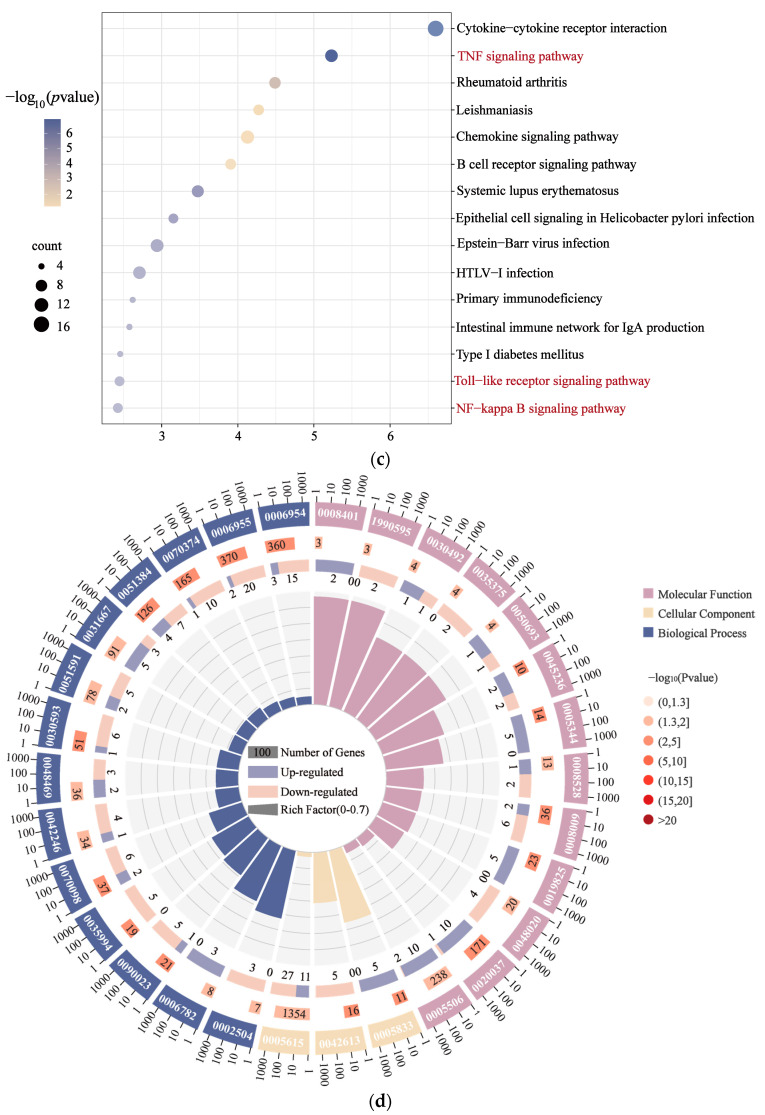
Transcriptomic landscape of CM treating rheumatoid arthritis: (**a**) DEGs volcano plot; (**b**) DEGs heatmap. (**c**) The top 15 significantly enriched terms in KEGG pathways. (**d**) The top 5 enriched terms in biological processes (blue parts), cellular components (yellow parts), and molecular functions (pink parts).

To address the role of CM in the transcriptional mechanism in anti-RA, we performed transcriptome sequencing of non- or CM-treated CIA rats to compare transcription profiles during CM treatment. This analysis resulted in 381 distinct expressed genes between non- or CM-treated CIA rats, with 181 detect differentially expressed genes (DEGs) up-regulated gene and 200 DEGs down-regulated gene in CM group with comparison of CIA group (Figure 6a). Analysis of the gene-expressing clusters demonstrated that CM-treated CIA rats express significantly distinct levels of the inflammatory-related genes compared to CIA rats, such as ALOX15, FOS, NFKBIA (Figure 6b). Our analysis of KEGG pathway enrichment analysis of the gene signature indicated a significant enrichment of cytokine–cytokine receptor interaction, TNF signaling pathway, Toll-like receptor signaling pathway, NF-kappa B signaling pathway (Figure 6c). In addition, the GO enrichment analysis of the DEGs indicated a significant enrichment of chemokine-mediated signaling pathway, inflammatory response, response to interleukin-6 in CM-treated CIA rats (adj. *p* value < 0.05) compared to non-treated CIA rats (Figure 6d).

### 3.5. CM Exhibited a Down-Regulatory Effect on Inflammatory-Related Gene Expression with Its Active Components

**Figure 7:**
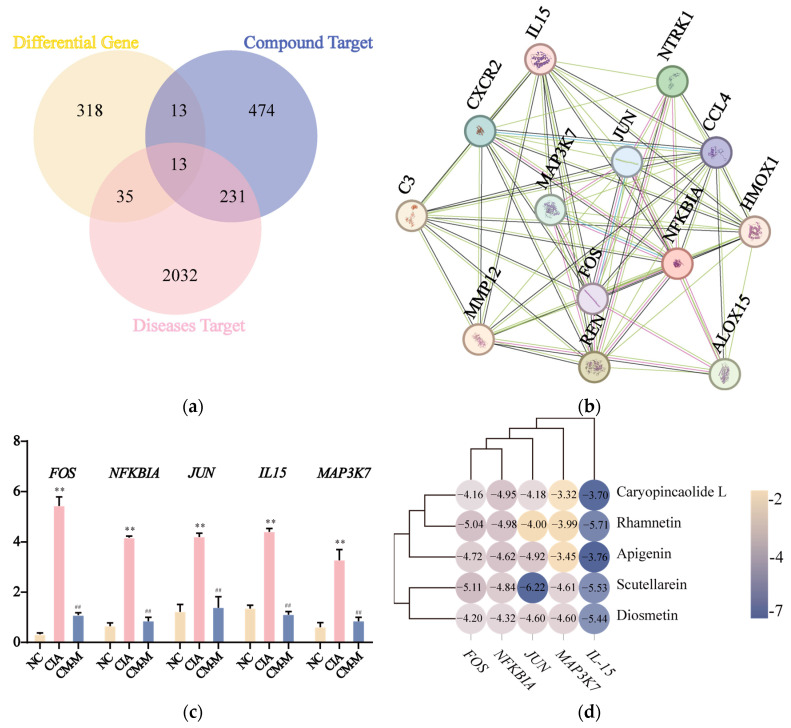

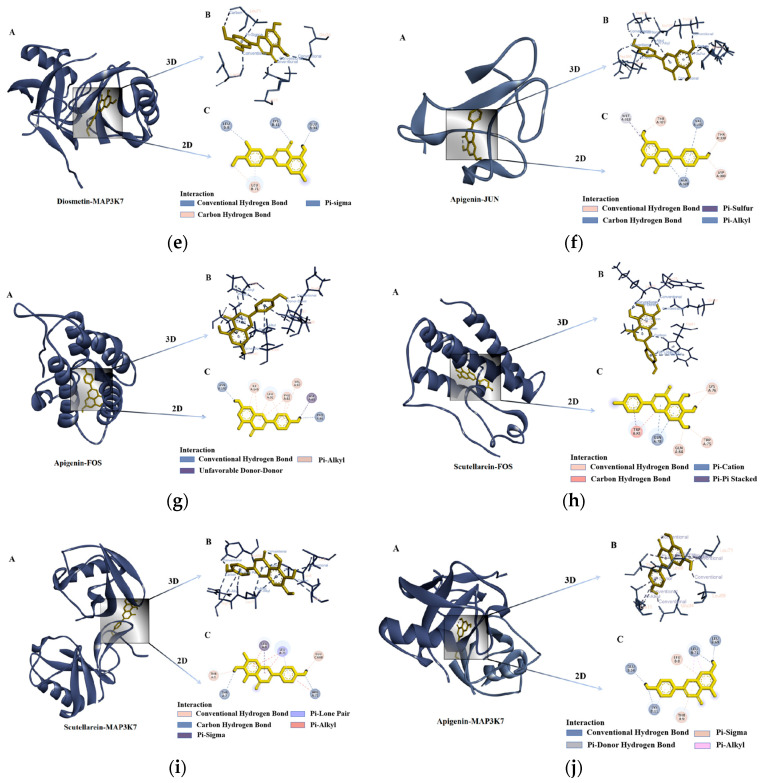
(**a**) Venn diagram of common targets between CM, rheumatoid arthritis and DEGs. (**b**) 13 common targets via STRING network topological analysis. (**c**) Fluorescence quantitative PCR verification of core differential expressed gene. Molecular docking results, * *p* < 0.05 and ** *p* < 0.01 vs. NC group, ^#^ *p* < 0.05 and ^##^ *p* < 0.01 vs. CIA group: (**d**) The heatmap of docking scores of key targets combining to 5 active compounds in CM. (**e**–**j**) The representative docking complex of key targets and compounds.

Given the evidence as mentioned above, we conducted a Venn analysis involving compound targets, disease targets, and DEGs. The analysis revealed 13 common genes among these three datasets (Figure 7a), indicating that these genes may play a crucial role as differentially expressed genes in the treatment of RA using CM. Subsequently, we constructed a PPI network for these 13 common genes (Figure 7b), which identified *FOS*, *NFKBIA*, *JUN*, *IL-15*, and *MAP3K7* as the top five genes with the highest degree values. We hypothesized that CM may exert its inhibitory effects on inflammatory response in CIA rats by regulating the expression of these genes. As depicted in Figure 7c, *FOS*, *NFKBIA*, *JUN*, *IL-15*, and *MAP3K7* were significantly upregulated in the CIA group compared to the NC group but downregulated under CM treatment.

To further validate the interaction of the aforementioned targets and components, molecular docking technology was employed to assess the binding affinity between these core components and targets. Binding energy was used to evaluate the docking results. As shown in Figure 7d, all the binding energies were less than 0 kcal/mol, indicating spontaneously binding occurred between ligand and receptor. To observe the interaction of small molecules with targets, docking results with better binding energy (less than −5 kcal/mol) were visualized (Figure 7e–j). Hydrogen bonding is the main binding force between small molecules and targets. For diosmetin, apigenin, scutellarein, all targets formed hydrogen bonds with groups on the A/B/C ring.

### 3.6. CM Induced M2 Macrophage Polarization In Vitro

**Figure 8:**
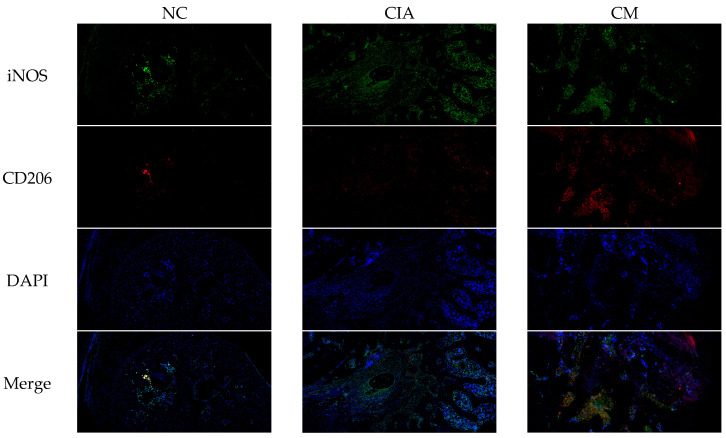
Immunofluorescence double staining of M1 and M2 macrophages in rats’ joint tissues of each group (iNOS as the positive marker for M1 macrophages with green color, CD206 as the positive marker for M2 macrophages with red color, DAPI as the positive marker for cell nuclei with blue color, and Merge as the combined marker of all three).

Macrophage polarization has been identified as a critical factor in inflammation, with the transition from M1 to M2 phenotype being associated with attenuation of inflammatory responses. The above findings indicate that CM may possess anti-inflammatory properties in vivo. With this in mind, we conducted an investigation into the impact of CM on macrophage phenotype in vivo using CIA rats. Specifically, the macrophage subpopulations were characterized using immunofluorescence staining for molecular markers iNOS (M1 marker) and CD206 (M2 marker) [37]. Our results demonstrated that CM treatment led to a decline in iNOS expression and elevation in CD206 expression (Figure 8). The significant difference between CM group and CIA group (n = 3) was observed in iNOS and CD206 cells number (Figure 8). The combined evidence demonstrates that CM is able to inhibit M1 macrophage infiltration and promotes M2 macrophage polarization.

## 4. Discussion

The present study elucidates the therapeutic potential of CM in mitigating RA symptoms using a collagen-induced arthritis (CIA) rat model. The CIA model replicates key immunological and pathological features of human RA, such as synovial inflammation and cartilage destruction, making it a robust platform for investigating RA pathogenesis and evaluating potential therapeutics. Using diverse clinical and histopathological metrics, we demonstrated that CM markedly reduces paw swelling, arthritis scores, and joint destruction, highlighting its potential to impede RA progression and preserve joint integrity.

Although the etiology and pathogenesis of RA have remained elusive, research suggests that the interplay between bone and inflammation establishes a causal relationship [38]. Cytokines are key mediators of immune responses in RA, and our study highlights the ability of CM to modulate their levels. We observed that CM treatment significantly decreased the levels of pro-inflammatory cytokines TNFα and IL-17 while increasing the concentration of the anti-inflammatory cytokine IL-10. This shift in cytokine balance suggests that CM inhibits the secretion of pro-inflammatory mediators and promotes anti-inflammatory responses. TNF-α, IL-17, and IL-10 are central players in RA’s immune regulation [39]: TNF-α, a primary proinflammatory cytokine, induces the secretion of VCAM-1 and ICAM-1 and stimulates the production of IL-6, IL-8, and GM-CSF [40], while IL-17 exacerbates inflammation and bone erosion by promoting bone resorption and vascular opacity [41]; in contrast, IL-10 modulates B cell functions and antibody production, with its in vivo concentration negatively correlated with the susceptibility and severity of RA [42]. And CM’s effects on these cytokines are likely a major mechanism behind its therapeutic potential.

At the metabolic level, CM’s therapeutic effects were closely tied to its ability to restore amino acid and glycerophospholipid metabolism. Disruptions in these pathways are hallmarks of RA and are implicated in immune regulation and inflammatory responses. For instance, reduced LysoPC levels are associated with heightened IL-6 secretion and disease severity [43,44], while tryptophan depletion exacerbates inflammation via increased cytokine production [45]. Methionine inhibits inflammatory and oxidative stress factors, while low levels worsen RA [46]. Arginine, a nitric oxide precursor, is depleted during RA progression [47]. Our study found that CM treatment reversed these metabolic alterations, elevating LysoPCs and normalizing tryptophan and methionine levels, thereby suppressing inflammation and promoting immune homeostasis. These metabolic changes are further linked to CM’s modulation of inflammatory cytokines, as evidenced by decreased TNF-α and IL-17 levels and elevated IL-10 concentrations [48], which collectively ameliorated systemic inflammation.

Transcriptomic analysis further revealed that CM exerts its anti-inflammatory effects by downregulating key genes in the TNF signaling pathway, including *FOS*, *NFKBIA*, *JUN*, *MAP3K7*, and *IL-15*, which are critical in driving inflammatory cascades and tissue damage in RA. Specifically, c-FOS and c-JUN, members of the AP-1 family [49,50], facilitate the expression of pro-inflammatory cytokines in response to TNF-α, while MAP3K7 regulates both the NF-κB and MAPK pathways, contributing to immune dysregulation [51]. IL-15 exhibits diverse biological functions, including the regulation of tissue repair and modulation of inflammatory responses. Elevated IL-15 expression in RA patients exacerbates the inflammatory response [52], thereby contributing to disease progression. CM’s modulation of these genes was confirmed by PCR and further supported by molecular docking analysis, which showed strong binding affinities between CM’s active constituents and these targets. These findings suggest that CM mitigates inflammation by directly disrupting TNF signaling pathways. Specifically, the downregulation of these genes reduces the production of pro-inflammatory mediators derived from glycerophospholipids and shifts amino acid metabolism to favor anti-inflammatory processes [53]. These changes help modulate immune cell activity, reduce inflammation, and promote tissue repair in RA.

Additionally, our findings highlight CM’s role in macrophage polarization, a critical factor in RA pathogenesis [54]. CM suppressed M1 macrophage activation, characterized by pro-inflammatory cytokine production, and promoted M2 macrophage transition, which facilitates tissue repair. This dual effect underscores the comprehensive immunomodulatory properties of CM, further validated by its impact on TNF pathway genes.

Notably, the principal anti-RA constituents identified in CM via network pharmacology exhibit robust binding affinity to critical genes within the TNF pathway. Many of these constituents exert their effects by targeting pathways associated with inflammation and restraining the release of inflammatory mediators. For instance, isorhamnetin demonstrated the ability to attenuate the expression levels of NF-κB and p38 mRNA, thereby inhibiting the release of TNF-α, IL-6, and IL-8 through the NF-κB and p38 MAPK signaling pathways, ultimately ameliorating clinical symptoms in RA mice [55]. Recent investigations by Liu [56] unveiled that isorhamnetin can additionally suppress the overexpression of MMP2 and MMP9 by downregulating the phosphorylation level of key proteins in the SRC/ERK/CREB pathway, thus manifesting anti-RA properties. Likewise, apigenin manifests inhibitory actions on synovial hyperplasia, angiogenesis, osteoclastogenesis, and exogenous cytokine release by impeding the RANKL/RANK/OPG pathway [57,58]. Moreover, diosmetin exhibits the capacity to attenuate the expression of pro-inflammatory cytokines TNF-α, IL-1β, IL-6, and IL-8 by inhibiting protein expression within the Akt/NF-κB pathway, while also fostering the apoptosis of synovial fibroblasts (MH7A) [59]. Both scutellarin and apigenin exhibit anti-inflammatory effects by modulating TNF signaling and macrophage polarization [60,61], suggesting potential therapeutic roles in rheumatoid arthritis, which will be further explored through isolated compound testing.

While our study contributes valuable insights, it comes with certain limitations. First, although the CIA model is widely used to study the inflammatory aspects of RA, it might not capture the full pathophysiology, such as joint-specific autoimmunity seen in RA patients. Complementary models, such as the K/BxN serum-transfer arthritis model, could be used to provide a more comprehensive understanding of potential therapeutic effects, enhancing the translational relevance of research findings. Second, our analysis unveils the anti-inflammatory and metabolic mechanisms of CM in RA. However, future research is needed to elucidate the interconnected linking TNF signal pathway with anti-inflammatory. Additionally, studies involving more human cell lines and efficacy comparisons with anti-TNF therapies are required to thoroughly assess the underlying molecular mechanisms. The synergistic contributions of the active compound to the therapeutic effects of CM should be further assessed through isolated compound testing in subsequent experiments. Third, an in-depth exploration of the pharmacokinetics, tolerability, and potential contraindications associated with CM will enhance our understanding of its transition from laboratory research to dietary interventions and functional foods. Given that CM has traditionally been consumed as tea and recent research indicates its water extract contains flavonoids and terpenes with therapeutic potential for RA, it is well-suited for development into various products, such as functional ready-to-drink teas, water-soluble powders, functional herbal capsules/tablets and functional herbal tea bags. Of course, all of these are based on further in-depth research.

## 5. Conclusions

In conclusion, this study underscores CM’s potential as a therapeutic agent for RA. CM exhibits the capability to downregulate the expression of FOS, NFKBIA, JUN, MAP3K7, and IL-15 within the TNF signaling pathway, attributed to active constituents such as scutellarin and apigenin. These regulatory effects enhance glycerophospholipid and amino acid metabolism, and inhibit M1 macrophage activation. In light of the increasing global burden of autoimmune diseases, our research not only contributes to the scientific understanding of CM as a functional food but also aims to provide valuable insights that could support the development of safer, more effective dietary interventions for RA management. By validating traditional claims surrounding CM through rigorous scientific investigation, this research paves the way for its potential incorporation into modern therapeutic regimens, ultimately improving patient outcomes and quality of life.

## Figures and Tables

**Table 1:** Structural information of the main active components of CM.

NO	Compounds	Formula	Accurate Mass		Rt (min)	Error (ppm)	MS/MS	Structure
			Measured	Predicted				
1	Caryopincaolide L	C_20_H_24_O_4_	329.1761	329.1747	74.72	4.25	311.1655, 293.1548, 283.1343, 243.1024, 227.1074, 151.1123, 133.1019, 119.0864	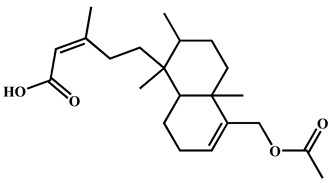
2	Scutellarein	C_15_H_10_O_6_	285.0406	285.0404	42.97	0.7	267.0303, 257.0462, 241.0501, 213.0555, 148.9880, 117.0336	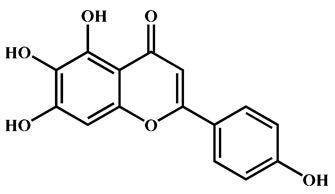
3	Rhamnetin	C_16_H_12_O_7_	315.0512	315.051	50.08	0.63	300.028	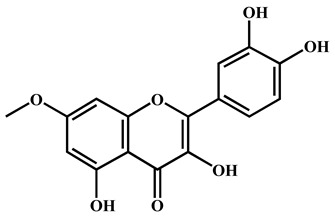
4	Apigenin	C_15_H_10_O_5_	269.0457	269.0455	61.05	0.74	241.0506, 225.0549, 151.0027, 117.0325	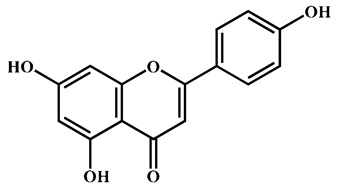
5	Diosmetin	C_16_H_12_O_6_	299.0562	299.0561	63.73	0.33	284.0328, 256.0378, 151.0000, 117.0318	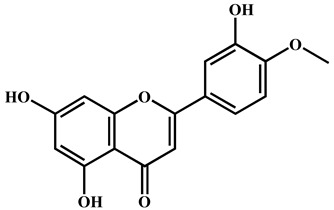

## Data Availability

The raw data supporting the conclusions of this article will be made available by the authors on request.

## Supplementary Materials

The following supporting information can be downloaded at: https://www.mdpi.com/article/10.3390/nu16244311/s1, Figure S1: Chromatogram for stability investigation of CM extract (a: signal offset display, b: signal overlap display), Figure S2: The typical pathological morphology of heart, liver, kidney H&E staining in different group, Figure S3: PCA and correlation analysis results of QC sample (A is score chart, B is one-dimensional distribution chart, C is correlation analysis chart); Figure S4: Permutation plot test of OPLS-DA model for group A vs. B (A is NC vs. CIA, B is CIA vs. CM, horizontal coordinate represents the permutation retention of the permutation test, vertical coordinate represents the value of R^2^Y or Q^2^, green dot represents the R^2^Y value obtained by the permutation test, blue square dot represents the Q^2^ value obtained by the permutation test, and the two dashed lines represent the regression lines of R^2^Y and Q^2^, respectively); Table S1: Characterization of compounds of CM by UHPLC-Q-Exactive MS/MS; Table S2: Amplified primer sequence; Table S3: Information of putative gene targets related to these 77 phytochemicals of CM; Table S4: Information of unique RA-related gene targets; Table S5: 244 common targets between CM-related targets and RA-related predicted targets; Table S6: Information of 54 core candidate nodes; Table S7: Enriched terms of 54 core candidate targets in KEGG pathways, biological processes, cellular components, and molecular functions; Table S8: Kernel therapeutic targets screened by MCODE algorithm; Table S9: Enriched terms of different cluster genes in KEGG pathways, biological processes, cellular components, and molecular functions; Table S10: Response stability in QC sample; Table S11: Summary of PLS-DA model parameters for evaluating model quality by 200 permutation tests of corresponding validation plots; Table S12: Identification of potential differential metabolites in different group.
