# Supplementary material for: From Tea to Functional Foods: Exploring Caryopteris mongolica Bunge for Anti-Rheumatoid Arthritis and Unraveling Its Potential Mechanisms
Source: Nutrients. 2024 Dec 13;16(24):4311. doi: 10.3390/nu16244311 (PMC11680032; doi:10.3390/nu16244311)
Supplement: Supplementary file 1 [file nutrients-16-04311-s001.zip › Supporting Figure Information.pdf]

S1.1 Supplementary Figures

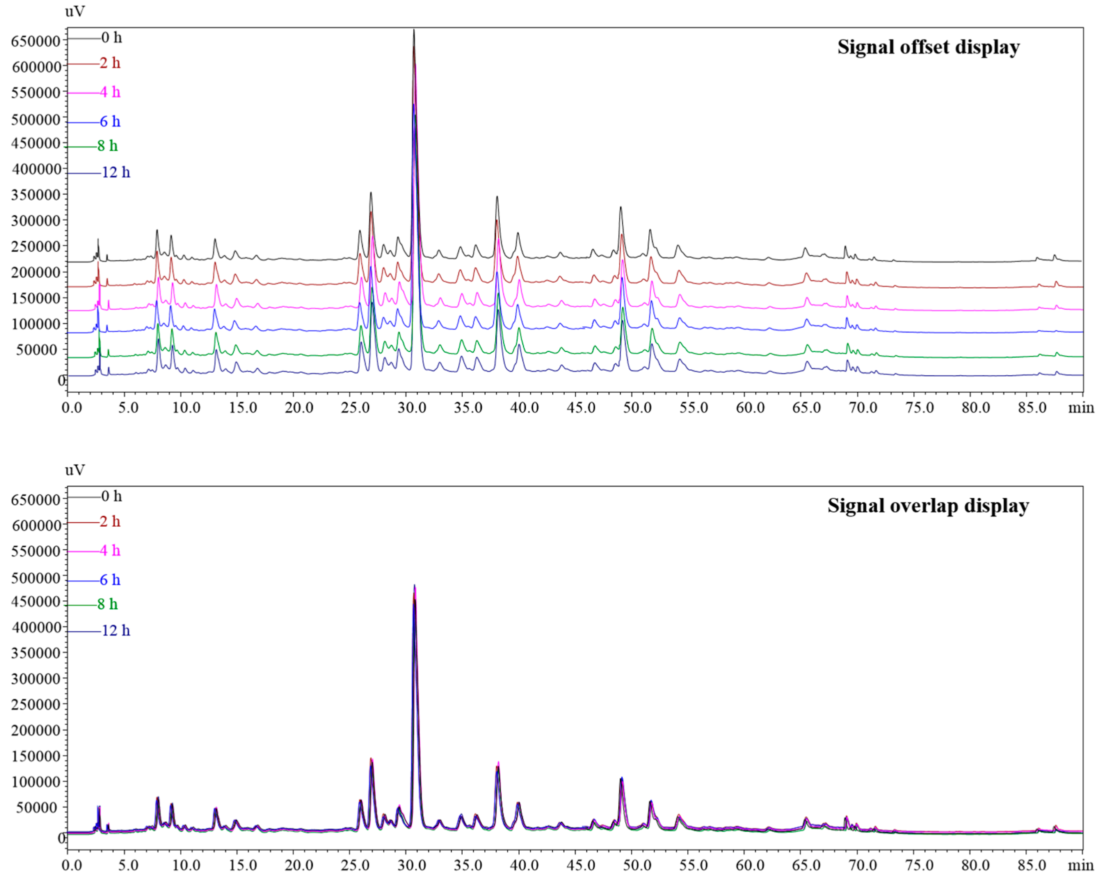

Figure S1 Chromatogram for stability investigation of CM extract(a:signal offset display, b: signal overlap display)

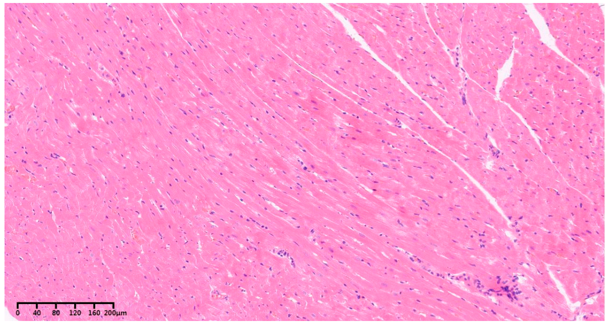

Heart H&E staining of NC rat

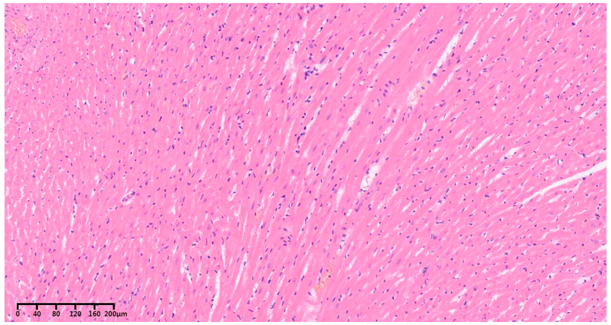

Heart H&E staining of CIA rat

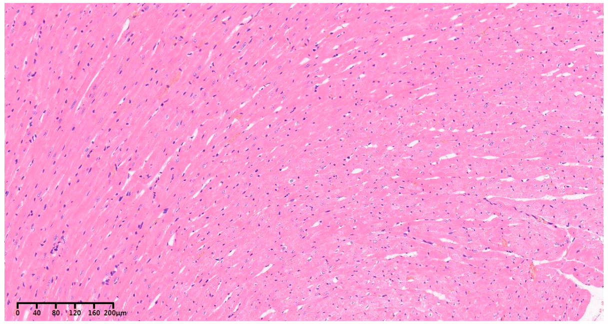

Heart H&E staining of CM-H rat

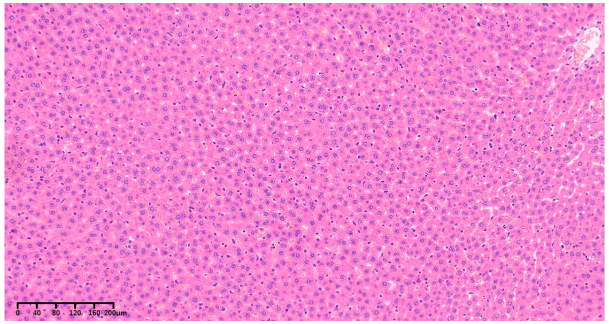

Liver H&E staining of NC rat

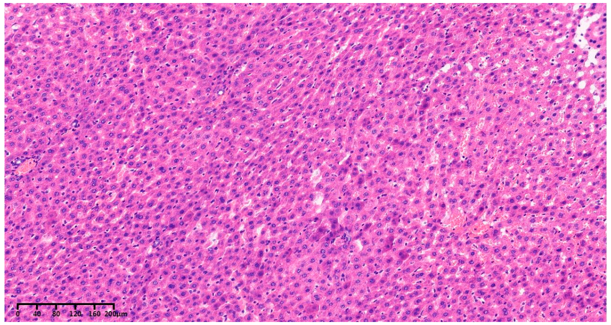

Liver H&E staining of CIA rat

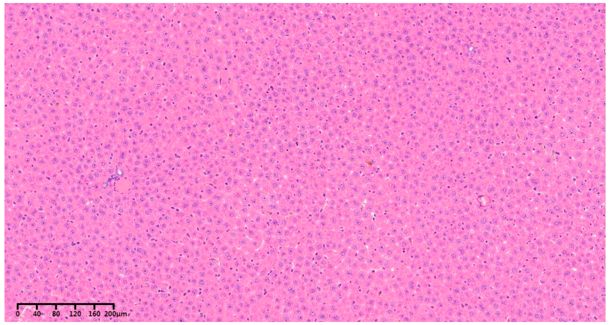

Liver H&E staining of CM-H rat

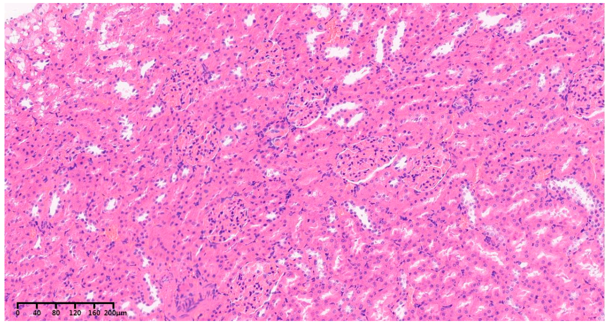

Kidney H&E staining of NC rat

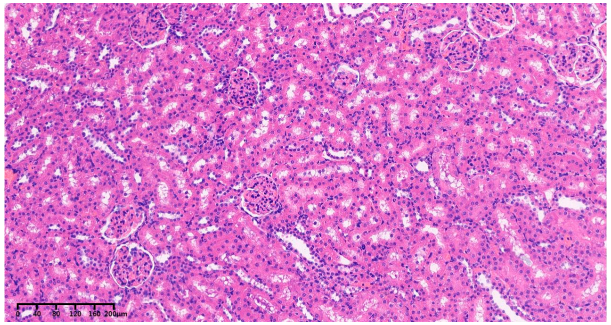

Kidney H&E staining of CIA rat

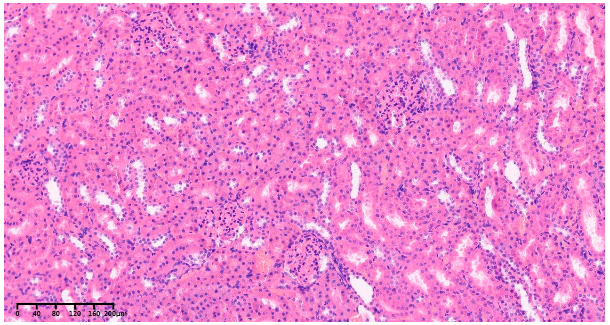

Kidney H&E staining of CM-H rat

Figure S2 The typical pathological morphology of heart, liver, kidney H&E staining in different group

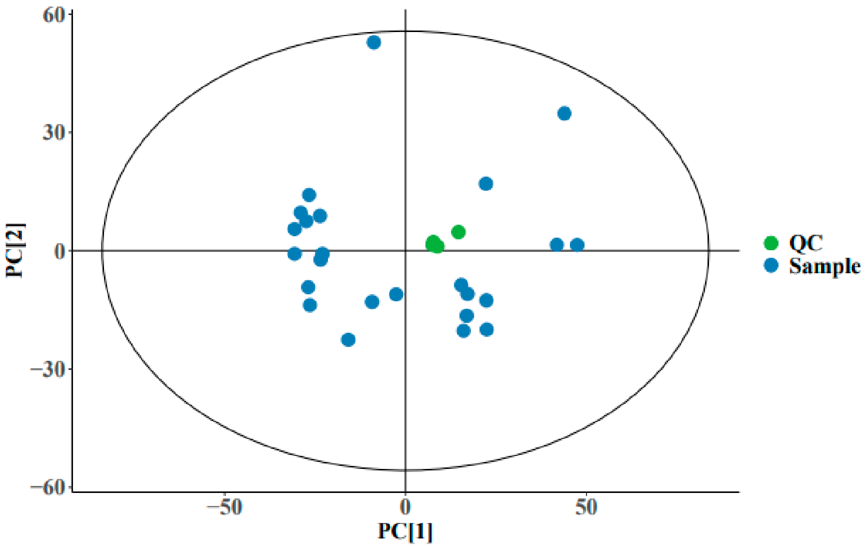

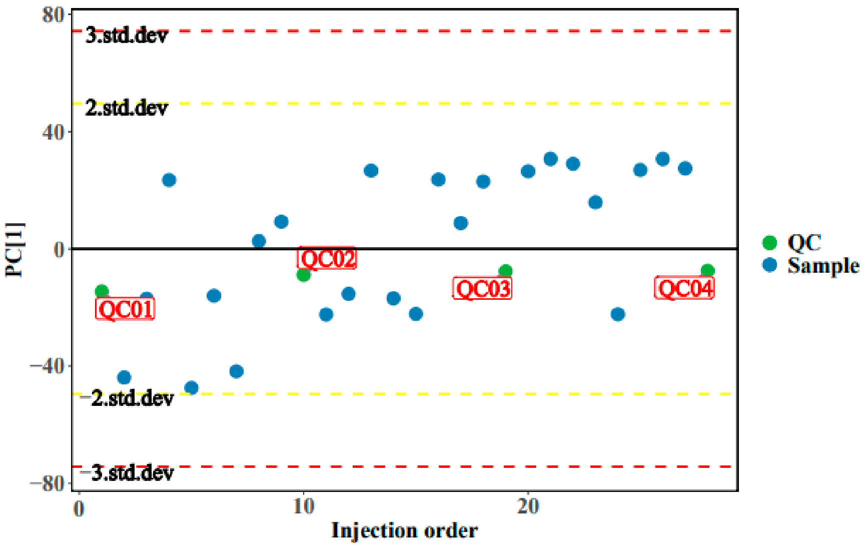

(A) (B)

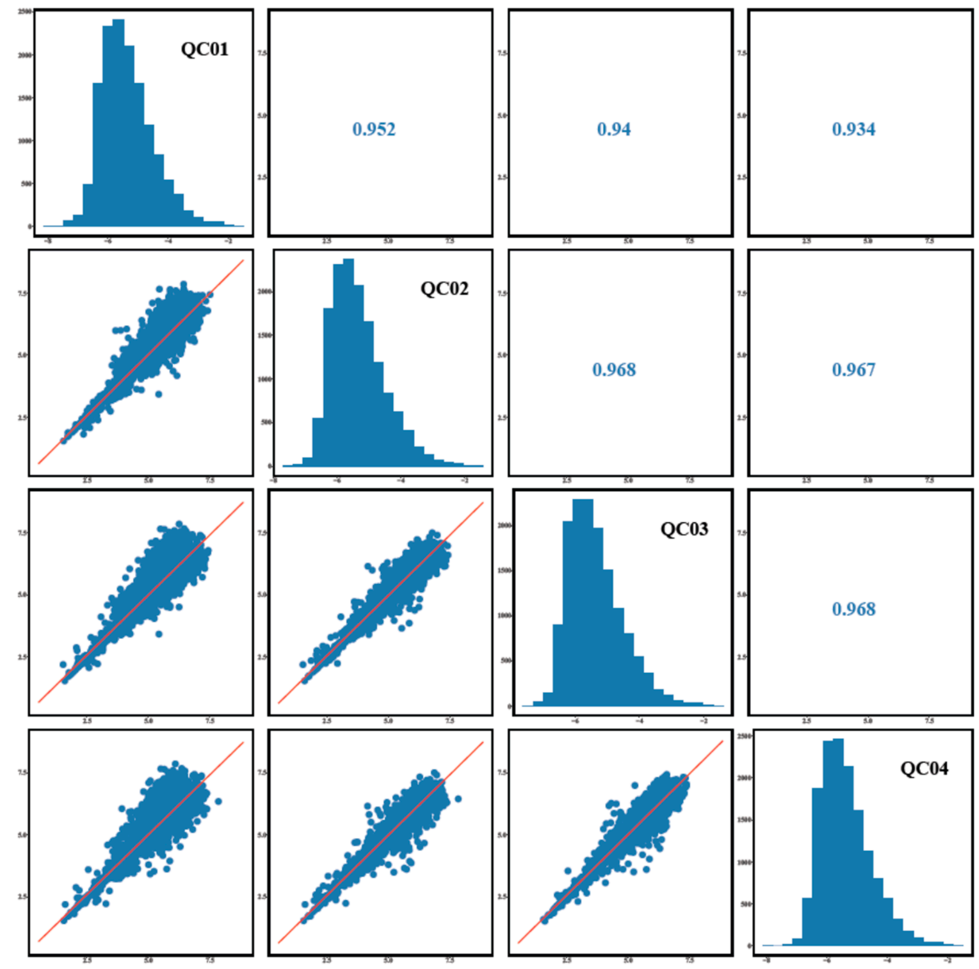

(C)

Figure S3 PCA and correlation analysis results of QC sample (A is score chart, B is one-dimensional distribution chart, C is correlation analysis chart)

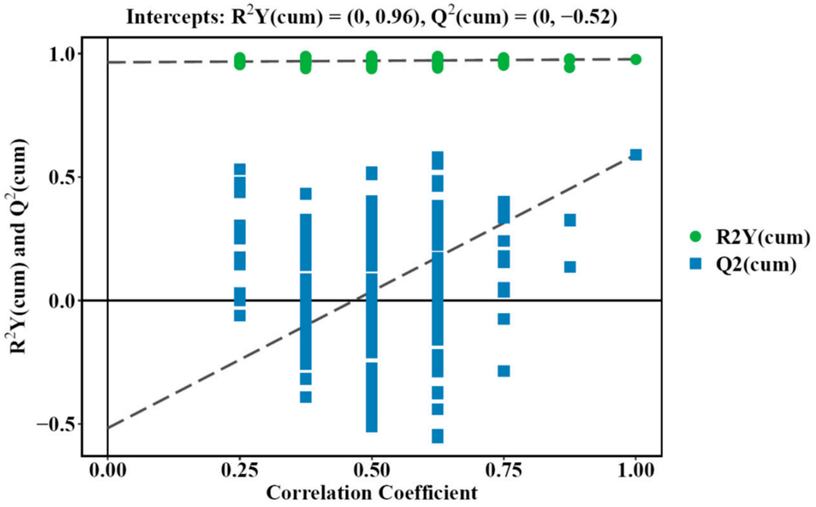

(A)

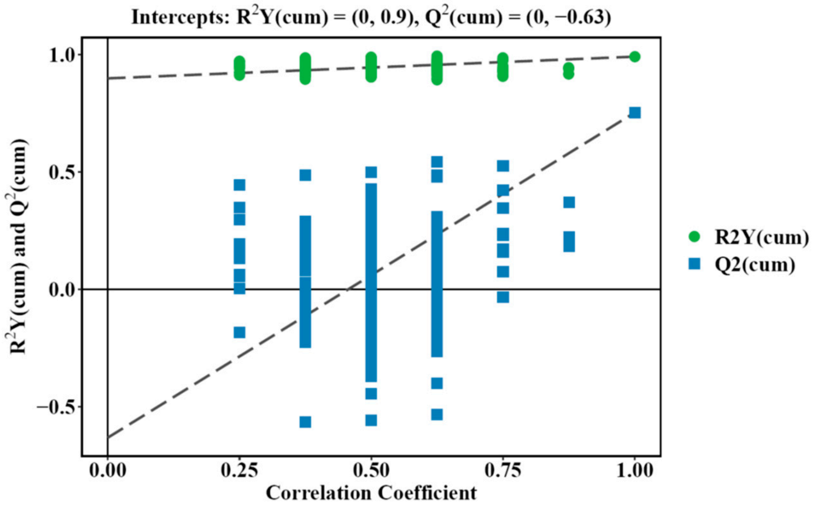

(B)

Figure S4 Permutation plot test of OPLS-DA model for group A vs B (A is NC vs CIA, B is CIA vs CM, horizontal coordinate represents the permutation retention of the permutation test, vertical coordinate represents the value of R^2Y or Q^2, green dot represents the R^2Y value obtained by the permutation test, blue square dot represents the Q^2 value obtained by the permutation test, and the two dashed lines represent the regression lines of R^2Y and Q^2, respectively)
