# Supplementary material for: From Tea to Functional Foods: Exploring Caryopteris mongolica Bunge for Anti-Rheumatoid Arthritis and Unraveling Its Potential Mechanisms
Source: Nutrients. 2024 Dec 13;16(24):4311. doi: 10.3390/nu16244311 (PMC11680032; doi:10.3390/nu16244311)
Supplement: Supplementary file 1 [file nutrients-16-04311-s001.zip › Supporting Table Information.pdf]

Table S1 Characterization of compounds of CM by UHPLC-Q-Exactive MS/MS

NO	Compounds	Formula	Accurate Mass		Rt (min)	Error（ppm）	MS/MS
			Measured	Predicted			
1	Salsolinol	$C_{10}H_{13}NO_2$	180.1013	180.1019	2.31	-3.33	158.3887, 72.0814
2	Pyrrole-2-carboxylic acid	$C_5H_5NO_2$	112.0394	112.0393	2.39	0.89	70.0657
3	Raffinose	$C_{18}H_{32}O_{16}$	527.1569	527.1582	2.51	-2.47	365.1039, 347.0939, 397.0890
4	Betaine	$C_5H_{11}NO_2$	118.0861	118.0862	2.76	-0.85	70.0656, 58.0657
5	Cellotetraose	$C_{24}H_{42}O_{21}$	701.1907	701.1902	2.85	0.71	665.2147, 485.1513, 383.1198, 341.1089, 179.0554
6	Maltopentose	$C_{30}H_{52}O_{26}$	863.2501	863.2499	2.88	0.23	827.2689, 665.2172, 545.1735, 485.1522, 341.1091, 179.0554, 161.0447
7	Ribose	$C_5H_{10}O_5$	149.0455	149.0455	2.92	0	89.0237, 103.0396, 71.0129, 131.0349
8	2,3-Dihydroxypropanoic acid	$C_3H_6O_4$	105.0188	105.0193	2.93	-4.76	75.0079
9	Pilocarpine	$C_{11}H_{16}N_2O_2$	209.1275	209.1284	2.95	-4.3	123.0916, 121.0760
10	Stachydrine	$C_7H_{13}NO_2$	144.1017	144.1019	3.08	-1.39	144.1016, 85.0593, 70.0657
11	Inositol	$C_6H_{12}O_6$	179.0553	179.0561	3.12	-4.47	161.0447, 89.0230, 101.0230
12	Quinic acid	$C_7H_{12}O_6$	191.0566	191.0561	3.17	2.62	85.0287, 173.0429, 143.0323, 127.0380
13	3-CoQA	$C_{16}H_{18}O_8$	337.0927	337.0929	3.17	-0.59	191.0554, 173.0449, 163.0391, 155.0336, 119.0502, 93.0333

14	4-CQA-Glc	$C_{22}H_{28}O_{14}$	515.1404	515.1406	3.2	-0.39	341.0884, 191.0555, 173.0441, 161.0235
15	Trigonelline	$C_7H_7NO_2$	138.0549	138.0549	3.21	0	124.0393, 110.0607, 94.0659
16	3,5-di-tert-Butyl-4-hydroxybenzaldehyde	$C_{15}H_{22}O_2$	235.1691	235.1692	3.22	-3.78	148.0752, 118.0863, 87.0444
17	Citramalic acid	$C_5H_8O_5$	147.03	147.0299	3.47	0.68	129.0191, 87.0081, 85.0289, 101.0238
18	Citric acid	$C_6H_8O_7$	191.0203	191.0197	3.72	3.14	173.0092, 129.0189, 111.0083, 87.0087
19	3-PQA	$C_{16}H_{20}O_{10}$	371.0982	371.0984	3.83	-0.54	197.0453, 191.0559, 179.0342, 173.0449, 161.0235, 135.0436
20	Gallic acid*	$C_7H_6O_5$	169.0146	169.0142	4.36	2.37	125.0220, 97.0273
21	6-O-Galloyl-glucose	$C_{13}H_{16}O_{10}$	331.0674	331.0671	4.49	0.91	169.0494, 125.0231
22	Pantothenic acid	$C_9H_{17}NO_5$	218.1029	218.1033	4.88	-1.83	146.0819, 88.0397
23	Indole-3-carboxaldehyde	C_9H_7NO	146.06	146.06	4.93	0	146.0597, 85.0593, 118.0654
24	Mesaconic acid	$C_5H_6O_4$	129.019	129.0193	5.23	-2.33	85.0287
25	Pyroquilon	$C_{11}H_{11}NO$	174.0912	174.0913	5.52	-0.57	159.0676, 131.0734
26	Clandonoside II	$C_{17}H_{26}O_{12}$	421.1351	421.1351	5.53	0	375.1296, 213.0765, 195.0662, 179.0557, 169.0860, 151.0754
27	Loganicacid	$C_{16}H_{24}O_{10}$	375.1294	375.1296	5.53	-0.53	213.0764, 195.0663, 179.0861, 151.0751, 125.0597
28	Vanillic acid*	$C_8H_8O_4$	167.035	167.0349	5.99	0.6	123.0447, 109.0289

29	3-CQA	$C_{16}H_{18}O_9$	353.0876	353.0878	6.03	-0.57	191.0555, 179.0345, 173.0450, 161.0232, 135.0443
30	Methyl Salicylate	$C_8H_8O_3$	151.0399	151.04	7.16	-0.66	109.0290, 123.0448
31	cis-1-CQA-Glc	$C_{22}H_{28}O_{14}$	515.1403	515.1406	7.38	-0.58	353.1092, 281.0674, 251.0564, 173.0448, 161.0232
32	cis-4-O-caffeoyl-glucoside	$C_{15}H_{18}O_9$	341.0876	341.0878	7.39	-0.59	281.0668, 251.0558, 221.0451, 179.0342, 161.0233, 135.0440
33	5-Methoxyindole	C_9H_9NO	148.08	148.08	7.86	2.7	133.0527, 105.0576
34	Gentisic acid	$C_7H_6O_4$	153.0193	153.0193	7.98	0	109.029
35	cis-5-CQA	$C_{16}H_{18}O_9$	353.0876	353.0878	8	-0.57	191.0553, 179.0341, 173.0439, 161.0235, 135.0440
36	4-PQA	$C_{16}H_{20}O_{10}$	371.0982	371.0984	8.03	-0.54	197.0453, 191.0559, 179.0342, 173.0449, 161.0235, 135.0436
37	3-Methyl-2-furoic acid	$C_6H_6O_3$	127.0392	127.0389	8.24	2.36	85.0594
38	Caprolactam	$C_6H_{11}NO$	114.0918	114.0913	8.29	4.38	72.0815
39	trans-4-O-caffeoyl-glucoside	$C_{15}H_{18}O_9$	341.0876	341.0878	8.42	-0.59	281.0668, 251.0558, 221.0451, 179.0342, 161.0233, 135.0440
40	trans-5-CQA	$C_{16}H_{18}O_9$	353.0876	353.0878	8.9	-0.57	191.0553, 179.0341, 173.0439, 161.0235, 135.0440
41	trans-1-CQA-Glc	$C_{22}H_{28}O_{14}$	515.1403	515.1406	9	-0.58	353.1092, 281.0674, 251.0564, 173.0448, 161.0232
42	2,5-Dihydroxybenzaldehyde	$C_7H_6O_3$	137.0242	137.0244	9	-1.46	109.0290, 81.0337, 93.0339

43	Isopropylmalic acid	$C_7H_{12}O_5$	175.0603	175.0612	9.26	-5.14	157.0495, 131.0700, 115.0387, 113.0595, 85.0644
44	Acetovanillone	$C_9H_{10}O_3$	165.0556	165.0557	9.3	-0.85	147.0440, 129.0335
45	4-CQA	$C_{16}H_{18}O_9$	353.0876	353.0878	9.53	-0.57	191.0555, 179.0342, 173.0446, 161.0231, 135.0441
46	Atropine	$C_{17}H_{23}NO_3$	290.18	290.18	10.28	0.69	133.0523, 105.0576, 87.0548
47	Esculetin	$C_9H_6O_4$	177.0185	177.0193	10.97	-4.52	149.0236, 133.0284, 105.0335, 89.0231
48	8-O-Acetylharpagide	$C_{17}H_{26}O_{11}$	405.1398	405.1402	11.4	-0.99	183.0657, 165.0547, 139.0390, 123.0443, 119.0338
49	Caffeic acid	$C_9H_8O_4$	179.0342	179.0349	11.86	-3.91	135.044
50	Andrographidoid E	$C_9H_{12}O_4$	183.0657	183.0662	12.05	-2.73	165.0550, 139.0393, 121.0284, 117.0544, 112.9843
51	6, 8-Bis-(C-glucosyl)- 7, 4′, 5′-trihydroxyflavone	$C_{27}H_{30}O_{15}$	593.1517	593.1529	12.98	-2.02	575.1383, 515.1213, 503.1201, 473.1091, 455.0974, 413.0878, 383.0775, 353.0673
52	FQA+Glc or isomer	$C_{16}H_{20}O_9$	355.1029	355.1035	13.08	-1.69	295.0795, 265.0694, 235.0586, 193.0481, 175.0374
53	Resacetophenone	$C_8H_8O_3$	151.0399	151.04	13.67	-0.66	109.029
54	5-CoQA	$C_{16}H_{18}O_8$	337.0927	337.0929	13.71	-0.59	191.0555, 173.0446, 163.0390, 155.0336, 119.0502, 93.0333
55	FQA+Glc or isomer	$C_{16}H_{20}O_9$	355.1029	355.1035	14.47	-1.69	295.0795, 265.0694, 235.0586, 193.0481, 175.0374
56	3-Hydroxycinnamic acid*	$C_9H_8O_3$	163.0395	163.04	16.23	-3.07	163.0374, 117.0533, 135.0428

57	4-Hydroxybenzaldehyde	$C_7H_6O_2$	121.0291	121.0295	17.19	-3.3	90.109
58	Campneoside II or isomer	$C_{29}H_{36}O_{16}$	639.1932	639.193	17.24	0.31	621.1834, 459.1506, 179.0342, 161.0234
59	Campneoside II or isomer	$C_{29}H_{36}O_{16}$	639.1932	639.193	17.88	0.31	621.1834, 459.1506, 179.0342, 161.0234
60	1-CoQA	$C_{16}H_{18}O_8$	337.0927	337.0929	18.19	-0.59	191.0554, 173.0449, 163.0391, , 155.0336, 119.0502, 93.0333
61	Incanide B-xyl	$C_{21}H_{30}O_{13}$	489.1618	489.1614	19.97	0.82	445.1699, 357.1183, 195.0657, 177.0553, 151.0753, 107.0489
62	4-Coumaric acid	$C_9H_8O_3$	163.0401	163.04	20.46	0.61	96.969
63	Darendoside B	$C_{21}H_{32}O_{12}$	475.1818	475.182	21.93	-0.42	429.1760, 205.0711, 163.0600, 143.0339, 119.0337
64	Ferulic acid*	$C_{10}H_{10}O_4$	193.0501	193.0506	22.08	-2.59	178.0248, 134.0351, 149.0596, 117.0532
65	Vitexin	$C_{21}H_{20}O_{10}$	431.0977	431.0984	22.89	-1.62	341.0658, 311.0565, 283.0603, 268.0362
66	Protocatechuic acid*	$C_7H_6O_4$	153.0192	153.0193	23.87	-0.65	109.0290, 135.0087, 96.9690
67	Isovitexin	$C_{21}H_{20}O_{10}$	431.0983	431.0984	23.93	-0.23	341.0681, 311.0535, 283.0573, 257.0858
68	Quercetin-3-O-(2-glucosyl)-Rha	$C_{27}H_{30}O_{16}$	609.1453	609.1461	24.37	-1.31	455.0738, 301.0323, 463.0842
69	Quercetin-3-O-glc*	$C_{21}H_{20}O_{12}$	463.0888	463.0882	24.42	1.3	301.0354, 133.0293
70	Aureoglandulosin B or C or isomer	$C_{19}H_{18}O_4$	311.1281	311.1277	24.59	1.29	289.0359, 279.1032, 251.1073, 163.0395, 137.0604, 121.1019
71	Quercetin-5-O-glu	$C_{21}H_{18}O_{13}$	477.0656	477.0675	24.77	-3.98	301.0354

72	Aureoglandulosin A or isomer	$C_{26}H_{30}O_{10}$	503.1925	503.1911	24.81	2.78	311.1285, 279.1023, 251.1073, 175.0759, 137.0603
73	Isoacteoside	$C_{29}H_{36}O_{15}$	623.1986	623.1976	24.92	1.6	161.0233, 461.1671, 315.1092, 579.1354
74	Magnoloside B or F or isomer	$C_{35}H_{46}O_{20}$	785.2519	785.2504	26.11	1.91	623.2186, 461.1671, 315.1087, 179.0342, 161.0233, 135.0440
75	PG+Caff+Rha+Ara	$C_{34}H_{44}O_{19}$	755.2412	755.2399	26.76	1.72	161.0233, 461.1669, 315.1092, 593.2111, 623.1989
76	Kaempferol-7-O-glc	$C_{21}H_{20}O_{11}$	447.0934	447.0941	26.78	-1.57	285.0380, 284.0303, 161.0436, 149.0466
77	Magnoloside B or F or isomer	$C_{35}H_{46}O_{20}$	785.2513	785.2504	26.79	1.15	623.2186, 461.1671, 315.1087, 179.0342, 161.0233, 135.0440
78	Luteolin-7-O-glu	$C_{21}H_{18}O_{12}$	461.0721	461.0726	27.3	-1.08	327.0515, 285.0379, 151.0035, 133.0290
79	Luteolin-7-O-rutinoside	$C_{27}H_{30}O_{15}$	593.1519	593.1529	27.7	-1.69	447.0938, 285.0405
80	Salicylic acid*	$C_7H_6O_3$	137.0242	137.0244	30.06	-1.46	93.034
81	Kaempferol-3-O-glucopyranosyl-7-O-rha	$C_{27}H_{30}O_{15}$	593.1519	593.1529	30.37	-1.69	429.0829, 285.0405
82	Magnoloside B or F or isomer	$C_{35}H_{46}O_{20}$	785.2523	785.2504	31.17	2.42	623.2186, 461.1671, 315.1087, 179.0342, 161.0233, 135.0440
83	1, 5-di-CQA	$C_{25}H_{24}O_{12}$	515.1191	515.1195	31.48	-0.78	353.0883, 191.0555, 179.0343, 161.0236, 135.0442
84	Aureoglandulosin B or C or isomer	$C_{19}H_{18}O_4$	311.1289	311.1277	31.77	3.86	289.0359, 279.1032, 251.1073, 163.0395, 137.0604, 121.1019
85	Magnoloside A or D	$C_{29}H_{36}O_{15}$	623.1976	623.1976	32.02	0	161.0233, 461.1671, 315.1092, 579.1354

86	Luteolin-4'-O-glc	$C_{21}H_{20}O_{11}$	447.0939	447.0941	32.06	-0.45	327.0524, 285.038, 257.0428, 229.0517
87	Umbelliferone	$C_9H_6O_3$	163.039	163.0389	32.5	0.61	145.0283, 135.0443, 107.0496, 123.0406
88	2'-O-(E)-p-coumaroyl asystasioside A	$C_{31}H_{40}O_{16}$	667.2244	667.2243	32.59	0.15	621.1819, 483.0545, 298.1606, 179.0342, 161.0233, 151.0389, 113.0232
89	Kaempferol-3-O-glu	$C_{21}H_{18}O_{12}$	461.0721	461.0726	32.62	-1.08	285.0402, 239.0355
90	Azelaic acid	$C_9H_{16}O_4$	187.0969	187.0976	33.76	-3.74	169.0867, 143.1064, 125.0960, 117.0543
91	apigenin 7-O-β-D-glc	$C_{21}H_{20}O_{10}$	431.0986	431.0984	34.04	0.46	301.0333, 269.0426, 268.0355, 257.0854, 151.0018, 117.0331
92	6'-O-vanilloyl-8- O-acetylharpagide	$C_{25}H_{32}O_{14}$	579.1718	579.169	34.07	4.83	287.0565, 271.0612, 177.0551, 137.0608
93	Kankanoside G	$C_{29}H_{36}O_{14}$	607.2048	607.2032	34.11	2.64	445.1708, 179.0346, 161.0233
94	Aureoglandulosin A or isomer	$C_{26}H_{30}O_{10}$	503.192	503.1911	34.11	1.79	311.1285, 279.1023, 251.1073, 175.0759, 137.0603
95	Hypolaetin	$C_{15}H_{10}O_7$	301.0348	301.0353	34.17	-1.66	283.0221, 255.0272
96	Incanoside C	$C_{36}H_{48}O_{20}$	799.2691	799.2666	34.73	3.13	623.2198, 461.1675, 315.1076, 193.0500, 175.0392, 113.0231, 161.2332, 135.0440
97	Isorhoifolin	$C_{27}H_{30}O_{14}$	577.1572	577.1581	34.81	-1.56	429.0829, 285.0406, 269.0457
98	4-Methylumbelliferyl-α-D-mannoside	$C_{16}H_{18}O_8$	339.1084	339.1074	34.83	2.95	177.0548, 145.0284, 117.0344
99	PG+Coum+Rha	$C_{29}H_{36}O_{14}$	607.2028	607.2032	35.01	-0.66	461.1669, 315.1089, 145.0284

100	PG+Feru+Rha+Ara	$C_{35}H_{46}O_{19}$	769.2577	769.256	35.03	2.21	593.2092, 461.1669, 193.0499, 161.0234, 175.0391
101	Galangin-7-O-glu	$C_{21}H_{18}O_{11}$	445.0774	445.0776	35.5	-0.45	269.0457, 175.0239, 157.0135, 129.0181, 117.0, 343, 113.0231
102	Isorhamnetin-7-O-glu	$C_{22}H_{20}O_{13}$	491.0828	491.0831	35.64	-0.61	315.0513, 300.0477, 175.0240
103	PG+Feru+Rha	$C_{30}H_{38}O_{15}$	637.2154	637.2132	36.35	3.45	461.1669, 315.1088, 193.0499, 175.0391, 161.0233, 113.0231
104	4-Methylumbelliferyl-β-D-mannoside	$C_{16}H_{18}O_{8}$	339.1085	339.1074	36.44	3.24	177.0554, 145.0291, 117.0397
105	Incanides A	$C_{30}H_{36}O_{16}$	651.1947	651.193	36.68	2.61	489.1618, 471.1543, 445.1718, 195.0656, 179.0342, 161.0233, 151.0753, 135.0440
106	Hydroxygenkwanin-5-O-glu	$C_{22}H_{20}O_{12}$	475.0877	475.0882	37.41	-1.05	299.0561, 284.0330, 167.0340, 113.0230
107	3, 4-di-CQA	$C_{25}H_{24}O_{12}$	515.1185	515.1195	37.85	-1.94	353.0882, 191.0557, 179.0343, 173.0445, 161.0233, 137.0237, 135.0440
108	5, 7-Dihydroxy-2-(4-hydroxyphenyl)-6, 8-bis[3, 4, 5-trihydroxy-6-(hydroxymethyl)tetrahydro-2H-pyran-2-yl]-4H-chromen-4-one	$C_{27}H_{30}O_{15}$	593.15	593.1529	37.97	-4.89	417.1042, 399.0934, 285.0384, 152.0103
109	Leucosceptoside A	$C_{30}H_{38}O_{15}$	637.2154	637.2132	38.9	3.45	161.0233
110	Diosmetin-7-O-glu	$C_{22}H_{20}O_{12}$	475.0876	475.0882	38.92	-1.26	299.0563, 284.0328, 175.0240, 151.0028, 113.0231
111	Scutellarein-7-O-glc	$C_{21}H_{20}O_{11}$	447.0946	447.0941	39.12	1.12	285.0407, 167.0038

112	8-O-Acetylharpagide	$C_{17}H_{26}O_{11}$	429.1347	429.1367	39.63	-4.66	369.1097, 351.0988, 203.0486
113	Magnoloside N	$C_{41}H_{56}O_{25}$	947.3019	947.3032	41.13	-1.37	161.0233, 785.2518, 623.2197, 461.1668, 315.1089
114	PG+Feru+Rha+Ara	$C_{35}H_{46}O_{19}$	769.2577	769.256	41.19	2.21	593.2092, 461.1669, 193.0499, 161.0234, 175.0391
115	6'-O-sinapoyl-8-O-acetylharpagid	$C_{28}H_{36}O_{15}$	635.1974	635.1951	41.4	3.62	373.0826, 245.0813, 177.0553, 167.0345, 147.0445
116	PG+Feru+Rha	$C_{30}H_{38}O_{15}$	637.2159	637.2132	41.85	4.24	461.1669, 315.1088, 193.0499, 175.0391, 161.0233, 113.0231
117	Aureoglandulosin B or C or isomer	$C_{19}H_{18}O_{4}$	311.1284	311.1277	41.92	2.25	289.0367, 279.1016, 251.1076, 163.0394, 137.0603
118	Luteolin-7-O-glc	$C_{21}H_{20}O_{11}$	447.0946	447.0941	42.09	1.12	285.0387, 179.0036, 151.0082
119	3, 5-di-CQA	$C_{25}H_{24}O_{12}$	515.1191	515.1195	42.71	-0.78	353.0883, 191.0555, 179.0343, 161.0236, 135.0442
120	Scutellarein*	$C_{15}H_{10}O_{6}$	285.0406	285.0404	42.97	0.7	267.0303, 257.0462, 241.0501, 213.0555, 148.9880, 117.0336
121	8-O-cafeoyl massenoside	$C_{26}H_{32}O_{13}$	551.1763	551.177	43.7	-1.27	491.1559, 307.0826, 265.0718, 235.0609, 205.0500, 163.0389, 145.0283
122	8-O-trans-cinnamoyl mussaenoside	$C_{26}H_{32}O_{11}$	519.1862	519.1872	44.08	-1.93	483.0919, 398.1353, 323.0774, 243.0269, 201.0164, 179.0339, 161.0232, 137.0232
123	4, 5-di-CQA	$C_{25}H_{24}O_{12}$	515.1188	515.1195	44.44	-1.36	353.0882, 341.0878, 191.0557, 179.0343, 173.0445, 161.0233, 137.0237, 135.0440

124	Incanoside D	$C_{37}H_{50}O_{20}$	813.2822	813.2822	44.77	0	637.2349, 475.1825, 398.1385, 193.0500, 175.0391
125	4-Methylumbelliferyl-α-D-glucopyranoside	$C_{16}H_{18}O_{8}$	339.1086	339.1074	45.03	3.54	177.0553, 145.0289, 117.0340
126	Isorhamnetin*	$C_{16}H_{12}O_{7}$	315.0512	315.051	45.62	0.63	300.0275, 271.0233
127	6'-O-feruloyl-8-O-acetylharpagid	$C_{27}H_{34}O_{14}$	605.1855	605.1846	45.66	1.49	499.1581, 379.1010, 361.0900, 287.0563, 177.0553, 149.0605, 121.0655
128	4-Methylumbelliferone	$C_{10}H_{8}O_{3}$	177.0554	177.0546	47.26	4.52	149.0604, 145.0291, 117.0342, 107.0492
129	Martynoside	$C_{31}H_{40}O_{15}$	651.2283	651.2294	47.43	-1.69	519.9954, 475.1826, 447.0947, 398.1534, 353.0861, 193.0499, 175.0391, 160.0154
130	PG+Feru+2Glc+Rha	$C_{42}H_{58}O_{25}$	961.2932	961.2919	49.3	1.35	785.2517, 623.2194, 461.1668, 175.0391, 161.0233, 135.0441
131	Campylopusaurone	$C_{30}H_{20}O_{12}$	571.0876	571.0881	49.89	-0.88	483.0328, 285.0406, 133.0291
132	Luteolin*	$C_{15}H_{10}O_{6}$	285.0407	285.0404	50.01	1.05	241.0493, 151.0082, 133.0274
133	Rhamnetin	$C_{16}H_{12}O_{7}$	315.0512	315.051	50.08	0.63	300.028
134	4-Methylumbelliferyl-β-D-glucopyranoside	$C_{16}H_{18}O_{8}$	339.1086	339.1074	50.12	3.54	177.0548, 145.0284, 117.0344
135	Isomartynoside	$C_{31}H_{40}O_{15}$	651.2304	651.2294	52.54	1.54	505.1717, 475.1810, 399.0930, 193.0499, 175.0392
136	PG+Coum+Rha	$C_{29}H_{36}O_{14}$	607.2026	607.2032	52.89	-0.99	461.1669, 315.1089, 145.0284
137	Tyrosol + Caff+ 2Rha	$C_{29}H_{36}O_{13}$	591.2073	591.2078	54.14	-0.85	161.0233

138	Aureoglandulosin A	$C_{20}H_{18}O_5$	339.1216	339.1227	54.29	-3.24	321.1131, 307.0974, 177.0555, 161.0605, 145.0289
139	Lonicerin	$C_{27}H_{30}O_{15}$	593.1519	593.1529	54.44	-1.69	285.0405
140	Diosmetin-5-O-glu	$C_{22}H_{20}O_{12}$	475.0878	475.0882	54.97	-0.84	299.0563, 284.0328, 175.0240, 151.0031, 113.0231
141	Incanide B	$C_{31}H_{46}O_{15}$	657.2745	657.2764	56.65	-2.89	489.1619, 475.1511, 357.1205, 195.0656, 177.0549, 151.0753, 107.0490
142	Rutin*	$C_{27}H_{30}O_{16}$	609.1453	609.1461	57.5	-1.31	301.0355
143	Apigenin*	$C_{15}H_{10}O_5$	269.0457	269.0455	61.05	0.74	241.0506, 225.0549, 151.0027, 117.0325
144	2'-O-(E)-p-coumaroyl caryocanoside B	$C_{42}H_{54}O_{22}$	909.3055	909.3033	61.24	2.42	805.2565, 783.2729, 673.2123, 607.2228, 509.1442, 265.0725, 193.0498, 175.0391
145	Dillenetin	$C_{17}H_{14}O_7$	329.0665	329.0667	62.79	-0.61	314.0435, 299.0194, 285.0407, 271.0251, 161.0250, 117.0345
146	Diosmetin*	$C_{16}H_{12}O_6$	299.0562	299.0561	63.73	0.33	284.0328, 256.0378, 151.0000, 117.0318
147	Kolavonic acid or isomer	$C_{18}H_{28}O_3$	293.2118	293.2111	66.83	2.39	275.2017, 257.1909, 239.1799, 173.1332, 147.1173, 133.1020, 119.0863
148	Andrographolide	$C_{20}H_{30}O_5$	351.2152	351.2166	68.87	-3.99	333.2063
149	Quercetin-5-O-glc	$C_{21}H_{20}O_{12}$	463.0888	463.0882	71.06	1.3	283.0251, 255.0298, 117.0340
150	Caryopterisoid P	$C_{18}H_{26}O_3$	291.1966	291.1955	71.08	3.78	273.1862, 255.1758, 249.1503, 217.1595, 159.1175, 145.1020, 135.0812, 131.0864,

151	Rhein	$C_{15}H_8O_6$	283.0245	283.0248	71.76	-1.06	257.0824, 239.0714
152	Chrysophanol	$C_{15}H_{10}O_4$	253.0502	253.0506	72.38	-1.58	238.0365, 225.0557
153	Acacetin	$C_{16}H_{12}O_5$	283.0613	283.0611	72.59	0.71	268.0379, 133.0287
154	Aureoglandulosin F	$C_{21}H_{26}O_4$	343.1917	343.1903	73.6	4.08	325.1810, 257.1185, 135.1176, 121.1021
155	Caryopincaolide L	$C_{20}H_{24}O_4$	329.1761	329.1747	74.72	4.25	311.1655, 293.1548, 283.1343, 243.1024, 227.1074, 151.1123, 133.1019, 119.0864
156	Trachelogenin	$C_{21}H_{24}O_7$	389.1568	389.1594	75.54	-6.68	350.4656, 303.0839, 149.0227
157	Piperine	$C_{17}H_{19}NO_3$	286.1439	286.1437	76.26	0.7	201.0545, 135.0441
158	Cyrtophyllone B or isomer	$C_{20}H_{28}O_4$	333.2073	333.206	77.29	3.9	315.1967, 287.1656, 193.0869, 177.0555, 165.0553
159	Cyperotundone	$C_{15}H_{22}O$	219.174	219.1743	77.57	-1.37	173.0811, 155.0700, 82.9455
160	Microstegiol	$C_{20}H_{26}O_2$	299.2005	299.2019	77.63	-4.68	257.1548, 243.1389, 229.1232, 217.1231, 205.1230, 187.0764, 133.1017
161	Sugiol or isomer	$C_{20}H_{28}O_2$	301.2175	301.2161	77.81	4.65	283.2072, 245.1544, 231.1386, 217.1230, 205, 1231, 191.1075, 123.1172
162	14-Deoxycoleon U	$C_{20}H_{26}O_4$	331.1913	331.1903	77.95	3.02	313.1809, 295.1708, 285.1858, 257.1180, 247.0973, 177.0562, 165.0554
163	Cyrtophyllone B or isomer	$C_{20}H_{28}O_4$	333.2071	333.206	78.27	3.3	315.1967, 287.1656, 193.0869, 177.0555, 165.0553

164	Caryopterisoid O	$C_{18}H_{28}O_2$	277.2171	277.2162	78.41	3.25	259.2082, 249.1494, 149.0240, 135.1176, 121.1020, 107.0864
165	Caryopincaolide K	$C_{20}H_{24}O_3$	313.1813	313.1798	79.11	4.79	295.1709, 245.1188, 217.0870, 203.1076, 177.0554, 111.0813
166	Royleanone	$C_{20}H_{28}O_3$	315.1966	315.1967	79.29	-0.32	112.9853, 180.8996, 158.2681, 136.9097
167	Kolavonic acid or isomer	$C_{18}H_{28}O_3$	293.2122	293.2111	79.37	3.75	275.2016, 257.1914, 219.1751, 177.1287, 149.0968, 133.1019, 119.0863
168	Cyrtophyllone B or isomer	$C_{20}H_{28}O_4$	333.207	333.206	79.51	3	315.1967, 287.1656, 193.0869, 177.0555, 165.0553
169	Isorosmanol	$C_{20}H_{26}O_5$	345.171	345.1707	79.72	0.87	301.1477
170	Carnosol	$C_{20}H_{26}O_4$	331.1915	331.1903	79.72	3.62	313.1810, 261.1133, 285.1857, 271.1339, 253.1233, 245.1183
171	Rosmanol	$C_{20}H_{26}O_5$	345.171	345.1707	80.65	0.87	301.1477
172	Caryopterisoid M	$C_{22}H_{36}O_4$	365.2699	365.2683	80.85	4.38	347.2600, 305.2489, 135.1175, 121.1021, 107.0862
173	α-Pyrrolidinopropiophenone	$C_{13}H_{17}NO$	204.1382	204.1383	81.43	-0.49	204.1379, 149.0230, 164.9305
174	Caryopincaolide H	$C_{19}H_{26}O_3$	303.1969	303.1954	81.66	4.95	245.1539, 217.1235, 205.1234, 191.1083, 177.1284, 151.1128, 123.1176
175	Carnosic acid	$C_{20}H_{28}O_4$	333.2071	333.206	82.72	3.3	287.2001, 269.1900, 205.1225, 245.1536

176	α-Cyperone	$C_{15}H_{22}O$	219.1747	219.1743	83.16	1.83	173.0811, 155.0700, 82.9455
177	Caryopterisoid Q	$C_{22}H_{30}O_4$	359.222	359.2216	83.17	1.11	313.1809, 285.1497, 203.1073, 165.0552, 119.0862
178	1-Oxomicrostegiol	$C_{20}H_{24}O_3$	313.1811	313.1798	83.2	4.15	285.1499, 203.1076, 193.0869, 177.0554, 165.0553, 119.0863
179	Caryopterisoid G	$C_{22}H_{32}O_4$	361.2387	361.2373	83.52	3.88	343.2286, 315.1968, 287.1650, 193.0863, 165.0556, 135.1177, 123.1174
180	Sugiol or isomer	$C_{20}H_{28}O_2$	301.2173	301.2161	84.69	3.98	283.2072, 245.1544, 231.1386, 217.1230, 205, 1231, 191.1075, 123.1172
181	Caryopterisoid L	$C_{22}H_{34}O_4$	363.2521	363.2529	84.94	-2.2	263.2018, 233.1546, 135.1176

Table S2 Amplified primer sequence

Gene	Primer sequence (5'-3')	Length (bp)
FOS	Forward: TCCAAGCGGAGACAGATCAACT<br>Reverse: TCAAGTCCAGGGAGGTCACAGA	177
NFKBIA	Forward: TCGTGGAGCACTTGGTGACTT<br>Reverse: GTAGCCCTGGTAGGTTACTCTGTTG	161
JUN	Forward: AAACGACCTTCTACGACGATGC<br>Reverse: AGTGGTGATGTGCCCATTGCTG	254
IL15	Forward: CCATGTCTTCATTTTGGGCTGT<br>Reverse: CAGGAGAAAGCAGTTCATTGCAGTA	187
MAP3K7	Forward: AACCAGGCAAAGCAACAGAGTG<br>Reverse: GGCTTGGTATAGGCTGTAGTCG	161
GAPDH	Forward: CTGGAGAAACCTGCCAAGTATG<br>Reverse: GGTGGAAGAATGGGAGTTGCT	138

Table S3 Information of putative gene targets related to these 77 phytochemicals of CM

No.	Compound targets	No.	Compound targets	No.	Compound targets	No.	Compound targets	No.	Compound targets
1	ABAT	148	CDK5	295	GLO1	442	MAPK11	589	PSENEN
2	ABCB1	149	CDK5R1	296	GLRA1	443	MAPK12	590	PSMB5
3	ABCC1	150	CDK6	297	GMPR	444	MAPK14	591	PTAFR
4	ABCC9	151	CDK8	298	GPBAR1	445	MAPK3	592	PTGDR
5	ABCG2	152	CDK9	299	GPI	446	MAPK8	593	PTGDR2
6	ABL1	153	CES1	300	GPR18	447	MAPK9	594	PTGER1
7	ABL2	154	CES2	301	GPR35	448	MAPT	595	PTGER2
8	ACACB	155	CFB	302	GPR55	449	MB	596	PTGER3
9	ACE	156	CFD	303	GPR88	450	MC4R	597	PTGER4
10	ACE2	157	CFTR	304	GRIA2	451	MCL1	598	PTGES
11	ACHE	158	CHEK1	305	GRIN1	452	MDM2	599	PTGS1
12	ACLY	159	CHRM1	306	GRIN2A	453	MDM4	600	PTGS2
13	ACPP	160	CHRM2	307	GRK1	454	MERTK	601	PTK2
14	ADA	161	CHRM3	308	GRK6	455	MET	602	PTK2B
15	ADAM17	162	CHRM4	309	GRM1	456	METAP2	603	PTP4A3
16	ADAMTS4	163	CHRM5	310	GRM2	457	MIF	604	PTPN1
17	ADAMTS5	164	CHRNA7	311	GRM5	458	MINK1	605	PTPN11
18	ADH1A	165	CHUK	312	GSK3A	459	MKNK2	606	PTPN2
19	ADH5	166	CISD1	313	GSK3B	460	MME	607	PTPN6
20	ADK	167	CLK4	314	GSR	461	MMP1	608	PTPRS
21	ADORA1	168	CMA1	315	GSTM1	462	MMP12	609	PYGL
22	ADORA2A	169	CNR1	316	GSTP1	463	MMP13	610	PYGM
23	ADORA2B	170	CNR2	317	GUSB	464	MMP14	611	QDPR
24	ADORA3	171	COMT	318	HAO2	465	MMP16	612	RAC1
25	ADRA1A	172	CPA1	319	HCAR2	466	MMP2	613	RAP2A
26	ADRA1B	173	CPB1	320	HCK	467	MMP3	614	RARA
27	ADRA1D	174	CPT1A	321	HCRTR1	468	MMP7	615	RARB
28	ADRA2A	175	CPT1B	322	HCRTR2	469	MMP8	616	RASGRP3
29	ADRA2C	176	CRHR1	323	HDAC1	470	MMP9	617	RBP4

30	AGTR1	177	CSF1R	324	HDAC2	471	MPG	618	REN
31	AGTR2	178	CSNK1A1	325	HDAC3	472	MPI	619	RET
32	AHCY	179	CSNK1D	326	HDAC4	473	MPO	620	RHOA
33	AHR	180	CSNK2A1	327	HDAC5	474	MSR1	621	RIPK2
34	AKR1A1	181	CSNK2B	328	HDAC6	475	MTHFD1	622	RNASEH1
35	AKR1B1	182	CTSA	329	HDAC7	476	MTNR1A	623	ROCK1
36	AKR1B10	183	CTSB	330	HDAC8	477	MTNR1B	624	ROCK2
37	AKR1C1	184	CTSD	331	HGF	478	MTOR	625	RORA
38	AKR1C2	185	CTSG	332	HIF1A	479	MYLK	626	ROS1
39	AKR1C3	186	CTSK	333	HK1	480	NAE1	627	RPA1
40	AKR1C4	187	CTSL	334	HK2	481	NCOR2	628	RPS6KA5
41	AKT1	188	CTSS	335	HMGCR	482	NCSTN	629	RTN4R
42	ALB	189	CTSV	336	HMOX1	483	NEK1	630	RXRA
43	ALDH1A1	190	CXCR1	337	HNF4A	484	NEK2	631	RXRB
44	ALDH2	191	CXCR2	338	HPGD	485	NEK6	632	RXRG
45	ALDH5A1	192	CYP11B1	339	HPGDS	486	NFE2L2	633	S100A9
46	ALK	193	CYP11B2	340	HPRT1	487	NFKB1	634	S1PR3
47	ALOX12	194	CYP17A1	341	HRH1	488	NFKBIA	635	SAE1
48	ALOX15	195	CYP19A1	342	HRH2	489	NOS2	636	SCN5A
49	ALOX5	196	CYP1A2	343	HRH3	490	NOS3	637	SCN9A
50	ALOX5AP	197	CYP1B1	344	HRH4	491	NOX4	638	SEC14L2
51	ALPG	198	CYP24A1	345	HSD11B1	492	NPC1L1	639	SELE
52	ALPL	199	CYP26A1	346	HSD11B2	493	NPY2R	640	SELP
53	AMPD1	200	CYP27A1	347	HSD17B1	494	NPY5R	641	SERPINA1
54	AMPD2	201	CYP2A6	348	HSD17B10	495	NQO1	642	SERPINA6
55	AMPD3	202	CYP2C19	349	HSD17B2	496	NQO2	643	SERPINE1
56	AMY1A	203	CYP2C9	350	HSD17B3	497	NR1H2	644	SGK1
57	ANXA5	204	CYP51A1	351	HSP90AA1	498	NR1H3	645	SHBG
58	APEX1	205	DAGLA	352	HSP90AB1	499	NR1H4	646	SIGMAR1
59	APH1A	206	DAO	353	HSPA1A	500	NR1I2	647	SIRT2
60	APH1B	207	DAPK1	354	HSPA5	501	NR1I3	648	SLC10A2
61	APP	208	DDR1	355	HSPA8	502	NR3C1	649	SLC22A12
62	APRT	209	DDR2	356	HTR1A	503	NR3C2	650	SLC22A6

63	AR	210	DDX39B	357	HTR1D	504	NR4A1	651	SLC28A2
64	ARG1	211	DDX6	358	HTR2A	505	NTRK1	652	SLC29A1
65	ATG5	212	DHFR	359	HTR2B	506	NTRK3	653	SLC6A2
66	ATIC	213	DHODH	360	HTR2C	507	NTSR1	654	SLC6A3
67	ATP12A	214	DNTT	361	HTR6	508	NUAK1	655	SLC6A4
68	ATR	215	DPP4	362	HTR7	509	OGA	656	SLC6A5
69	AURKA	216	DRD1	363	ICMT	510	OPRD1	657	SLC6A9
70	AURKB	217	DRD2	364	IDH1	511	OPRK1	658	SLK
71	AURKC	218	DRD3	365	IDO1	512	OPRL1	659	SMO
72	AVPR1A	219	DRD4	366	IFNGR1	513	OPRM1	660	SMTN
73	AVPR2	220	DUSP3	367	IGF1	514	OXER1	661	SMYD2
74	AXL	221	DUT	368	IGF1R	515	OXTR	662	SNCA
75	B3GAT1	222	DYRK1A	369	IKBKB	516	P2RX3	663	SOD2
76	BACE1	223	ECE1	370	IL15	517	P2RX7	664	SORD
77	BACE2	224	EDNRA	371	IL17F	518	PABPC1	665	SPHK2
78	BCHE	225	EGFR	372	IL1B	519	PADI4	666	SQLE
79	BCL2	226	EGLN1	373	IL2	520	PANK3	667	SRC
80	BCL2A1	227	EIF2AK1	374	IMPDH2	521	PARP1	668	SRD5A1
81	BCL2L1	228	EIF2AK2	375	INSR	522	PDCD4	669	SRD5A2
82	BCL2L2	229	ELANE	376	IRAK4	523	PDE10A	670	ST6GAL1
83	BLK	230	ELAVL1	377	ISG20	524	PDE2A	671	STAT1
84	BLM	231	ENPP2	378	ITGA2B	525	PDE4B	672	STAT3
85	BMP2	232	EP300	379	ITGA4	526	PDE4D	673	STAT5A
86	BPI	233	EPHA3	380	ITGA5	527	PDE5A	674	STAT6
87	BRAF	234	EPHA6	381	ITGAL	528	PDE7A	675	STK10
88	BRD3	235	EPHA7	382	ITGAV	529	PDGFRB	676	STK26
89	BRD4	236	EPHB4	383	ITGB1	530	PER2	677	STK3
90	BRPF1	237	EPHB6	384	ITGB3	531	PFKFB3	678	STK32B
91	BRS3	238	EPHX1	385	ITGB7	532	PGF	679	STK33
92	BST1	239	EPHX2	386	JAK1	533	PGGT1B	680	STK35
93	BTK	240	ERBB2	387	JAK2	534	PGR	681	STS
94	C3	241	ERN1	388	JAK3	535	PI4KB	682	SYK
95	C5AR1	242	ESR1	389	JUN	536	PIK3CA	683	TACR1

96	CA1	243	ESR2	390	KCNA3	537	PIK3CB	684	TACR3
97	CA12	244	ESRRA	391	KCNA5	538	PIK3CD	685	TAP1
98	CA13	245	EZH2	392	KCNE1	539	PIK3CG	686	TAS2R31
99	CA14	246	F10	393	KCNH2	540	PIK3R1	687	TBXA2R
100	CA2	247	F2	394	KCNK3	541	PIM1	688	TBXAS1
101	CA3	248	F2R	395	KCNMA1	542	PIM2	689	TDP1
102	CA4	249	F2RL1	396	KCNQ1	543	PIM3	690	TEK
103	CA5A	250	F3	397	KDM1A	544	PIN1	691	TERT
104	CA5B	251	F8	398	KDM2A	545	PKN1	692	TGFB2
105	CA6	252	FAAH	399	KDM3A	546	PLA2G10	693	TGFBR1
106	CA7	253	FABP1	400	KDM4A	547	PLA2G1B	694	TGFBR2
107	CA9	254	FABP2	401	KDM4C	548	PLA2G2A	695	THRA
108	CACNA2D1	255	FABP3	402	KDM4E	549	PLA2G2E	696	TIE1
109	CACNA2D2	256	FABP4	403	KDM5C	550	PLA2G2F	697	TK1
110	CAMK2B	257	FABP5	404	KDM6B	551	PLA2G4A	698	TLR4
111	CAMK2D	258	FADS1	405	KDR	552	PLA2G5	699	TNF
112	CAPN1	259	FAP	406	KIF11	553	PLA2G7	700	TNIK
113	CAPN2	260	FBP1	407	KIT	554	PLAA	701	TNKS
114	CASP1	261	FCAR	408	KLF5	555	PLAT	702	TNKS2
115	CASP3	262	FDFT1	409	KLK5	556	PLAU	703	TNNC1
116	CASP6	263	FFAR1	410	LCK	557	PLEC	704	TNNI3
117	CASP7	264	FGF1	411	LCN2	558	PLG	705	TNNT2
118	CASR	265	FGFR1	412	LDHA	559	PLK1	706	TOP1
119	CAT	266	FGFR2	413	LDHB	560	PMS2	707	TOP2A
120	CBR1	267	FGFR3	414	LGALS2	561	PNMT	708	TPMT
121	CCKAR	268	FGFR4	415	LGALS3	562	PNP	709	TREM1
122	CCKBR	269	FGR	416	LIMK1	563	POLA1	710	TRIM24
123	CCL4	270	FKBP1A	417	LIMK2	564	POLB	711	TRPA1
124	CCL5	271	FLT1	418	LIPE	565	PPARA	712	TRPM8
125	CCNA1	272	FLT3	419	LRRK2	566	PPARD	713	TRPV1
126	CCNA2	273	FLT4	420	LTA4H	567	PPARG	714	TRPV3
127	CCNB1	274	FNTA	421	LTF	568	PPOX	715	TSPO
128	CCNB2	275	FNTB	422	LYN	569	PPP1CA	716	TTL

129	CCNB3	276	FOS	423	LYPLA1	570	PPP1CB	717	TTR
130	CCNC	277	FPR1	424	LYPLA2	571	PPP1CC	718	TUBB1
131	CCND1	278	FTO	425	MANBA	572	PPP2CA	719	TYK2
132	CCNE1	279	FUT7	426	MAOA	573	PRCP	720	TYMS
133	CCNE2	280	FYN	427	MAOB	574	PRDX5	721	TYR
134	CCR1	281	G6PD	428	MAP2K1	575	PREP	722	TYRO3
135	CCR2	282	GAA	429	MAP2K5	576	PRKAA2	723	UGT2B7
136	CCR5	283	GABBR1	430	MAP3K10	577	PRKAB1	724	UQCRB
137	CCR9	284	GABRA1	431	MAP3K11	578	PRKAG1	725	VDR
138	CD209	285	GABRA2	432	MAP3K19	579	PRKCA	726	VHL
139	CD38	286	GABRA3	433	MAP3K20	580	PRKCB	727	WAS
140	CD3E	287	GABRA5	434	MAP3K5	581	PRKCD	728	XDH
141	CDC25A	288	GABRB3	435	MAP3K7	582	PRKCG	729	XIAP
142	CDC25B	289	GABRG2	436	MAP3K8	583	PRKCH	730	YES1
143	CDC25C	290	GAPDH	437	MAP3K9	584	PRKCQ	731	ZAP70
144	CDC7	291	GART	438	MAP4K4	585	PRKCZ		
145	CDK1	292	GBA	439	MAP4K5	586	PRKDC		
146	CDK2	293	GC	440	MAPK1	587	PSEN1		
147	CDK4	294	GCGR	441	MAPK10	588	PSEN2		

Table S4 Information of unique RA-related gene targets

No.	Disease targets	No.	Disease targets	No.	Disease targets	No.	Disease targets	No.	Disease targets
1	PTPN22	464	LINC-ROR	927	HNRNPA2B1	1390	H3FJ	1853	ASRT2
2	TNF	465	CP	928	ISG20	1391	H2AFN	1854	RNR5
3	IL6	466	PVT1	929	HLA-DPA1	1392	VHX	1855	ZNF73
4	IL10	467	POMC	930	CFB	1393	NEDFACH	1856	ACF
5	HLA-DRB1	468	CYP1A2	931	CCL27	1394	IRID1	1857	CECR
6	CIITA	469	TNFSF10	932	BACH2	1395	SPI3	1858	LRE1
7	PSTPIP1	470	NFKB2	933	LST1	1396	IMD57	1859	POTEH
8	STAT4	471	BDNF-AS	934	CDSN	1397	COXPD19	1860	ZNF72
9	SLC22A4	472	CYP2C9	935	RPP14	1398	SPG77	1861	XKR3
10	IL2RA	473	IGKC	936	GSDMB	1399	DCWHKTA	1862	CECR2
11	IRF5	474	AFF3	937	C2	1400	HCC2	1863	DEL22q11.2
12	MIF	475	MECP2	938	NELFE	1401	HPS11	1864	DER22t11-22
13	MIR146A	476	CD19	939	KIAA1109	1402	FIH2	1865	DER22t8-22
14	CD244	477	JAK2	940	SYNGR1	1403	HDLCQ7	1866	DFNB40
15	NFKBIL1	478	F2	941	COL4A1	1404	MGCPH	1867	DUP22q11.2
16	HLA-B	479	SRC	942	DAXX	1405	NPHP19	1868	IGLJ@
17	IL2RB	480	MIR34A	943	ETFA	1406	KIAA0386	1869	IGLV@
18	LTA	481	SOD1	944	RTKN2	1407	H2AA	1870	SLC25A18
19	IL1B	482	DNASE1	945	HSPA1L	1408	GOUT4	1871	ATP6V1E1
20	PADI4	483	PXK	946	SMTNL2	1409	MVCD7	1872	BCL2L13
21	CRP	484	CD226	947	IL12RB2	1410	TEBIVANED1	1873	BID
22	MIR155	485	JAK1	948	FADS1	1411	RMNS	1874	MICAL3
23	H19	486	KIF5A	949	EMCN	1412	TEBIVANED3	1875	MIR648
24	COPA	487	TMX2-CTNND1	950	ATP6V1G2	1413	H3.1	1876	PEX26
25	IL17A	488	TNFRSF14	951	PSMB8	1414	RNTMT1	1877	TUBA8
26	AP4B1-AS1	489	COL1A1	952	RARB	1415	H2AFP	1878	USP18

27	TLR1	490	CCN2	953	GPSM3	1416	H4M	1879	GGTLC3
28	ZFAS1	491	OLIG3	954	CARD9	1417	TRNS	1880	RIMBP3
29	CTLA4	492	IRF4	955	TPD52	1418	H2AFE	1881	DGCR6
30	HOTAIR	493	MMP15	956	MPIG6B	1419	TEBIVANED2	1882	PRODH
31	NOD2	494	SLC17A5	957	TMEM187	1420	H2AFD	1883	DGCR5
32	MIR132	495	ADAMTS5	958	MYO18B	1421	TEBIVANED4	1884	DGCR2
33	MMP3	496	BIRC5	959	NTN1	1422	JKAP	1885	ESS2
34	TNFRSF1A	497	XIAP	960	SH3PXD2A	1423	RIEG3	1886	TSSK2
35	IL1RN	498	HLA-DQA2	961	ELMO1	1424	DFNB91	1887	GSC2
36	MALAT1	499	IRF1	962	CLLS4	1425	AIEFL	1888	SLC25A1
37	SNHG29	500	SUMO4	963	CSCI	1426	DFNB66	1889	CLTCL1
38	MIR150	501	MIR151A	964	DEL6pter	1427	PL48	1890	DVL1P1
39	NLRP1	502	MTRR	965	INTLQ3	1428	TFQTL2	1891	HIRA
40	SUPT20H	503	ENG	966	LRSL	1429	JSP1	1892	MRPL40
41	CHRNG	504	CASP8	967	DUSP22	1430	ASGD3	1893	UFD1L
42	LACC1	505	MUC1	968	EXOC2	1431	NSC	1894	CDC45L
43	PTPN2	506	PTX3	969	HUS1B	1432	DFNB104	1895	CLDN5
44	UCA1	507	NLRP12	970	FOXQ1	1433	DFNA21	1896	GP1BB
45	IL18	508	ADGRE5	971	FOXF2	1434	ERVK-6	1897	TBX1
46	TNFRSF1B	509	ADAMTS12	972	FOXCUT	1435	HYPT10	1898	GNB1L
47	CD247	510	IFNA1	973	FOXC1	1436	FAM20C	1899	TXNRD2
48	CCR6	511	CALR	974	GMDS	1437	PDGFA	1900	COMT
49	SNHG28	512	TRIM21	975	WRNIP1	1438	PRKAR1B	1901	ARVCF
50	IL2	513	MTR	976	ELANH2	1439	DNAAF5	1902	TANGO2
51	FBXL19-AS1	514	ENSG0000027476<br>0	977	PI9	1440	SUN1	1903	MIR185
52	ANKRD55	515	ENSG0000027796<br>7	978	SERPINB6	1441	GET4	1904	DGCR8
53	TNFSF11	516	MIR221	979	NQO2	1442	CENTA1	1905	TRMT2A
54	MMP1	517	CD27	980	BPHL	1443	COX19	1906	RANBP1
55	SYK	518	UBE2L3	981	TUBB2A	1444	CYP2W1	1907	ZDHHC8

56	IL1A	519	MIR493HG	982	TUBB2B	1445	MIR339	1908	RTN4R
57	FCRL3	520	ENSG00000276919	983	PSMG4	1446	GPER1	1909	DGCR6L
58	TLR4	521	LBR	984	SLC22A23	1447	ZFAND2A	1910	ZNF74
59	TRAF1	522	BPI	985	FAM60B	1448	INTS1	1911	SCARF2
60	IFNG	523	ICAM3	986	PRPF4B	1449	MAFK	1912	KLHL22
61	HLA-DQB1	524	LGALS3	987	ECI2	1450	PSMG3	1913	MED15
62	IL4	525	MIR17	988	CDYL	1451	ELFN1	1914	PI4KA
63	CXCL8	526	MIR381	989	RPP40	1452	MAD1L1	1915	HCF2
64	COMP	527	ESR1	990	LYRM4	1453	MRM2	1916	SNAP29
65	IL1R1	528	CASP3	991	PPP1R3G	1454	NUDT1	1917	CRKL
66	PTGS2	529	APOE	992	FARS2	1455	SNX8	1918	AIFM3
67	MEFV	530	NEAT1	993	NRN1	1456	EIF3B	1919	LZTR1
68	CD40	531	MIR133B	994	F13A1	1457	CHST12	1920	THAP7
69	CCL2	532	TNFSF15	995	LY86	1458	GRIFIN	1921	P2RXL1
70	MTHFR	533	GCH1	996	OFC1	1459	LFNG	1922	SLC7A4
71	TNFRSF11B	534	TAPBP	997	RREB1	1460	BRAT1	1923	GGT2
72	MMP13	535	PLAUR	998	SSR1	1461	IQCE	1924	RIMBP3B
73	SAA1	536	ACTA2	999	CTAG3	1462	TTYH3	1925	HIC2
74	IL1RAPL2	537	CSN1S1	1000	RIOK1	1463	AMZ1	1926	RIMBP3C
75	FAS	538	MIR23A	1001	DSP	1464	GNA12	1927	YDJC
76	IL23R	539	HAVCR2	1002	BLOC1S5	1465	CARD11	1928	SDF2L1
77	MMP9	540	BTK	1003	EEF1E1	1466	SDK1	1929	MIR130B
78	COL2A1	541	BANK1	1004	SLC35B3	1467	STBMS1	1930	PPIL2
79	FCGR3A	542	LRP5	1005	HULC	1468	FOXK1	1931	YPEL1
80	MACIR	543	TRPV1	1006	OFCC1	1469	AP5Z1	1932	PPM1F
81	VEGFA	544	ADA2	1007	TFAP2A	1470	RADIL	1933	TOP3B
82	IL15	545	PDE4A	1008	TFAP2AAS2	1471	PAPOLB	1934	VPREB1
83	TGFB1	546	AIF1	1009	GCNT2	1472	MMD2	1935	PRAME
84	IL6ST	547	PTEN	1010	PAK1IP1	1473	RBAK	1936	GGTLC2
85	TLR2	548	GDF5	1011	TMEM14C	1474	WIPI2	1937	MIR650
86	TIMP1	549	BLTP1	1012	TMEM14B	1475	SLC29A4	1938	IGLC1
87	FCGR2A	550	LINC00426	1013	MAK	1476	FBXL18	1939	RSPH14

88	VCAM1	551	LINC02384	1014	GCM2	1477	ACTB	1940	GNAZ
89	CD40LG	552	SH2B3	1015	SYCP2L	1478	FSCN1	1941	RAB36
90	BLK	553	MS4A1	1016	ELOVL2	1479	RNF216	1942	BCR
91	ACP5	554	MMEL1	1017	ERVFRD-1	1480	OCM	1943	IGLL1
92	S100A9	555	EPO	1018	NEDD9	1481	PMS2	1944	RGL4
93	CCR5	556	CCHCR1	1019	ADTRP	1482	AIMP2	1945	ZNF70
94	SLC11A1	557	CD163	1020	HIVEP1	1483	EIF2AK1	1946	VPREB3
95	IL13	558	COL11A2	1021	PHACTR1	1484	CYTH3	1947	CHCHD10
96	ICAM1	559	HSPA8	1022	TBC1D7	1485	FAM220A	1948	MMP11
97	SERPINH1	560	MIR494	1023	GFOD1	1486	RAC1	1949	SMARCB1
98	FCGR3B	561	MIR99B	1024	ETM3	1487	DAGLB	1950	DERL3
99	HMGB1	562	COL9A3	1025	SCAR3	1488	KDELR2	1951	SLC2A11
100	MMP2	563	MIR140	1026	SCZD3	1489	GRID2IP1	1952	DDT
101	MIF-AS1	564	OPRM1	1027	TRNAM2	1490	ZNF12	1953	GSTT2
102	FOXP3	565	ENSG00000277577	1028	SIRT5	1491	C1GALT1	1954	CABIN1
103	IL6-AS1	566	CFI	1029	NOL7	1492	LOC105375159	1955	SUSD2
104	LOC126859963	567	BCL2L1	1030	RANBP9	1493	MDDC	1956	GGT5
105	NLRP3	568	MIR320A	1031	MCUR1	1494	COL28A1	1957	SPECC1L
106	IL6R	569	F3	1032	ALPQTL3	1495	MIOS	1958	UPB1
107	IL21	570	ZAP70	1033	BWQTL4	1496	RPA3	1959	GUCD1
108	IL7	571	EGF	1034	CIHL	1497	GLCCI1	1960	SNRPD3
109	CSF2	572	SELP	1035	CMAHP	1498	ICA1	1961	PIWIL3
110	HSPD1	573	NGF	1036	DYX2	1499	NXPH1	1962	SGSM1
111	BGLAP	574	CXCR2P1	1037	NBLST4	1500	COXFA4	1963	CRYBB3
112	SPP1	575	ITGB1	1038	OTSC3	1501	PHF14	1964	CRYBB2
113	LOC100287329	576	JAZF1	1039	JARID2	1502	THSD7A	1965	BRV2
114	PDCD1	577	LRBA	1040	DTNBP1	1503	TMEM106B	1966	HCHGQ3
115	SELE	578	SOD2	1041	MYLIP	1504	SCIN	1967	ITS
116	HLA-DQA1	579	GATA3	1042	GMPR	1505	ARL4A	1968	KAZA1
117	CSF1	580	CCL11	1043	ATXN1	1506	EDSS2	1969	SQTL2
118	MBL2	581	VIP	1044	RBM24	1507	FGQTL4	1970	ADRBK2

119	TNIP1	582	CXCL16	1045	CAP2	1508	ETV1	1971	SEZ6L
120	S100A8	583	PRKCH	1046	FAM8A1	1509	DGKB	1972	HPS4
121	HP	584	GGH	1047	NUP153	1510	AGMO	1973	SRRD
122	TNFRSF11A	585	PSORS1C3	1048	KIF13A	1511	MEOX2	1974	TFIP11
123	S100A12	586	DCN	1049	NHLRC1	1512	CRPPA	1975	TPST2
124	CCL5	587	ALOX5AP	1050	KDM1B	1513	SOSTDC1	1976	CRYBA4
125	CXCL10	588	TTC7A	1051	RNF144B	1514	BZW2	1977	CRYBB1
126	TNFAIP3	589	GSTT1	1052	ID4	1515	TSPAN13	1978	MIAT
127	IL23A	590	LERFS	1053	MBOAT1	1516	AGR2	1979	MN1
128	IL10RA	591	SIRT1	1054	E2F3	1517	AGR3	1980	PITPNB
129	FASLG	592	CD83	1055	CDKAL1	1518	SNX13	1981	TTC28
130	IL17RA	593	MIR197	1056	SOX4	1519	PRPS1L1	1982	CHEK2
131	FCGR2B	594	RBPJ	1057	CASC15	1520	HDAC9	1983	HSC20
132	REL	595	IGHM	1058	NBAT1	1521	TWIST1	1984	XBP1
133	FLG	596	TREX1	1059	NRSN1	1522	FERD3L	1985	ZNRF3
134	CD79A	597	MIR30A	1060	DCDC2	1523	POLR1F	1986	KREMEN1
135	FSTL1	598	TGFB2	1061	KAAG1	1524	MACC1	1987	EMID1
136	TTR	599	ADAMTS7	1062	MRS2	1525	ITGB8	1988	EWSR1
137	TNFSF13B	600	CALCR	1063	GPLD1	1526	ABCB5	1989	GAS2L1
138	BMP6	601	ADRB2	1064	ALDH5A1	1527	SP8	1990	RASL10A
139	CD4	602	VIM	1065	KIAA0319	1528	FGQTL2	1991	AP1B1
140	ADAM17	603	THBD	1066	TDP2	1529	MYCLK1	1992	RFPL1
141	SLC19A1	604	LGALS9	1067	ACOT13	1530	MYP17	1993	RFPL1S
142	GPI	605	KIT	1068	GMNN	1531	STQTL17	1994	NEFH
143	CCR1	606	CEACAM8	1069	RIPOR2	1532	SP4	1995	THOC5
144	CCL3	607	NFKBIE	1070	CARMIL1	1533	DNAH11	1996	NIPSNAP1
145	STAT3	608	PRF1	1071	SCGN	1534	CDCA7L	1997	NF2
146	PRTN3	609	TNXB	1072	SLC17A1	1535	RAPGEF5	1998	CABP7
147	RELN	610	MTOR	1073	H2AC1	1536	TOMM7	1999	ZMAT5
148	MMP14	611	SERPINA1	1074	H2BC1	1537	HYCC1	2000	UQCR10
149	HLA-C	612	COL5A2	1075	SLC17A4	1538	KLHL7	2001	ASCC2
150	ITGAV	613	AKT1	1076	SLC17A3	1539	NUP42	2002	MTMR3
151	SOCS1	614	ESR2	1077	SLC17A2	1540	GPNMB	2003	HORMAD2

152	HLA-DPB1	615	TAP1	1078	H1-1	1541	MALSU1	2004	CASTOR1
153	ADIPOQ	616	COL11A1	1079	H3C1	1542	IGF2BP3	2005	TBC1D10A
154	CD28	617	P4HA2	1080	H4C1	1543	TRA2A	2006	SF3A1
155	GAS5	618	MIR143	1081	H4C2	1544	STK31	2007	CCDC157
156	PTGS1	619	F2RL1	1082	H3C2	1545	MPP6	2008	SEC14L2
157	NOS2	620	MIR126	1083	H2AC4	1546	OSBPL3	2009	MTFP1
158	TAGAP	621	HRH2	1084	H2BC3	1547	CYCS	2010	SEC14L3
159	MICA	622	IL34	1085	H3C3	1548	C7orf31	2011	SEC14L4
160	TNFAIP6	623	ITGA2	1086	H1-2	1549	NPVF	2012	GAL3ST1
161	MMP12	624	TEK	1087	H4C3	1550	MIR148A	2013	PES1
162	ITGAM	625	SPRED2	1088	H1-6	1551	NFE2L3	2014	TCN2
163	NR4A2	626	PLAU	1089	H2BC4	1552	HNRPA2B1	2015	DUSP18
164	PADI2	627	ASPN	1090	H2AC6	1553	CBX3	2016	OSBP2
165	ATIC	628	PPARA	1091	H1-4	1554	SNX10	2017	MORC2
166	MAPK14	629	CD55	1092	H2BC5	1555	SKAP2	2018	TUG1
167	NFKBIA	630	PICSAR	1093	H2BC6	1556	HOXA1	2019	SMTN
168	CCR2	631	FLT1	1094	H4C4	1557	HOXA2	2020	SELENOM
169	MMP8	632	NPY	1095	H3C4	1558	HOXA3	2021	INPP5J
170	CHI3L1	633	FCAR	1096	H2AC7	1559	HOXAAS2	2022	PLA2G3
171	CD44	634	EDN1	1097	H2BC7	1560	HOXA4	2023	RNF185
172	IFNB1	635	TSBP1	1098	H4C5	1561	HOXAAS3	2024	LIMK2
173	IL37	636	CD2	1099	H2BC8	1562	HOXA5	2025	PIK3IP1
174	MPO	637	HAVCR1	1100	H2AC8	1563	HOXA6	2026	ZNF278
175	PHF19	638	PTGER4	1101	H3C6	1564	HOXA7	2027	DRG1
176	MAFB	639	ATM	1102	H1-3	1565	HOXA9	2028	EIF4ENIF1
177	NFKB1	640	B3GAT1	1103	H4C6	1566	MIR196B	2029	SFI1
178	CXCR3	641	MATN1	1104	H4C7	1567	HOXA10	2030	PISD
179	CXCL12	642	SOST	1105	H3C7	1568	HOXA11	2031	DEPDC5
180	ACAN	643	CYP3A4	1106	H2BC9	1569	HOXA11AS	2032	HPC6
181	LINC02605	644	TACR3	1107	H3C8	1570	HOXA13	2033	MDNS
182	BTNL2	645	ITPA	1108	H2BC10	1571	HOTTIP	2034	YWHAH
183	PTPRC	646	PRDM1	1109	H4C8	1572	EVX1	2035	SLC5A1
184	JUN	647	IL7R	1110	TRNAM1	1573	HIBADH	2036	RFPL2

185	HSPA4	648	H2AC18	1111	TRR-TCG4-1	1574	TAX1BP1	2037	SLC5A4
186	RIPK1	649	BDNF	1112	BTN3A2	1575	CREB5	2038	RFPL3
187	OLAH	650	HTR2A	1113	BTN2A2	1576	HHT4	2039	RFPL3S
188	LTF	651	PLAT	1114	BTN3A1	1577	TRIL	2040	RTCB
189	HLA-DMA	652	TNFRSF17	1115	BTN2A3	1578	CPVL	2041	BPIFC
190	RELB	653	MMP17	1116	BTN3A3	1579	CHN2	2042	CES
191	MAPK1	654	IRF8	1117	BTN2A1	1580	PRR15	2043	L1.2
192	NR3C1	655	CENPB	1118	BTN1A1	1581	WIPF3	2044	POTE22
193	CARD8	656	IBSP	1119	TRNAR2	1582	SCRN1	2045	XTES
194	CD80	657	EGFR	1120	TRNAV21	1583	FKBP14	2046	IL17R
195	HSPA5	658	MIR222	1121	ABT1	1584	FAPP2	2047	CECR1
196	OSM	659	PARN	1122	ZNF322	1585	ZNRF2	2048	KIAA1740
197	TNFRSF13C	660	ANGPT1	1123	STQTL18	1586	GGCT	2049	C22DELq11.2
198	ITGB2	661	IL9	1124	H2BC11	1587	GARS1	2050	GC2
199	STEAP4	662	HJV	1125	H2AC11	1588	CRHR2	2051	ATP6E
200	CTSK	663	KITLG	1126	H2BC12	1589	INMT	2052	KIAA1364
201	TFRC	664	IGF2	1127	H4C9	1590	AQP1	2053	PBD7A
202	STAT1	665	C3	1128	H2AC12	1591	GHRHR	2054	TUBAL2
203	BSG	666	MIR210	1129	TRNAV12	1592	ADCYAP1R1	2055	UBP43
204	ALB	667	UNC13D	1130	PRSS16	1593	NEUROD6	2056	RIMBP3A
205	SIAE	668	TAC1	1131	ZNF184	1594	PDE1C	2057	PRODH2
206	PIK3CG	669	UBA1	1132	TRS-AGA2-3	1595	GSBS	2058	LINC00037
207	RETN	670	ATXN2	1133	TRS-TGA4-1	1596	LSM5	2059	IDD
208	TRAF6	671	LTB	1134	TRS-TGA2-1	1597	AVL9	2060	DGSI
209	MICB	672	MIR10A	1135	TRNAV17	1598	KBTBD2	2061	STK22B
210	MAPK8	673	NLRC4	1136	TRNAT15	1599	FKBP9	2062	GSCL
211	TLR3	674	UBASH3A	1137	H2BC13	1600	NT5C3A	2063	SLC20A3
212	ADAMTS4	675	AR	1138	H2AC13	1601	RP9	2064	CLTD
213	CD36	676	TLR5	1139	H3C10	1602	PTHB1	2065	DVL
214	ICOSLG	677	TFR2	1140	H2AC14	1603	BMPER	2066	TUPLE1

215	CCR3	678	IL20	1141	H2BC14	1604	NPSRAS1	2067	NLVCF
216	DHODH	679	IGFBP1	1142	H4C11	1605	DPY19L1	2068	CDC45L2
217	CXCL1	680	ALPL	1143	H4C12	1606	TBX20	2069	TMVCF
218	APOH	681	PARP1	1144	H2AC15	1607	EEPD1	2070	PNUTL1
219	BST1	682	MYOM2	1145	H2BC15	1608	MATCAP2	2071	BS
220	IL12B	683	CCL13	1146	H2AC16	1609	ANLN	2072	DGS
221	CXCR4	684	CST3	1147	H1-5	1610	AOAH	2073	WDR14
222	SERPINA3	685	NTAN1	1148	H3C11	1611	TRGJ@	2074	TRXR2
223	ELANE	686	NPPB	1149	H4C13	1612	TRGV@	2075	C22orf25
224	AGER	687	PNP	1150	H3C12	1613	GPR141	2076	HTF9C
225	CCL20	688	IL27	1151	H2AC17	1614	NME8	2077	HTF9A
226	IRAK1	689	TREM1	1152	C6DELpter	1615	SFRP4	2078	ZNF378
227	NAMPT	690	IRAK4	1153	LMWDSP2	1616	EPDR1	2079	NOGOR
228	TLR9	691	TGFBR2	1154	LSIRF	1617	STARD3NL	2080	SREC2
229	PLA2G2A	692	MIR499A	1155	SEC5L1	1618	TRGC2	2081	PCQAP
230	IL16	693	NOTCH4	1156	HFH1	1619	TARP	2082	PIK4CA
231	HLA-DMB	694	MIR106A	1157	FKHL6	1620	TRGC1	2083	HC2
232	FOLR2	695	DDR2	1158	LINC01379	1621	AMPH	2084	CEDNIK
233	CSF1R	696	GZMA	1159	FKHL7	1622	VPS41	2085	AIFL
234	IL11	697	ABCG2	1160	WHIP	1623	POU6F2	2086	SWNTS2
235	CRH	698	FGF1	1161	EI	1624	RALA	2087	P2XM
236	CLEC16A	699	MIR346	1162	CAP3	1625	DMP4	2088	CAT4
237	TIMP2	700	MTHFD1	1163	PI6	1626	MASNS	2089	HRG22
238	DDX39B	701	AMPD1	1164	NMOR2	1627	HEATR2	2090	UBCH7
239	CXCR5	702	RNPC3	1165	RIP1	1628	UNC84A	2091	MIRN130B
240	SAA4	703	S100A4	1166	CDCBM5	1629	C7orf20	2092	CYP60
241	CX3CR1	704	LPIN2	1167	CDCBM7	1630	CMKRL2	2093	PRKM1
242	CD86	705	LEPQTL1	1168	PAC4	1631	AIRAP	2094	POPX2
243	CD69	706	CCL19	1169	C6orf85	1632	INT1	2095	IGI
244	ANXA5	707	PTPN11	1170	X5L	1633	NFE2U	2096	MAPE
245	CCR7	708	SLX1A-SULT1A3	1171	PRP4	1634	PAC3	2097	GGTL4
246	TNFRSF6B	709	MIR214	1172	PECI	1635	PPP1R28	2098	IGLC

247	CR1	710	MRAP	1173	ISD11	1636	TXBP181	2099	RTDR1
248	CPT2	711	AIM2	1174	FARS1	1637	FTSJ2	2100	CML
249	IL22	712	ELN	1175	NRN	1638	MTH1	2101	IGO
250	GZMB	713	APOM	1176	F13A	1639	EIF3S9	2102	RGR
251	PRRC2A	714	EMSLR	1177	MD1	1640	C4ST2	2103	FTDALS2
252	B2M	715	LCK	1178	CL	1641	SCDO3	2104	STMY3
253	BTLA	716	TACR1	1179	CAGE1	1642	BAAT1	2105	SNF5
254	FOS	717	CD38	1180	RIO1	1643	KIAA1023	2106	GLUT11
255	PPP1CA	718	FAM167A	1181	KPPS2	1644	KIAA1691	2107	CAIN
256	TNFRSF10A	719	TGFBR1	1182	IO	1645	KIAA1950	2108	KIAA0376
257	CCRL2	720	ATG5	1183	ERP46	1646	CARMA1	2109	BUP1
258	CCL4	721	ACP1	1184	BLOS5	1647	MNF	2110	GGT
259	TAP2	722	SOD2-OT1	1185	PAPST2	1648	KIAA0415	2111	KIAA1941
260	MEG3	723	KIR2DS2	1186	MRDS1	1649	KIAA1849	2112	CRYB3
261	TNFSF13	724	DCLRE1C	1187	AP2TF	1650	TPAP	2113	CRYB2
262	CX3CL1	725	TNFRSF25	1188	HIPSTR	1651	PAQR10	2114	BARK2
263	FPGS	726	IDO1	1189	Ii	1652	IDDSSA	2115	KFS4
264	ANXA1	727	TLR8	1190	PIP1	1653	PMAT	2116	TIP39
265	ALOX5	728	TBX21	1191	RP62	1654	FBL18	2117	CTRCT23
266	PRG4	729	C5-OT1	1192	GCMB	1655	BRWS1	2118	CATCN3
267	ZNF354A	730	CYLD-AS1	1193	C6orf177	1656	SNL	2119	C22orf35
268	LIN54	731	ENPP1	1194	SSC2	1657	TRIAD3	2120	MGCR
269	USP50	732	TRH	1195	HEF1	1658	PMSL2	2121	TPRBK
270	FRG2C	733	RAD51B	1196	C6orf105	1659	JTV1	2122	RAD53
271	CAST	734	TRAF3IP2	1197	ZNF40	1660	HRI	2123	HSCB
272	CILP	735	RHOA	1198	ARCND3	1661	PSCD3	2124	XBP2
273	CCN6	736	MIR498	1199	KIAA1733	1662	SIPAR	2125	KIAA1133
274	ENO1	737	MIR145	1200	PIG51	1663	MRD48	2126	KRM1
275	PPP1CB	738	NOD1	1201	SCABD	1664	TSSN1	2127	EMU1
276	IL32	739	NAGLU	1202	TRNAMI2	1665	NPH1	2128	EWS
277	HTRA1	740	NFE2L2	1203	RANBPM	1666	NDUFA4	2129	GAR22
278	SELL	741	IL12RB1	1204	CCDC90A	1667	KIAA0783	2130	RRP22
279	CTSB	742	PIP4K2C	1205	HB15	1668	KIAA0960	2131	ADTB1

280	IL18R1	743	MIR31	1206	CMAH	1669	HLD16	2132	CMT2CC
281	ZFP36	744	PGF	1207	JMJ	1670	KIAA1905	2133	FMIP
282	MAP3K8	745	SERPINC1	1208	HPS7	1671	ARL4	2134	CALN2
283	CXCL9	746	CD1D	1209	MIR	1672	DGK	2135	UCRC
284	TRB	747	MIR769	1210	GMPR1	1673	TMEM195	2136	p100
285	PPP1CC	748	TSLP	1211	ATX1	1674	ISPD	2137	HILDA
286	LIF	749	NFATC1	1212	RNPC6	1675	ECTODIN	2138	GATSL3
287	SH2D1A	750	IKBKE	1213	EPM2A	1676	5MP1	2139	EPI64
288	JAM3	751	ANPEP	1214	TPMTD	1677	NET6	2140	SF3A120
289	LRRK2	752	CTSL	1215	LSD2	1678	AG2	2141	TAP
290	CXCL5	753	PDGFRB	1216	D6S231E	1679	BCMP11	2142	TAP3
291	ABCB1	754	RO60	1217	CSS10	1680	RP85	2143	CST
292	CD5	755	IL17D	1218	LINC00340	1681	KIAA0713	2144	PES
293	CCR4	756	MIR141	1219	VMP	1682	PRPS3	2145	TC2
294	HFE	757	RORC	1220	RU2	1683	MITR	2146	ORP4
295	CTSG	758	IFNA2	1221	RU2AS	1684	ACS3	2147	ZCW3
296	ITGA4	759	MIR382	1222	PIGPLD	1685	NTWIST	2148	SELM
297	SOCS3	760	LOC126862264	1223	SSADH	1686	TWISTNB	2149	PIB5PA
298	C5	761	CD68	1224	TTRAP	1687	BTD	2150	SPLA2III
299	NPSR1	762	BCL2L11	1225	THEM2	1688	BWQTL1	2151	PATZ
300	ITGAL	763	CD209	1226	MGORS6	1689	MYP4	2152	NEDD3
301	SYVN1	764	SPTAN1	1227	FAM65B	1690	DNAHC11	2153	KIAA0542
302	GNAQ	765	CYP21A2	1228	LRRC16A	1691	R1	2154	PSD
303	DHFR	766	MIR203A	1229	NPT1	1692	GFR	2155	KIAA0645
304	TNFRSF9	767	HLA-DOA	1230	HISTH2AA	1693	IFNB2	2156	YWHA1
305	ADORA3	768	MIR16-1	1231	HIST1H2BA	1694	FAM126A	2157	SGLT1
306	HAS1	769	CD81	1232	NPT4	1695	RP42	2158	C22orf28
307	C4A	770	COL9A1	1233	NPT3	1696	NLP1	2159	BPIL2
308	IKBKB	771	CYP19A1	1234	HIST1H1A	1697	PLCA3	2160	ACTBL1
309	SAA2	772	ADAD1	1235	HIST1H3A	1698	C7orf30	2161	XRG3
310	FCGR1A	773	KDR	1236	HIST1H4A	1699	IMP3	2162	IMD51
311	SRF	774	SAAL1	1237	HIST1H4B	1700	VAM1	2163	VAIHS
312	KLRK1	775	TGIF1	1238	HIST1H3B	1701	DFNA5	2164	ARCL2C

313	CHUK	776	MIR486-1	1239	HIST1H2AB	1702	ORP3	2165	PBD7B
314	IL18BP	777	FCER2	1240	HIST1H2BB	1703	CYC	2166	MACTHC2
315	CCL21	778	ELP1	1241	HIST1H3C	1704	RFRP	2167	ISG43
316	TNFSF14	779	CFTR	1242	HIST1H1C	1705	MIRN148A	2168	KIAA1666
317	CDH11	780	NOTCH1	1243	HLA-H	1706	NRF3	2169	SCZD4
318	JAK3	781	COG6	1244	HIST1H4C	1707	IBMPFD2	2170	DGCR14
319	DEK	782	WDFY4	1245	HIST1H1T	1708	OPTB8	2171	CTP
320	PRSS2	783	IMPDH2	1246	HIST1H2BC	1709	SCAP2	2172	DVL1
321	IL21R	784	MIR20A	1247	HIST1H2AC	1710	HOX1F	2173	MRPL22
322	CSF3	785	STAT6	1248	HIST1H1E	1711	MCOHI	2174	MGORS7
323	LECT2	786	IL15RA	1249	HIST1H2BD	1712	HOX1E	2175	AWAL
324	LTBR	787	SNORD44	1250	HIST1H2BE	1713	HOX1D	2176	CDCREL
325	ANKH	788	CXCR6	1251	HIST1H4D	1714	HOX1C	2177	BDPLT1
326	VDR	789	DNTT	1252	HIST1H3D	1715	HOX1B	2178	CTHM
327	ERAP1	790	GRN	1253	HIST1H2AD	1716	HOX1A	2179	SELZ
328	TP53	791	TIMP3	1254	HIST1H2BF	1717	HOX1G	2180	MECRCN
329	PLA2G10	792	REN	1255	HIST1H4E	1718	MIRN196B	2181	KIAA1292
330	SIGLEC1	793	CD70	1256	HIST1H2BG	1719	HOX1H	2182	VDEGS
331	TNFRSF4	794	MIR199A1	1257	HIST1H2AE	1720	HOX1I	2183	TIG1
332	ZNF334	795	MAP3K7	1258	HIST1H3E	1721	HOX1J	2184	PMGYCHA
333	IL26	796	MBP	1259	HIST1H1D	1722	NCRNA00213	2185	SERPIND1
334	PLB1	797	MIR98	1260	HIST1H4F	1723	TXBP151	2186	NS10
335	FN1	798	MIR575	1261	HIST1H4G	1724	TIP27	2187	P2X6
336	IL17B	799	LCE3C	1262	HIST1H3F	1725	CREBPA	2188	KIAA1020
337	FPR2	800	CXCR2	1263	HIST1H2BH	1726	KIAA0644	2189	ERK2
338	HAPLN1	801	MIR483	1264	HIST1H3G	1727	HVLP	2190	CAMKP
339	IL1RAP	802	MIR543	1265	HIST1H2BI	1728	ARHGAP3	2191	VPREB
340	HLA-DRB4	803	LCE3B	1266	HIST1H4H	1729	CR16	2192	OIP4
341	SFTPD	804	MIR199B	1267	TRMI2	1730	SES1	2193	PHL
342	CAT	805	U2AF1	1268	TRNAR3	1731	EDSKSCL2	2194	IGL5
343	GOLGB1	806	ABCC1	1269	BTF4	1732	CARD4	2195	SMAJ
344	GSTM1	807	TH	1270	BTF2	1733	GCTG	2196	INI1
345	GPT	808	FLNB	1271	BTF5	1734	GARS	2197	OBLFC1

346	IL22RA1	809	PSORS1C1	1272	BTF3	1735	CRFR2	2198	GGTD
347	RELA	810	COL5A1	1273	BTF1	1736	CHIP28	2199	CATCN2
348	LINC01672	811	WIPF1	1274	BTN	1737	GHRFR	2200	CTRCT3
349	MMP19	812	KCNJ11	1275	ZNF489	1738	PACAPR	2201	GRK3
350	IL17F	813	MIR122	1276	HIST1H2BJ	1739	ATOH2	2202	NTR1
351	PRL	814	GSR	1277	HIST1H2AG	1740	HCAM3	2203	CTRCT17
352	TNFRSF18	815	ASAH1	1278	HIST1H2BK	1741	KIAA0241	2204	CEBALID
353	APOB	816	ELMO2	1279	HIST1H4I	1742	FKBP60	2205	KIAA1043
354	ICOS	817	RARRES2	1280	HIST1H2AH	1743	NT5C3	2206	CHK2
355	ACE	818	FADD	1281	TSSP	1744	PAP1	2207	JAC1
356	INS	819	CD59	1282	TRNAS2	1745	BBS9	2208	ECTD13
357	IL36RN	820	GSN	1283	TRNAS3	1746	CV2	2209	BAM22
358	WAS	821	NTRK1	1284	TRNAS1	1747	AAA1	2210	SAP114
359	IL33	822	MIR451A	1285	HIST1H2BL	1748	GPR154	2211	KIAA1186
360	MIR21	823	BRD2	1286	HIST1H2AI	1749	KIAA0877	2212	KIAA1664
361	TPMT	824	CFH	1287	HIST1H3H	1750	ASD4	2213	ZCWCC1
362	C4B	825	CCL22	1288	HIST1H2AJ	1751	CDC10	2214	SEPM
363	XIST	826	HLA-DQB2	1289	HIST1H2BM	1752	KIAA1706	2215	PIPP
364	CR2	827	IRF3	1290	HIST1H4J	1753	KIAA0895	2216	MAZR
365	HPRT1	828	TRA-TGC7-1	1291	HIST1H4K	1754	FSFS8	2217	PSSC
366	IL12A	829	TRA-TGC5-1	1292	HIST1H2AK	1755	CED12	2218	FFEVF1
367	AIRE	830	CCL17	1293	HIST1H2BN	1756	PGR13	2219	HSPC117
368	HLA-A	831	MIR99A	1294	HIST1H2AL	1757	TXNDC3	2220	SNEDS
369	VWF	832	LSM2	1295	HIST1H1B	1758	FRPHE	2221	PTORCH2
370	CERNA3	833	CCL26	1296	HIST1H3I	1759	UCC1	2222	D2L2AD
371	HMGB2	834	APRT	1297	HIST1H4L	1760	MENTHO	2223	CAFS
372	TYMS	835	ITGAX	1298	HIST1H3J	1761	SCAR29	2224	GCCD5
373	CXCL13	836	HRH4	1299	HIST1H2AM	1762	WTSL	2225	ARC105
374	PON1	837	LGALS2	1300	MKPX	1763	HINCONS	2226	SPG84
375	CASP1	838	GGT1	1301	SHEP8	1764	CILD18	2227	THPH10
376	ERAP2	839	LBH	1302	SEC5	1765	KIAA0810	2228	NS2

377	HGD	840	PDGFB	1303	FREAC2	1766	CEE	2229	NS13
378	PTGES	841	RBP4	1304	FREAC3	1767	GPR30	2230	ALL
379	MIR22	842	TNPO3	1305	SERPINB9	1768	KIAA1440	2231	VPREB2
380	LEP	843	ARID5B	1306	PTI	1769	MVA7	2232	IMMD
381	GSTP1	844	CA2	1307	RIP	1770	FJH1	2233	RDT
382	ANGPT2	845	PGR-AS1	1308	C6orf86	1771	PRT1	2234	TBHS1
383	PSMB9	846	CYTOR	1309	DRS1	1772	C7orf27	2235	CTRCT22
384	RAG2	847	THBS1	1310	C6orf149	1773	PAPA7	2236	CCA2
385	SLC22A5	848	CXCR1	1311	COXPD14	1774	BIMP3	2237	CDS1
386	IL3	849	PRDX5	1312	PPKS2	1775	SPG48	2238	SIDBA5
387	PLA2G7	850	ALOX15	1313	ENDOPDI	1776	ENT4	2239	CLAPB2
388	TLR7	851	IL1RL2	1314	MUTED	1777	ZIN	2240	PRP21
389	MIR146B	852	IL1RL1	1315	BOFS	1778	HNPCC4	2241	KIAA0852
390	IL19	853	MIR15B	1316	CTRCT13	1779	HLD17	2242	LIBF
391	MIR223	854	C5AR1	1317	HRPT4	1780	LEMSPAD	2243	CMS23
392	TYK2	855	CEBPB	1318	CASL	1781	ARNO3	2244	TGA
393	MIR125A	856	PLD4	1319	QME	1782	ACPIN1	2245	GIDID2
394	CD8A	857	RCAN1	1320	DEE70	1783	OI21	2246	AGM2
395	TRPV4	858	AHR	1321	TBC7	1784	GCTR	2247	RTPS1
396	MVK	859	ANXA3	1322	RNTMI2	1785	MC4DN21	2248	LFS2
397	MMP10	860	NCF2	1323	FMP32	1786	KIAA0718	2249	KIDAR
398	TNFSF12	861	HOXD13	1324	BL11	1787	MDDGA7	2250	CMT2Z
399	CCL18	862	NCF1	1325	DIDDF	1788	USAG1	2251	DORV
400	MMP7	863	TXNDC5	1326	SCA1	1789	RIFTD	2252	MRD15
401	ETS1	864	GRK2	1327	EPM2B	1790	HDAC7B	2253	DIGFAN
402	IGF1	865	IGFBP3	1328	AOF1	1791	SCS	2254	VCFS
403	MYC	866	CCL8	1329	KIAA1154	1792	NATO3	2255	SWNTS1
404	TMSB4X	867	B3GNT2	1330	GPIPLD	1793	CILD7	2256	DGCR
405	MUC5B	868	ACKR3	1331	C6orf32	1794	JPO2	2257	CSS3
406	PPARG	869	ADORA2A	1332	LRRC16	1795	MRGEF	2258	AAK1
407	IFNGR1	870	CLEC12A	1333	H2AFR	1796	BSF2	2259	GAK
408	BCL2	871	IKZF3	1334	TSH2B	1797	DRCTNNB1A	2260	AKR1B1
409	FRZB	872	FKBP5	1335	UAQTL4	1798	PERCHING	2261	5-LOX

410	ADA	873	CXCL2	1336	H1F1	1799	UIP1	2262	CA-I
411	HGF	874	HSD11B1	1337	H3FA	1800	KOC1	2263	CA-II
412	IFNG-AS1	875	STS	1338	H4FA	1801	PALS2	2264	HMGCR
413	TNFSF4	876	BGN	1339	H4FI	1802	KIAA0704	2265	JAK-2
414	NOS3	877	BMP4	1340	H3FL	1803	THC4	2266	MMP-1
415	IL4R	878	SMS	1341	H2AFM	1804	C7orf9	2267	Malaria DHOdehase
416	CD274	879	ABCC5	1342	H2BFF	1805	SKAP55R	2268	ATK
417	PRKCD	880	ABCC4	1343	H3FC	1806	BSAS	2269	CB2
418	PTH	881	CTSD	1344	H1F2	1807	RUSAT1	2270	G6PD
419	IL10RB	882	DDX6	1345	HFE1	1808	RHOGAP3	2271	H1R
420	APOA1	883	GRK6	1346	H4FG	1809	KIAA0193	2272	JAK-3
421	FGF2	884	HCLS1	1347	H1FT	1810	C7orf24	2273	PLA2G1B
422	BMP2	885	HOXD10	1348	H2BFL	1811	SMAD1	2274	PIK3CA
423	TNFRSF13B	886	BCL2A1	1349	H2AFL	1812	CO	2275	COX-1
424	HLA-G	887	NR4A3	1350	H1F4	1813	IGHD4	2276	S1PR1
425	PLA2G4A	888	CAV2	1351	H2BFB	1814	MATH2	2277	CTLA-4
426	CALCA	889	ABCC3	1352	H2BFH	1815	DFNA74	2278	ESRRA
427	NAT2	890	GPRC5A	1353	H4FB	1816	FKBP63	2279	IL17
428	MYD88	891	LHX2	1354	H3FB	1817	UMPH1	2280	JAK-1
429	CD58	892	MAB21L2	1355	H2AFG	1818	GPRA	2281	CD207
430	HIF1A	893	TXNIP	1356	H2BFG	1819	SPTRX2	2282	MAPK12
431	HLA-DRA	894	ABCC2	1357	H4FJ	1820	PYL	2283	OPRD1
432	PIK3CD	895	GSDME	1358	H2BFA	1821	MERP1	2284	ODF
433	PRKCQ	896	LY96	1359	H2AFA	1822	WT5	2285	PDE4D
434	CARD14	897	GART	1360	H3FD	1823	CDG2Y	2286	FUN1
435	DPP4	898	RGMB	1361	H1F3	1824	NDCAGF	2287	TSPO
436	CDKN1A	899	HOXD11	1362	H4FC	1825	MTDPS17	2288	TYR
437	IL5	900	ITGA6	1363	H4FL	1826	RMFSL	2289	TXNRD1
438	GHRL	901	LCN2	1364	H2BFJ	1827	PPBL	2290	CSF2RA
439	RAG1	902	MARCKS	1365	H3FH	1828	CAHH	2291	IL23
440	CYP2C19	903	RAB8A	1366	H2BFK	1829	LYNCH4	2292	NFKB
441	CCN1	904	PLEK	1367	H4FH	1830	GRP1	2293	CELA1

442	DKK1	905	POU3F1	1368	TRM2	1831	C7orf70	2294	COX-2
443	MIR142	906	DDIT4	1369	TRR3	1832	MDDGC7	2295	COX
444	COL9A2	907	GIN1	1370	CD277	1833	KIAA0744	2296	HIV tat
445	FGFR1	908	BAIAP2L1	1371	H2BJ	1834	CRS1	2297	ITGA4/B7
446	FBN1	909	PSG5	1372	H2AG	1835	PTFB	2298	CO5
447	CDK6	910	NDUFA4L2	1373	H2BK	1836	DNAHBL	2299	SLC46A1
448	GC	911	PTK2	1374	H4FM	1837	KIAA0277	2300	MMP-13
449	P2RX7	912	RAP2A	1375	H2AH	1838	HSF	2301	MMP-8
450	XDH	913	BDKRB2	1376	TRS2	1839	HLD5	2302	MMP-9
451	CD14	914	CXCL6	1377	TRS3	1840	VICKZ3	2303	IKKB
452	IFIH1	915	TLE3	1378	TRS1	1841	CMT2D	2304	PDE4B
453	SMAD3	916	TMPO	1379	H2BFC	1842	PSN1	2305	PNPLA6
454	SERPINE1	917	TNFAIP2	1380	H2AFC	1843	VRR1	2306	p38 alpha
455	COX5A	918	TFPI2	1381	H3FK	1844	CILD6	2307	p38 beta
456	HAMP	919	CALD1	1382	H2BFE	1845	NEDCAS	2308	CTSS
457	DNASE1L3	920	ST6GALNAC5	1383	H4FE	1846	BENTA	2309	GCR mRNA
458	TF	921	MGARP	1384	H4FD	1847	IMD11A	2310	H4R
459	CFLAR	922	B3GNT9	1385	H2BFD	1848	LGMDR20	2311	MCL1
460	RASGRP1	923	RUNX2	1386	H2AFI	1849	SWCOS		
461	HMOX1	924	SLC25A12	1387	H1F5	1850	HMN5A		
462	CASP10	925	CD3E	1388	H3FF	1851	SMAJI		
463	RUNX1	926	DNASE2	1389	H4FK	1852	PGR14		

Table S5 244 common targets between CM-related targets and RA-related predicted targets

No.	Common targets	No.	Common targets	No.	Common targets	No.	Common targets
1	ABCB1	62	CXCR2	123	ITGB1	184	PMS2
2	ABCC1	63	CYP19A1	124	JAK1	185	PNP
3	ABCG2	64	CYP1A2	125	JAK2	186	PPARA
4	ACE	65	CYP2C19	126	JAK3	187	PPARG
5	ADA	66	CYP2C9	127	JUN	188	PPP1CA
6	ADAM17	67	DDR2	128	KDR	189	PPP1CB
7	ADAMTS4	68	DDX39B	129	KIT	190	PPP1CC
8	ADAMTS5	69	DDX6	130	LCK	191	PRDX5
9	ADORA2A	70	DHFR	131	LCN2	192	PRKCD
10	ADORA3	71	DHODH	132	LGALS2	193	PRKCH
11	AHR	72	DNTT	133	LGALS3	194	PRKCQ
12	AKR1B1	73	DPP4	134	LIMK2	195	PTGER4
13	AKT1	74	EGFR	135	LRRK2	196	PTGES
14	ALB	75	EIF2AK1	136	LTF	197	PTGS1
15	ALDH5A1	76	ELANE	137	MAP3K7	198	PTGS2
16	ALOX15	77	ESR1	138	MAP3K8	199	PTK2
17	ALOX5	78	ESR2	139	MAPK1	200	PTPN11
18	ALOX5AP	79	ESRRA	140	MAPK12	201	PTPN2
19	ALPL	80	F2	141	MAPK14	202	RAC1
20	AMPD1	81	F2RL1	142	MAPK8	203	RAP2A
21	ANXA5	82	F3	143	MCL1	204	RARB
22	APRT	83	FADS1	144	MIF	205	RBP4
23	AR	84	FCAR	145	MMP1	206	REN
24	ATG5	85	FGF1	146	MMP12	207	RHOA
25	ATIC	86	FGFR1	147	MMP13	208	RTN4R
26	B3GAT1	87	FLT1	148	MMP14	209	S100A9
27	BCL2	88	FOS	149	MMP2	210	SEC14L2
28	BCL2A1	89	G6PD	150	MMP3	211	SELE
29	BCL2L1	90	GART	151	MMP7	212	SELP
30	BLK	91	GC	152	MMP8	213	SERPINA1

31	BMP2	92	GMPR	153	MMP9	214	SERPINE1
32	BPI	93	GPI	154	MPO	215	SMTN
33	BST1	94	GRK6	155	MTHFD1	216	SOD2
34	BTK	95	GSR	156	MTOR	217	SRC
35	C3	96	GSTM1	157	NFE2L2	218	STAT1
36	C5AR1	97	GSTP1	158	NFKB1	219	STAT3
37	CA2	98	HGF	159	NFKBIA	220	STAT6
38	CASP1	99	HIF1A	160	NOS2	221	STS
39	CASP3	100	HMGCR	161	NOS3	222	SYK
40	CAT	101	HMOX1	162	NQO2	223	TACR1
41	CCL4	102	HPRT1	163	NR3C1	224	TACR3
42	CCL5	103	HRH2	164	NTRK1	225	TAP1
43	CCR1	104	HRH4	165	OPRD1	226	TEK
44	CCR2	105	HSD11B1	166	OPRM1	227	TGFB2
45	CCR5	106	HSPA5	167	P2RX7	228	TGFBR1
46	CD209	107	HSPA8	168	PADI4	229	TGFBR2
47	CD38	108	HTR2A	169	PARP1	230	TLR4
48	CD3E	109	IDO1	170	PDE4B	231	TNF
49	CDK6	110	IFNGR1	171	PDE4D	232	TPMT
50	CFB	111	IGF1	172	PDGFRB	233	TREM1
51	CFTR	112	IKBKB	173	PGF	234	TRPV1
52	CHUK	113	IL15	174	PIK3CA	235	TSPO
53	COMT	114	IL17F	175	PIK3CD	236	TTR
54	CSF1R	115	IL1B	176	PIK3CG	237	TYK2
55	CTSB	116	IL2	177	PLA2G10	238	TYMS
56	CTSD	117	IMPDH2	178	PLA2G1B	239	TYR
57	CTSG	118	IRAK4	179	PLA2G2A	240	VDR
58	CTSK	119	ISG20	180	PLA2G4A	241	WAS
59	CTSL	120	ITGA4	181	PLA2G7	242	XDH
60	CTSS	121	ITGAL	182	PLAT	243	XIAP
61	CXCR1	122	ITGAV	183	PLAU	244	ZAP70

Table S6 Information of 54 core candidate nodes

	Name	Degree	MCC	DMNC	MNC
1	SRC	31	12495	0.33	30
2	PIK3CA	24	12622	0.33	24
3	PIK3CD	23	12610	0.34	23
4	JUN	22	2135	0.40	21
5	EGFR	20	8355	0.42	19
6	ESR1	20	1979	0.42	19
7	PTPN11	18	14727	0.56	17
8	PTK2	17	10978	0.47	15
9	STAT1	17	3204	0.45	17
10	MAPK1	17	1531	0.46	16
11	JAK2	16	15720	0.64	16
12	JAK1	16	4806	0.52	16
13	BCL2	16	231	0.34	15
14	NFKB1	15	177	0.38	14
15	KDR	14	5773	0.41	13
16	JAK3	14	3246	0.53	14
17	BCL2L1	14	100	0.34	12
18	TLR4	13	46	0.41	8
19	PTGS2	13	138	0.41	8
20	IKBKB	13	94	0.33	13
21	PDGFRB	12	12366	0.61	12
22	FOS	11	990	0.58	11
23	CASP3	11	51	0.32	10
24	ITGB1	10	153	0.43	9
25	CCL5	10	90	0.40	10
26	SYK	10	74	0.38	10
27	MAPK14	10	270	0.55	8
28	MAPK12	10	270	0.55	8
29	TYK2	9	1680	0.67	9

30	ESR2	9	722	0.60	9
31	PRKCQ	8	20	0.38	6
32	ZAP70	8	32	0.43	6
33	HPRT1	8	56	0.38	8
34	ATIC	8	54	0.58	5
35	CYP1A2	8	13	0.32	5
36	CYP2C9	8	130	0.41	8
37	APRT	8	32	0.43	6
38	PTPN2	7	840	0.70	7
39	IFNGR1	7	240	0.62	7
40	NFKBIA	7	54	0.48	7
41	CCL4	7	54	0.44	7
42	PTGS1	7	128	0.48	7
43	ALOX5	7	127	0.57	6
44	HIF1A	7	246	0.59	7
45	XIAP	7	34	0.37	7
46	CXCR2	6	31	0.52	5
47	CCR2	6	60	0.57	6
48	ELANE	6	31	0.52	5
49	MPO	6	31	0.52	5
50	CTSS	6	49	0.58	5
51	CTSB	6	49	0.58	5
52	CYP2C19	6	126	0.57	6
53	ALOX15	6	126	0.57	6
54	AMPD1	6	50	0.48	6

Table S7 Enriched terms of 54 core candidate targets in KEGG pathways, biological processes, cellular components, and molecular functions

Category	Term	Description	Count	%	PValue	Genes	List Total	Pop Hits	Pop Total	Fold Enrichment	Bonferroni	Benjamini	FDR
KEGG_PATHWAY	hsa04659	Th17 cell differentiation	17	31.48	1.11E-18	JUN, IFNGR1, STAT1, TYK2, FOS, MAPK14, HIF1A, NFKB1, MAPK12, NFKBIA, IKBKB, ZAP70, MAPK1, PRKCQ, JAK2, JAK3, JAK1	54	108	8644	25.20	2.23E-16	4.57E-17	1.55E-17
KEGG_PATHWAY	hsa04658	Th1 and Th2 cell differentiation	16	29.63	3.43E-18	JUN, IFNGR1, STAT1, TYK2, FOS, MAPK14, NFKB1, MAPK12, NFKBIA, IKBKB, ZAP70, MAPK1, PRKCQ, JAK2, JAK3, JAK1	54	92	8644	27.84	6.89E-16	1.15E-16	3.89E-17

KEGG_PATHWAY	hsa04620	Toll-like receptor signaling pathway	16	29.63	4.27E-17	JUN, STAT1, PIK3CD, TYK2, FOS, MAPK14, NFKB1, MAPK12, NFKBIA, IKBKB, PIK3CA, CCL5, CCL4, MAPK1, TLR4, JAK1	54	108	8644	23.71	8.58E-15	1.23E-15	4.14E-16
KEGG_PATHWAY	hsa04380	Osteoclast differentiation	15	27.78	3.71E-14	JUN, SYK, IFNGR1, STAT1, PIK3CD, TYK2, FOS, MAPK14, NFKB1, MAPK12, NFKBIA, IKBKB, PIK3CA, MAPK1, JAK1	54	135	8644	17.79	7.45E-12	6.21E-13	2.10E-13
KEGG_PATHWAY	hsa04668	TNF signaling pathway	14	25.93	9.83E-14	JUN, XIAP, PIK3CD, FOS, PTGS2, MAPK14, NFKB1, MAPK12, NFKBIA, IKBKB, PIK3CA, CASP3, CCL5, MAPK1	54	114	8644	19.66	1.98E-11	1.24E-12	4.18E-13

KEGG_PATHWAY	hsa04621	NOD-like receptor signaling pathway	16	29.63	1.71E-13	JUN, STAT1, XIAP, TYK2, MAPK14, NFKB1, MAPK12, NFKBIA, IKBKB, CCL5, BCL2, MAPK1, TLR4, JAK1, CTSB, BCL2L1	54	186	8644	13.77	3.43E-11	1.90E-12	6.44E-13
KEGG_PATHWAY	hsa04210	Apoptosis	14	25.93	9.98E-13	JUN, XIAP, PIK3CD, FOS, CTSS, NFKB1, NFKBIA, IKBKB, PIK3CA, CASP3, BCL2, MAPK1, CTSB, BCL2L1	54	136	8644	16.48	2.01E-10	8.72E-12	2.95E-12
KEGG_PATHWAY	hsa04062	Chemokine signaling pathway	15	27.78	4.98E-12	STAT1, SRC, PIK3CD, PTK2, NFKB1, NFKBIA, IKBKB, PIK3CA, CCL5, CCL4, CXCR2, MAPK1, JAK2, JAK3, CCR2	54	192	8644	12.51	1.00E-09	3.85E-11	1.30E-11

KEGG_PATHWAY	hsa04660	T cell receptor signaling pathway	13	24.07	5.35E-12	JUN, PIK3CD, PTPN11, FOS, MAPK14, NFKB1, MAPK12, NFKBIA, IKBKB, ZAP70, PIK3CA, MAPK1, PRKCQ	54	121	8644	17.20	1.08E-09	3.99E-11	1.35E-11
KEGG_PATHWAY	hsa04630	JAK-STAT signaling pathway	14	25.93	1.30E-11	PDGFRB, IFNGR1, STAT1, PIK3CD, PTPN11, TYK2, EGFR, PIK3CA, BCL2, JAK2, JAK3, PTPN2, JAK1, BCL2L1	54	166	8644	13.50	2.62E-09	8.45E-11	2.86E-11
GOTERM_BP_DIRECT	GO:0018108	Peptidyl-tyrosine phosphorylation	9	16.67	3.01E-12	PDGFRB, ZAP70, SYK, SRC, KDR, JAK2, JAK3, PTK2, EGFR	54	58	19462	55.93	4.10E-09	4.10E-09	3.65E-09
GOTERM_BP_DIRECT	GO:2000811	Negative regulation of anoikis	6	11.11	1.39E-09	ITGB1, PIK3CA, SRC, BCL2, PTK2, BCL2L1	54	19	19462	113.81	1.90E-06	5.06E-07	4.50E-07

GOTERM_BP_DIRECT	GO:0006954	Inflammatory response	13	24.07	1.60E-09	ALOX15, PIK3CD, FOS, PTGS2, NFKB1, PTGS1, IKBKB, CCL5, CCL4, CXCR2, PRKCQ, TLR4, CCR2	54	436	19462	10.75	2.18E-06	5.06E-07	4.50E-07
GOTERM_BP_DIRECT	GO:0051897	Positive regulation of protein kinase B signaling	10	18.52	1.61E-09	PDGFRB, ITGB1, PIK3CA, SRC, CCL5, KDR, PIK3CD, JAK2, PTK2, EGFR	54	185	19462	19.48	2.20E-06	5.06E-07	4.50E-07
GOTERM_BP_DIRECT	GO:0035994	Response to muscle stretch	6	11.11	1.85E-09	NFKBIA, JUN, PIK3CA, FOS, MAPK14, NFKB1	54	20	19462	108.12	2.53E-06	5.06E-07	4.50E-07
GOTERM_BP_DIRECT	GO:0098586	Cellular response to virus	8	14.81	6.77E-09	IFNGR1, CCL5, TYK2, JAK2, MAPK14, HIF1A, NFKB1, JAK1	54	95	19462	30.35	9.23E-06	1.54E-06	1.37E-06
GOTERM_BP_DIRECT	GO:0070374	Positive regulation of ERK1 and ERK2 cascade	10	18.52	1.08E-08	PDGFRB, JUN, SRC, CCL5, ALOX15, CCL4, KDR, PTPN11, TLR4, EGFR	54	230	19462	15.67	1.48E-05	2.09E-06	1.86E-06

GOTERM_BP_DIRECT	GO:0048661	Positive regulation of smooth muscle cell proliferation	7	12.96	1.23E-08	PDGFRB, PIK3CA, STAT1, CCL5, PTGS2, TLR4, ELANE	54	59	19462	42.76	1.67E-05	2.09E-06	1.86E-06
GOTERM_BP_DIRECT	GO:0006468	Protein phosphorylation	12	22.22	2.54E-08	IKBKB, ZAP70, SYK, SRC, MAPK1, PIK3CD, PRKCQ, TYK2, JAK2, JAK3, PTK2, JAK1	54	445	19462	9.72	3.46E-05	3.84E-06	3.42E-06
GOTERM_BP_DIRECT	GO:0071356	Cellular response to tumor necrosis factor	8	14.81	1.00E-07	NFKBIA, IKBKB, CCL5, CCL4, MAPK1, FOS, MAPK14, NFKB1	54	140	19462	20.59	1.37E-04	1.37E-05	1.22E-05
GOTERM_BP_DIRECT	GO:0007259	JAK-STAT cascade	6	11.11	1.24E-07	STAT1, TYK2, JAK2, JAK3, JAK1, CCR2	54	44	19462	49.15	1.69E-04	1.53E-05	1.36E-05
GOTERM_BP_DIRECT	GO:0051770	Positive regulation of nitric-oxide synthase biosynthetic process	5	9.26	1.46E-07	STAT1, KDR, JAK2, JAK3, TLR4	54	18	19462	100.11	1.99E-04	1.66E-05	1.47E-05

GOTERM_BP_DIRECT	GO:0043066	Negative regulation of apoptotic process	12	22.22	1.87E-07	PDGFRB, IKBKB, SRC, CASP3, BCL2, KDR, XIAP, MPO, PTK2, EGFR, NFKB1, BCL2L1	54	542	19462	7.98	2.54E-04	1.96E-05	1.74E-05
GOTERM_BP_DIRECT	GO:0019221	Cytokine-mediated signaling pathway	8	14.81	2.50E-07	IFNGR1, STAT1, PTPN11, TYK2, JAK2, JAK3, JAK1, CCR2	54	160	19462	18.02	3.40E-04	2.43E-05	2.17E-05
GOTERM_BP_DIRECT	GO:0050727	Regulation of inflammatory response	7	12.96	3.56E-07	ALOX5, ALOX15, XIAP, JAK2, PTGS2, ESR1, CCR2	54	103	19462	24.49	4.85E-04	3.16E-05	2.82E-05
GOTERM_BP_DIRECT	GO:0035556	Intracellular signal transduction	11	20.37	4.03E-07	ZAP70, SYK, SRC, MAPK1, PRKCQ, TYK2, JAK2, MAPK14, JAK3, MAPK12, JAK1	54	462	19462	8.58	5.49E-04	3.16E-05	2.82E-05

GOTERM_BP_DIRECT	GO:0001525	Angiogenesis	9	16.67	4.15E-07	PDGFRB, JUN, SYK, PIK3CA, KDR, MAPK14, PTGS2, HIF1A, PTK2	54	255	19462	12.72	5.65E-04	3.16E-05	2.82E-05
GOTERM_BP_DIRECT	GO:0014911	Positive regulation of smooth muscle cell migration	5	9.26	4.18E-07	PDGFRB, SRC, CCL5, BCL2, TLR4	54	23	19462	78.35	5.69E-04	3.16E-05	2.82E-05
GOTERM_BP_DIRECT	GO:0042127	Regulation of cell proliferation	8	14.81	4.76E-07	PDGFRB, NFKBIA, JUN, STAT1, XIAP, PTK2, EGFR, PTGS1	54	176	19462	16.38	6.49E-04	3.42E-05	3.04E-05
GOTERM_BP_DIRECT	GO:0046777	Protein autophosphorylation	8	14.81	5.97E-07	PDGFRB, ZAP70, SYK, SRC, KDR, JAK2, PTK2, EGFR	54	182	19462	15.84	8.14E-04	4.07E-05	3.63E-05

GOTERM_CC_DIRECT	GO:0005737	Cytoplasm	39	72.22	5.21E-11	ITGB1, SRC, ALOX15, XIAP, PIK3CD, CYP2C19, PTGS2, HIF1A, EGFR, PTGS1, IKBKB, CASP3, ALOX5, MAPK1, JAK2, JAK3, ELANE, JAK1, CCR2, PDGFRB, SYK, STAT1, PTPN11, TYK2, MAPK14, ESR1, MAPK12, PTK2, NFKB1, APRT, NFKBIA, CYP2C9, ZAP70, PIK3CA, BCL2, HPRT1, TLR4, PTPN2, BCL2L1	54	5796	20660	2.57	8.91E-09	8.91E-09	7.51E-09
GOTERM_CC_DIRECT	GO:0031234	Extrinsic component of cytoplasmic side of plasma membrane	8	14.81	7.64E-10	ZAP70, SYK, SRC, ALOX15, JAK2, JAK3, PTK2, JAK1	54	74	20660	41.36	1.31E-07	6.53E-08	5.50E-08

GOTERM_CC_DIRECT	GO:0005829	Cytosol	35	64.81	2.13E-08	SRC, ALOX15, XIAP, PIK3CD, HIF1A, IKBKB, ATIC, CASP3, ALOX5, MAPK1, JAK2, JAK3, ELANE, JAK1, CCR2, SYK, STAT1, AMPD1, PTPN11, TYK2, FOS, MAPK14, ESR1, MAPK12, PTK2, NFKB1, APRT, NFKBIA, ZAP70, PIK3CA, BCL2, PRKCQ, HPRT1, PTPN2, BCL2L1	54	5629	20660	2.38	3.64E-06	1.21E-06	1.02E-06
GOTERM_CC_DIRECT	GO:0045121	Membrane raft	8	14.81	3.26E-06	ITGB1, IKBKB, ZAP70, SRC, CASP3, KDR, JAK2, EGFR	54	249	20660	12.29	5.58E-04	1.39E-04	1.17E-04
GOTERM_CC_DIRECT	GO:0048471	Perinuclear region of cytoplasm	10	18.52	1.15E-04	ITGB1, PIK3CA, STAT1, SRC, ALOX5, TLR4, PTK2, EGFR, CTSB, CCR2	54	754	20660	5.07	1.95E-02	2.91E-03	2.45E-03

GOTERM_CC_DIRECT	GO:0005925	Focal adhesion	8	14.81	1.15E-04	PDGFRB, ITGB1, SRC, MAPK1, JAK2, PTK2, EGFR, JAK1	54	434	20660	7.05	1.95E-02	2.91E-03	2.45E-03
GOTERM_CC_DIRECT	GO:0005886	Plasma membrane	28	51.85	1.19E-04	ITGB1, SRC, ALOX15, PIK3CD, CYP2C19, EGFR, ATIC, CXCR2, KDR, MAPK1, JAK2, JAK3, JAK1, CCR2, PDGFRB, JUN, SYK, IFNGR1, TYK2, ESR1, PTK2, NFKBIA, CYP2C9, ZAP70, PIK3CA, PRKCQ, TLR4, PTPN2	54	5458	20660	1.96	2.02E-02	2.91E-03	2.45E-03
GOTERM_CC_DIRECT	GO:1904813	Ficolin-1-rich granule lumen	5	9.26	2.97E-04	ALOX5, MAPK1, MAPK14, CTSS, CTSB	54	125	20660	15.30	4.96E-02	6.36E-03	5.35E-03
GOTERM_CC_DIRECT	GO:0000791	Euchromatin	4	7.41	5.42E-04	JUN, JAK2, HIF1A, ESR1	54	62	20660	24.68	8.85E-02	1.03E-02	8.67E-03

GOTERM_CC_DIRECT	GO:0005654	Nucleoplasm	22	40.74	6.66E-04	JUN, STAT1, SRC, XIAP, PTPN11, FOS, MAPK14, MPO, HIF1A, ESR1, ESR2, NFKB1, MAPK12, APRT, NFKBIA, CASP3, ALOX5, CXCR2, BCL2, MAPK1, JAK2, PTPN2	54	4077	20660	2.06	1.08E-01	1.14E-02	9.59E-03
GOTERM_CC_DIRECT	GO:0005901	Caveola	4	7.41	9.45E-04	SRC, MAPK1, JAK2, PTGS2	54	75	20660	20.40	1.49E-01	1.47E-02	1.24E-02
GOTERM_CC_DIRECT	GO:0032991	Macromolecular complex	8	14.81	2.01E-03	SYK, STAT1, BCL2, PTPN11, PTGS2, HIF1A, ESR1, EGFR	54	702	20660	4.36	2.91E-01	2.86E-02	2.41E-02
GOTERM_CC_DIRECT	GO:0043235	Receptor complex	5	9.26	2.64E-03	PDGFRB, ITGB1, KDR, TLR4, EGFR	54	225	20660	8.50	3.64E-01	3.47E-02	2.93E-02

GOTERM_CC_DIRECT	GO:0009898	Cytoplasmic side of plasma membrane	4	7.41	2.98E-03	IKBKB, ALOX15, TYK2, JAK1	54	112	20660	13.66	4.00E-01	3.64E-02	3.07E-02
GOTERM_CC_DIRECT	GO:0034774	Secretory granule lumen	4	7.41	3.29E-03	ALOX5, MAPK14, NFKB1, APRT	54	116	20660	13.19	4.31E-01	3.75E-02	3.16E-02
GOTERM_CC_DIRECT	GO:0019897	Extrinsic component of plasma membrane	3	5.56	3.85E-03	TYK2, JAK2, JAK3	54	36	20660	31.88	4.83E-01	3.89E-02	3.28E-02
GOTERM_CC_DIRECT	GO:0090575	RNA polymerase II transcription factor complex	4	7.41	3.97E-03	JUN, STAT1, FOS, HIF1A	54	124	20660	12.34	4.94E-01	3.89E-02	3.28E-02
GOTERM_CC_DIRECT	GO:0043231	Intracellular membrane-bounded organelle	9	16.67	4.10E-03	PDGFRB, CYP2C9, CYP1A2, CYP2C19, MPO, CTSS, PTK2, ESR2, PTGS1	54	1015	20660	3.39	5.04E-01	3.89E-02	3.28E-02

GOTERM_CC_DIRECT	GO:0005634	Nucleus	26	48.15	5.03E-03	XIAP, MPO, HIF1A, EGFR, IKBKB, CASP3, KDR, MAPK1, JAK2, JAK1, PDGFRB, JUN, SYK, STAT1, PTPN11, TYK2, FOS, MAPK14, ESR1, MAPK12, PTK2, ESR2, NFKB1, NFKBIA, BCL2, PTPN2	54	6106	20660	1.63	5.78E-01	4.38E-02	3.69E-02
GOTERM_CC_DIRECT	GO:0033256	I-kappaB/NF-kappaB complex	2	3.70	5.12E-03	NFKBIA, NFKB1	54	2	20660	382.59	5.85E-01	4.38E-02	3.69E-02

GOTERM_MF_DIRECT	GO:0004712	Protein serine/threonine/tyrosine kinase activity	17	31.48	4.29E-14	PDGFRB, SYK, SRC, TYK2, MAPK14, EGFR, PTK2, MAPK12, IKBKB, ZAP70, PIK3CA, KDR, MAPK1, PRKCQ, JAK2, JAK3, JAK1	54	445	18976	13.42	1.09E-11	1.09E-11	8.53E-12
GOTERM_MF_DIRECT	GO:0004713	Protein tyrosine kinase activity	11	20.37	6.43E-13	PDGFRB, ZAP70, SYK, SRC, KDR, TYK2, JAK2, JAK3, PTK2, EGFR, JAK1	54	114	18976	33.91	1.64E-10	8.20E-11	6.40E-11
GOTERM_MF_DIRECT	GO:0019899	Enzyme binding	14	25.93	3.59E-11	PDGFRB, JUN, STAT1, SRC, PTGS2, CYP2C19, MAPK14, HIF1A, ESR1, EGFR, ESR2, NFKB1, NFKBIA, CYP1A2	54	388	18976	12.68	9.15E-09	2.76E-09	2.15E-09

GOTERM_MF_DIRECT	GO:0004715	Non-membrane spanning protein tyrosine kinase activity	8	14.81	4.32E-11	ZAP70, SYK, SRC, TYK2, JAK2, JAK3, PTK2, JAK1	54	46	18976	61.11	1.10E-08	2.76E-09	2.15E-09
GOTERM_MF_DIRECT	GO:0004672	Protein kinase activity	11	20.37	9.20E-08	IKBKB, SYK, SRC, CCL5, PRKCQ, TYK2, JAK2, MAPK14, PTK2, EGFR, MAPK12	54	384	18976	10.07	2.35E-05	4.08E-06	3.19E-06
GOTERM_MF_DIRECT	GO:0042802	Identical protein binding	20	37.04	9.60E-08	JUN, STAT1, XIAP, AMPD1, FOS, ESR1, EGFR, NFKB1, NFKBIA, IKBKB, CCL5, CCL4, KDR, BCL2, MAPK1, HPRT1, JAK2, TLR4, CCR2, BCL2L1	54	1737	18976	4.05	2.45E-05	4.08E-06	3.19E-06
GOTERM_MF_DIRECT	GO:0020037	Heme binding	8	14.81	2.38E-07	CYP2C9, SRC, CYP1A2, JAK2, CYP2C19, PTGS2, MPO, PTGS1	54	155	18976	18.14	6.07E-05	8.68E-06	6.77E-06

GOTERM_MF_DIRECT	GO:0005524	ATP binding	18	33.33	5.10E-07	PDGFRB, SYK, SRC, PIK3CD, TYK2, MAPK14, EGFR, PTK2, MAPK12, IKBKB, ZAP70, PIK3CA, KDR, MAPK1, PRKCQ, JAK2, JAK3, JAK1	54	1541	18976	4.10	1.30E-04	1.63E-05	1.27E-05
GOTERM_MF_DIRECT	GO:0031730	CCR5 chemokine receptor binding	4	7.41	7.14E-07	STAT1, CCL5, CCL4, JAK1	54	7	18976	200.80	1.82E-04	2.02E-05	1.58E-05
GOTERM_MF_DIRECT	GO:0005131	Growth hormone receptor binding	4	7.41	3.34E-06	TYK2, JAK2, JAK3, JAK1	54	11	18976	127.78	8.52E-04	8.52E-05	6.65E-05
GOTERM_MF_DIRECT	GO:0016702	Oxidoreductase activity, acting on single donors with incorporation of molecular oxygen, incorporation of two atoms of oxygen	4	7.41	7.33E-06	ALOX5, ALOX15, PTGS2, PTGS1	54	14	18976	100.40	1.87E-03	1.70E-04	1.33E-04

GOTERM_MF_DIRECT	GO:0005515	Protein binding	50	92.59	1.00E-05	ITGB1, PIK3CD, MPO, IKBKB, CASP3, KDR, JAK2, JAK3, JAK1, CCR2, CTSB, PDGFRB, SYK, IFNGR1, AMPD1, TYK2, FOS, APRT, ZAP70, PIK3CA, PRKCQ, HPRT1, TLR4, SRC, ALOX15, XIAP, PTGS2, HIF1A, EGFR, PTGS1, CCL5, ALOX5, CCL4, CXCR2, MAPK1, ELANE, JUN, STAT1, PTPN11, MAPK14, ESR1, MAPK12, ESR2, NFKB1, PTK2, NFKBIA, CYP1A2, BCL2, PTPN2, BCL2L1	54	12657	18976	1.39	2.55E-03	2.13E-04	1.66E-04
GOTERM_MF_DIRECT	GO:0019901	Protein kinase binding	10	18.52	1.29E-05	PDGFRB, IKBKB, SYK, PTPN11, JAK2, HIF1A, ESR1, PTK2, PTPN2, BCL2L1	54	523	18976	6.72	3.28E-03	2.53E-04	1.97E-04
GOTERM_MF_DIRECT	GO:0005178	Integrin binding	6	11.11	8.29E-05	ITGB1, SYK, SRC, KDR, PTK2, PTPN2	54	160	18976	13.18	2.09E-02	1.51E-03	1.18E-03

GOTERM_MF_DIRECT	GO:0019903	Protein phosphatase binding	5	9.26	1.21E-04	MAPK14, JAK3, PTK2, EGFR, JAK1	54	91	18976	19.31	3.04E-02	2.06E-03	1.61E-03
GOTERM_MF_DIRECT	GO:0042169	SH2 domain binding	4	7.41	2.03E-04	SYK, SRC, JAK2, PTK2	54	41	18976	34.28	5.06E-02	3.24E-03	2.53E-03
GOTERM_MF_DIRECT	GO:0045296	Cadherin binding	7	12.96	2.59E-04	ITGB1, ATIC, STAT1, SRC, KDR, PTPN11, EGFR	54	320	18976	7.69	6.38E-02	3.88E-03	3.03E-03
GOTERM_MF_DIRECT	GO:0001784	Phosphotyrosine binding	4	7.41	3.26E-04	ZAP70, SYK, MAPK1, PTPN11	54	48	18976	29.28	7.97E-02	4.37E-03	3.41E-03
GOTERM_MF_DIRECT	GO:0001223	Transcription coactivator binding	4	7.41	3.26E-04	STAT1, HIF1A, ESR1, NFKB1	54	48	18976	29.28	7.97E-02	4.37E-03	3.41E-03
GOTERM_MF_DIRECT	GO:0005506	Iron ion binding	5	9.26	7.16E-04	CYP2C9, ALOX5, ALOX15, CYP1A2, CYP2C19	54	145	18976	12.12	1.67E-01	9.13E-03	7.13E-03

Table S8 Kernel therapeutic targets screened by MCODE algorithm

Cluster	Score	Type	Symbol
1	6.9	Clustered	PTPN11
1	6.9	Clustered	PIK3CD
1	6.9	Clustered	JAK3
1	6.9	Clustered	SRC
1	6.9	Clustered	AKT1
1	6.9	Clustered	JUN
1	6.9	Clustered	PDGFRB
1	6.9	Clustered	PTPN2
1	6.9	Clustered	ESR1
1	6.9	Clustered	EGFR
1	6.9	Clustered	KDR
1	6.9	Clustered	TYK2
1	6.9	Clustered	PIK3CA
1	6.9	Clustered	PTK2
2	6.0	Clustered	ALOX5
2	6.0	Clustered	PTGS2
2	6.0	Clustered	CYP2C9
2	6.0	Clustered	PTGS1
2	6.0	Clustered	ALOX15
2	6.0	Clustered	CYP2C19
3	5.6	Clustered	GMPR
3	5.6	Clustered	APRT
3	5.6	Clustered	ATIC
3	5.6	Clustered	IMPDH2
3	5.6	Clustered	AMPD1
3	5.6	Clustered	HPRT1
4	4.5	Clustered	MAPK14
4	4.5	Clustered	FOS
4	4.5	Clustered	MAPK1

4	4.5	Clustered	MAPK12
4	4.5	Clustered	ESR2
5	4.2	Clustered	CXCR2
5	4.2	Clustered	ELANE
5	4.2	Clustered	CCR1
5	4.2	Clustered	MPO
5	4.2	Clustered	CCR5
5	4.2	Clustered	CTSG
5	4.2	Clustered	CCR2
5	4.2	Clustered	MAP3K7
5	4.2	Clustered	BPI
5	4.2	Clustered	CCL4
5	4.2	Clustered	XIAP
5	4.2	Clustered	MAPK8
5	4.2	Clustered	IL1B
5	4.2	Clustered	LTF
5	4.2	Clustered	CTSS
5	4.2	Clustered	CTSB
5	4.2	Clustered	TNF
5	4.2	Clustered	CTSL
5	4.2	Clustered	CASP3
5	4.2	Clustered	BCL2
5	4.2	Clustered	CTSD

Table S9 Enriched terms of different cluster genes in KEGG pathways, biological processes, cellular components, and molecular functions

Category	Term	Pathway	Count	%	PValue	FDR	Name
KEGG_PATHWAY	hsa04630	JAK-STAT signaling pathway	9	64.29	1.96E-11	6.26E-10	MCODE_1
KEGG_PATHWAY	hsa01522	Endocrine resistance	8	57.14	3.30E-11	6.26E-10	MCODE_1
KEGG_PATHWAY	hsa04510	Focal adhesion	9	64.29	9.90E-11	1.02E-09	MCODE_1
KEGG_PATHWAY	hsa05205	Proteoglycans in cancer	9	64.29	1.07E-10	1.02E-09	MCODE_1
KEGG_PATHWAY	hsa01521	EGFR tyrosine kinase inhibitor resistance	7	50.00	8.16E-10	6.20E-09	MCODE_1
KEGG_PATHWAY	hsa04012	ErbB signaling pathway	7	50.00	1.28E-09	8.10E-09	MCODE_1
KEGG_PATHWAY	hsa05208	Chemical carcinogenesis - reactive oxygen species	8	57.14	1.09E-08	5.92E-08	MCODE_1
KEGG_PATHWAY	hsa04370	VEGF signaling pathway	6	42.86	1.59E-08	7.56E-08	MCODE_1
KEGG_PATHWAY	hsa04915	Estrogen signaling pathway	7	50.00	2.32E-08	9.60E-08	MCODE_1
KEGG_PATHWAY	hsa05418	Fluid shear stress and atherosclerosis	7	50.00	2.53E-08	9.60E-08	MCODE_1
KEGG_PATHWAY	hsa05161	Hepatitis B	7	50.00	6.33E-08	2.19E-07	MCODE_1
KEGG_PATHWAY	hsa05235	PD-L1 expression and PD-1 checkpoint pathway in cancer	6	42.86	1.29E-07	4.08E-07	MCODE_1
KEGG_PATHWAY	hsa05200	Pathways in cancer	9	64.29	1.98E-07	5.68E-07	MCODE_1
KEGG_PATHWAY	hsa05231	Choline metabolism in cancer	6	42.86	2.09E-07	5.68E-07	MCODE_1
KEGG_PATHWAY	hsa04625	C-type lectin receptor signaling pathway	6	42.86	2.82E-07	6.62E-07	MCODE_1
KEGG_PATHWAY	hsa04151	PI3K-Akt signaling pathway	8	57.14	2.91E-07	6.62E-07	MCODE_1

KEGG_PATHWAY	hsa04015	Rap1 signaling pathway	7	50.00	2.96E-07	6.62E-07	MCODE_1
KEGG_PATHWAY	hsa05207	Chemical carcinogenesis - receptor activation	7	50.00	3.14E-07	6.62E-07	MCODE_1
KEGG_PATHWAY	hsa04810	Regulation of actin cytoskeleton	7	50.00	4.95E-07	9.90E-07	MCODE_1
KEGG_PATHWAY	hsa04014	Ras signaling pathway	7	50.00	5.91E-07	1.12E-06	MCODE_1
KEGG_PATHWAY	hsa05131	Shigellosis	7	50.00	7.73E-07	1.40E-06	MCODE_1
KEGG_PATHWAY	hsa04926	Relaxin signaling pathway	6	42.86	8.28E-07	1.43E-06	MCODE_1
KEGG_PATHWAY	hsa05135	Yersinia infection	6	42.86	1.12E-06	1.83E-06	MCODE_1
KEGG_PATHWAY	hsa05162	Measles	6	42.86	1.16E-06	1.83E-06	MCODE_1
KEGG_PATHWAY	hsa05224	Breast cancer	6	42.86	1.58E-06	2.39E-06	MCODE_1
KEGG_PATHWAY	hsa04072	Phospholipase D signaling pathway	6	42.86	1.64E-06	2.39E-06	MCODE_1
KEGG_PATHWAY	hsa05211	Renal cell carcinoma	5	35.71	2.59E-06	3.59E-06	MCODE_1
KEGG_PATHWAY	hsa05230	Central carbon metabolism in cancer	5	35.71	2.74E-06	3.59E-06	MCODE_1
KEGG_PATHWAY	hsa04917	Prolactin signaling pathway	5	35.71	2.74E-06	3.59E-06	MCODE_1
KEGG_PATHWAY	hsa05223	Non-small cell lung cancer	5	35.71	3.07E-06	3.76E-06	MCODE_1
KEGG_PATHWAY	hsa05218	Melanoma	5	35.71	3.07E-06	3.76E-06	MCODE_1
KEGG_PATHWAY	hsa05214	Glioma	5	35.71	3.62E-06	4.30E-06	MCODE_1
KEGG_PATHWAY	hsa05165	Human papillomavirus infection	7	50.00	4.28E-06	4.93E-06	MCODE_1
KEGG_PATHWAY	hsa04062	Chemokine signaling pathway	6	42.86	5.91E-06	6.60E-06	MCODE_1

KEGG_PATHWAY	hsa05167	Kaposi sarcoma-associated herpesvirus infection	6	42.86	6.21E-06	6.61E-06	MCODE_1
KEGG_PATHWAY	hsa05210	Colorectal cancer	5	35.71	6.26E-06	6.61E-06	MCODE_1
KEGG_PATHWAY	hsa05169	Epstein-Barr virus infection	6	42.86	7.57E-06	7.78E-06	MCODE_1
KEGG_PATHWAY	hsa05215	Prostate cancer	5	35.71	1.01E-05	1.01E-05	MCODE_1
KEGG_PATHWAY	hsa05417	Lipid and atherosclerosis	6	42.86	1.03E-05	1.03E-05	MCODE_1
KEGG_PATHWAY	hsa05163	Human cytomegalovirus infection	6	42.86	1.28E-05	1.28E-05	MCODE_1
KEGG_PATHWAY	hsa04620	Toll-like receptor signaling pathway	5	35.71	1.55E-05	1.55E-05	MCODE_1
KEGG_PATHWAY	hsa04722	Neurotrophin signaling pathway	5	35.71	2.28E-05	2.28E-05	MCODE_1
KEGG_PATHWAY	hsa04919	Thyroid hormone signaling pathway	5	35.71	2.43E-05	2.43E-05	MCODE_1
KEGG_PATHWAY	hsa04660	T cell receptor signaling pathway	5	35.71	2.43E-05	2.43E-05	MCODE_1
KEGG_PATHWAY	hsa04380	Osteoclast differentiation	5	35.71	3.74E-05	3.74E-05	MCODE_1
KEGG_PATHWAY	hsa05160	Hepatitis C	5	35.71	6.94E-05	6.94E-05	MCODE_1
KEGG_PATHWAY	hsa05213	Endometrial cancer	4	28.57	7.98E-05	7.98E-05	MCODE_1
KEGG_PATHWAY	hsa04360	Axon guidance	5	35.71	1.20E-04	1.20E-04	MCODE_1
KEGG_PATHWAY	hsa05120	Epithelial cell signaling in Helicobacter pylori infection	4	28.57	1.40E-04	1.40E-04	MCODE_1
KEGG_PATHWAY	hsa05212	Pancreatic cancer	4	28.57	1.79E-04	1.79E-04	MCODE_1
KEGG_PATHWAY	hsa05220	Chronic myeloid leukemia	4	28.57	1.79E-04	1.79E-04	MCODE_1
KEGG_PATHWAY	hsa05100	Bacterial invasion of epithelial cells	4	28.57	1.86E-04	1.86E-04	MCODE_1

KEGG_PATHWAY	hsa05203	Viral carcinogenesis	5	35.71	1.87E-04	1.87E-04	MCODE_1
KEGG_PATHWAY	hsa05170	Human immunodeficiency virus 1 infection	5	35.71	2.17E-04	2.17E-04	MCODE_1
KEGG_PATHWAY	hsa04662	B cell receptor signaling pathway	4	28.57	2.41E-04	2.41E-04	MCODE_1
KEGG_PATHWAY	hsa05166	Human T-cell leukemia virus 1 infection	5	35.71	2.59E-04	2.59E-04	MCODE_1
KEGG_PATHWAY	hsa05171	Coronavirus disease - COVID-19	5	35.71	3.06E-04	3.06E-04	MCODE_1
KEGG_PATHWAY	hsa05222	Small cell lung cancer	4	28.57	3.15E-04	3.15E-04	MCODE_1
KEGG_PATHWAY	hsa04933	AGE-RAGE signaling pathway in diabetic complications	4	28.57	4.03E-04	4.03E-04	MCODE_1
KEGG_PATHWAY	hsa05142	Chagas disease	4	28.57	4.27E-04	4.27E-04	MCODE_1
KEGG_PATHWAY	hsa04931	Insulin resistance	4	28.57	5.05E-04	5.05E-04	MCODE_1
KEGG_PATHWAY	hsa04066	HIF-1 signaling pathway	4	28.57	5.19E-04	5.19E-04	MCODE_1
KEGG_PATHWAY	hsa04668	TNF signaling pathway	4	28.57	5.92E-04	5.92E-04	MCODE_1
KEGG_PATHWAY	hsa04670	Leukocyte transendothelial migration	4	28.57	6.07E-04	6.07E-04	MCODE_1
KEGG_PATHWAY	hsa05168	Herpes simplex virus 1 infection	6	42.86	6.37E-04	6.37E-04	MCODE_1
KEGG_PATHWAY	hsa04935	Growth hormone synthesis, secretion and action	4	28.57	6.87E-04	6.87E-04	MCODE_1
KEGG_PATHWAY	hsa04611	Platelet activation	4	28.57	7.56E-04	7.56E-04	MCODE_1
KEGG_PATHWAY	hsa04010	MAPK signaling pathway	5	35.71	8.24E-04	8.24E-04	MCODE_1
KEGG_PATHWAY	hsa04068	FoxO signaling pathway	4	28.57	8.88E-04	8.88E-04	MCODE_1
KEGG_PATHWAY	hsa04210	Apoptosis	4	28.57	9.90E-04	9.90E-04	MCODE_1

KEGG_PATHWAY	hsa04550	Signaling pathways regulating pluripotency of stem cells	4	28.57	1.14E-03	1.14E-03	MCODE_1
KEGG_PATHWAY	hsa05226	Gastric cancer	4	28.57	1.29E-03	1.29E-03	MCODE_1
KEGG_PATHWAY	hsa04932	Non-alcoholic fatty liver disease	4	28.57	1.44E-03	1.44E-03	MCODE_1
KEGG_PATHWAY	hsa05225	Hepatocellular carcinoma	4	28.57	1.82E-03	1.82E-03	MCODE_1
KEGG_PATHWAY	hsa05164	Influenza A	4	28.57	1.92E-03	1.92E-03	MCODE_1
KEGG_PATHWAY	hsa04973	Carbohydrate digestion and absorption	3	21.43	2.20E-03	2.20E-03	MCODE_1
KEGG_PATHWAY	hsa04613	Neutrophil extracellular trap formation	4	28.57	2.63E-03	2.63E-03	MCODE_1
KEGG_PATHWAY	hsa04923	Regulation of lipolysis in adipocytes	3	21.43	3.33E-03	3.33E-03	MCODE_1
KEGG_PATHWAY	hsa04213	Longevity regulating pathway - multiple species	3	21.43	3.68E-03	3.68E-03	MCODE_1
KEGG_PATHWAY	hsa04929	GnRH secretion	3	21.43	4.05E-03	4.05E-03	MCODE_1
KEGG_PATHWAY	hsa04024	cAMP signaling pathway	4	28.57	4.18E-03	4.18E-03	MCODE_1
KEGG_PATHWAY	hsa05221	Acute myeloid leukemia	3	21.43	4.43E-03	4.43E-03	MCODE_1
KEGG_PATHWAY	hsa04664	Fc epsilon RI signaling pathway	3	21.43	4.56E-03	4.56E-03	MCODE_1
KEGG_PATHWAY	hsa01524	Platinum drug resistance	3	21.43	5.23E-03	5.23E-03	MCODE_1
KEGG_PATHWAY	hsa05132	Salmonella infection	4	28.57	5.56E-03	5.56E-03	MCODE_1
KEGG_PATHWAY	hsa04540	Gap junction	3	21.43	7.53E-03	7.53E-03	MCODE_1
KEGG_PATHWAY	hsa04211	Longevity regulating pathway	3	21.43	7.69E-03	7.69E-03	MCODE_1
KEGG_PATHWAY	hsa04658	Th1 and Th2 cell differentiation	3	21.43	8.20E-03	8.20E-03	MCODE_1

KEGG_PATHWAY	hsa04912	GnRH signaling pathway	3	21.43	8.38E-03	8.38E-03	MCODE_1
KEGG_PATHWAY	hsa04666	Fc gamma R-mediated phagocytosis	3	21.43	9.09E-03	9.09E-03	MCODE_1
KEGG_PATHWAY	hsa04750	Inflammatory mediator regulation of TRP channels	3	21.43	9.27E-03	9.27E-03	MCODE_1
KEGG_PATHWAY	hsa05146	Amoebiasis	3	21.43	1.00E-02	1.00E-02	MCODE_1
KEGG_PATHWAY	hsa04914	Progesterone-mediated oocyte maturation	3	21.43	1.00E-02	1.00E-02	MCODE_1
KEGG_PATHWAY	hsa05206	MicroRNAs in cancer	4	28.57	1.02E-02	1.02E-02	MCODE_1
KEGG_PATHWAY	hsa04659	Th17 cell differentiation	3	21.43	1.12E-02	1.12E-02	MCODE_1
KEGG_PATHWAY	hsa04725	Cholinergic synapse	3	21.43	1.22E-02	1.22E-02	MCODE_1
KEGG_PATHWAY	hsa04071	Sphingolipid signaling pathway	3	21.43	1.39E-02	1.39E-02	MCODE_1
KEGG_PATHWAY	hsa04152	AMPK signaling pathway	3	21.43	1.39E-02	1.39E-02	MCODE_1
KEGG_PATHWAY	hsa04650	Natural killer cell mediated cytotoxicity	3	21.43	1.52E-02	1.52E-02	MCODE_1
KEGG_PATHWAY	hsa04910	Insulin signaling pathway	3	21.43	1.76E-02	1.76E-02	MCODE_1
KEGG_PATHWAY	hsa05017	Spinocerebellar ataxia	3	21.43	1.91E-02	1.91E-02	MCODE_1
KEGG_PATHWAY	hsa04921	Oxytocin signaling pathway	3	21.43	2.19E-02	2.19E-02	MCODE_1
KEGG_PATHWAY	hsa04218	Cellular senescence	3	21.43	2.24E-02	2.24E-02	MCODE_1
KEGG_PATHWAY	hsa04150	mTOR signaling pathway	3	21.43	2.24E-02	2.24E-02	MCODE_1
KEGG_PATHWAY	hsa04140	Autophagy - animal	3	21.43	2.49E-02	2.49E-02	MCODE_1
KEGG_PATHWAY	hsa05130	Pathogenic Escherichia coli infection	3	21.43	3.49E-02	3.49E-02	MCODE_1

KEGG_PATHWAY	hsa05415	Diabetic cardiomyopathy	3	21.43	3.66E-02	3.66E-02	MCODE_1
KEGG_PATHWAY	hsa04020	Calcium signaling pathway	3	21.43	5.45E-02	5.45E-02	MCODE_1
KEGG_PATHWAY	hsa04960	Aldosterone-regulated sodium reabsorption	2	14.29	5.46E-02	5.46E-02	MCODE_1
KEGG_PATHWAY	hsa05219	Bladder cancer	2	14.29	6.04E-02	6.04E-02	MCODE_1
KEGG_PATHWAY	hsa04930	Type II diabetes mellitus	2	14.29	6.75E-02	6.75E-02	MCODE_1
KEGG_PATHWAY	hsa04920	Adipocytokine signaling pathway	2	14.29	9.96E-02	9.96E-02	MCODE_1
KEGG_PATHWAY	hsa00590	Arachidonic acid metabolism	6	100.00	1.53E-11	4.90E-10	MCODE_2
KEGG_PATHWAY	hsa04726	Serotonergic synapse	6	100.00	3.95E-10	6.32E-09	MCODE_2
KEGG_PATHWAY	hsa00591	Linoleic acid metabolism	3	50.00	1.17E-04	1.25E-03	MCODE_2
KEGG_PATHWAY	hsa01100	Metabolic pathways	6	100.00	1.86E-04	1.49E-03	MCODE_2
KEGG_PATHWAY	hsa05204	Chemical carcinogenesis - DNA adducts	3	50.00	6.45E-04	4.13E-03	MCODE_2
KEGG_PATHWAY	hsa04913	Ovarian steroidogenesis	2	33.33	2.94E-02	1.52E-01	MCODE_2
KEGG_PATHWAY	hsa04923	Regulation of lipolysis in adipocytes	2	33.33	3.33E-02	1.52E-01	MCODE_2
KEGG_PATHWAY	hsa00982	Drug metabolism - cytochrome P450	2	33.33	4.12E-02	1.65E-01	MCODE_2
KEGG_PATHWAY	hsa00230	Purine metabolism	6	100.00	6.81E-10	4.09E-09	MCODE_3
KEGG_PATHWAY	hsa01232	Nucleotide metabolism	5	83.33	4.44E-08	1.33E-07	MCODE_3
KEGG_PATHWAY	hsa01100	Metabolic pathways	6	100.00	1.86E-04	3.73E-04	MCODE_3
KEGG_PATHWAY	hsa00983	Drug metabolism - other enzymes	2	33.33	4.57E-02	6.86E-02	MCODE_3

KEGG_PATHWAY	hsa04917	Prolactin signaling pathway	5	100.00	4.05E-09	2.76E-07	MCODE_4
KEGG_PATHWAY	hsa01522	Endocrine resistance	5	100.00	1.60E-08	5.43E-07	MCODE_4
KEGG_PATHWAY	hsa05133	Pertussis	4	80.00	2.65E-06	4.69E-05	MCODE_4
KEGG_PATHWAY	hsa05140	Leishmaniasis	4	80.00	2.76E-06	4.69E-05	MCODE_4
KEGG_PATHWAY	hsa05235	PD-L1 expression and PD-1 checkpoint pathway in cancer	4	80.00	4.28E-06	4.90E-05	MCODE_4
KEGG_PATHWAY	hsa04658	Th1 and Th2 cell differentiation	4	80.00	4.73E-06	4.90E-05	MCODE_4
KEGG_PATHWAY	hsa04657	IL-17 signaling pathway	4	80.00	5.04E-06	4.90E-05	MCODE_4
KEGG_PATHWAY	hsa05142	Chagas disease	4	80.00	6.46E-06	5.22E-05	MCODE_4
KEGG_PATHWAY	hsa04659	Th17 cell differentiation	4	80.00	7.67E-06	5.22E-05	MCODE_4
KEGG_PATHWAY	hsa04620	Toll-like receptor signaling pathway	4	80.00	7.67E-06	5.22E-05	MCODE_4
KEGG_PATHWAY	hsa04668	TNF signaling pathway	4	80.00	9.03E-06	5.58E-05	MCODE_4
KEGG_PATHWAY	hsa04935	Growth hormone synthesis, secretion and action	4	80.00	1.05E-05	5.65E-05	MCODE_4
KEGG_PATHWAY	hsa04660	T cell receptor signaling pathway	4	80.00	1.08E-05	5.65E-05	MCODE_4
KEGG_PATHWAY	hsa04926	Relaxin signaling pathway	4	80.00	1.31E-05	6.37E-05	MCODE_4
KEGG_PATHWAY	hsa04380	Osteoclast differentiation	4	80.00	1.50E-05	6.68E-05	MCODE_4
KEGG_PATHWAY	hsa05135	Yersinia infection	4	80.00	1.57E-05	6.68E-05	MCODE_4
KEGG_PATHWAY	hsa05161	Hepatitis B	4	80.00	2.60E-05	1.04E-04	MCODE_4
KEGG_PATHWAY	hsa05167	Kaposi sarcoma-associated herpesvirus infection	4	80.00	4.47E-05	1.69E-04	MCODE_4

KEGG_PATHWAY	hsa05130	Pathogenic Escherichia coli infection	4	80.00	4.75E-05	1.70E-04	MCODE_4
KEGG_PATHWAY	hsa05170	Human immunodeficiency virus 1 infection	4	80.00	5.83E-05	1.97E-04	MCODE_4
KEGG_PATHWAY	hsa05417	Lipid and atherosclerosis	4	80.00	6.08E-05	1.97E-04	MCODE_4
KEGG_PATHWAY	hsa05208	Chemical carcinogenesis - reactive oxygen species	4	80.00	6.78E-05	2.10E-04	MCODE_4
KEGG_PATHWAY	hsa05171	Coronavirus disease - COVID-19	4	80.00	7.64E-05	2.26E-04	MCODE_4
KEGG_PATHWAY	hsa05132	Salmonella infection	4	80.00	9.44E-05	2.67E-04	MCODE_4
KEGG_PATHWAY	hsa04010	MAPK signaling pathway	4	80.00	1.66E-04	4.52E-04	MCODE_4
KEGG_PATHWAY	hsa04370	VEGF signaling pathway	3	60.00	2.76E-04	7.22E-04	MCODE_4
KEGG_PATHWAY	hsa04664	Fc epsilon RI signaling pathway	3	60.00	3.67E-04	9.24E-04	MCODE_4
KEGG_PATHWAY	hsa04912	GnRH signaling pathway	3	60.00	6.87E-04	1.67E-03	MCODE_4
KEGG_PATHWAY	hsa04933	AGE-RAGE signaling pathway in diabetic complications	3	60.00	7.94E-04	1.86E-03	MCODE_4
KEGG_PATHWAY	hsa04914	Progesterone-mediated oocyte maturation	3	60.00	8.26E-04	1.87E-03	MCODE_4
KEGG_PATHWAY	hsa04625	C-type lectin receptor signaling pathway	3	60.00	8.58E-04	1.88E-03	MCODE_4
KEGG_PATHWAY	hsa05145	Toxoplasmosis	3	60.00	9.77E-04	2.08E-03	MCODE_4
KEGG_PATHWAY	hsa04722	Neurotrophin signaling pathway	3	60.00	1.12E-03	2.31E-03	MCODE_4
KEGG_PATHWAY	hsa04071	Sphingolipid signaling pathway	3	60.00	1.16E-03	2.32E-03	MCODE_4
KEGG_PATHWAY	hsa04611	Platelet activation	3	60.00	1.22E-03	2.37E-03	MCODE_4
KEGG_PATHWAY	hsa04068	FoxO signaling pathway	3	60.00	1.36E-03	2.47E-03	MCODE_4

KEGG_PATHWAY	hsa04114	Oocyte meiosis	3	60.00	1.36E-03	2.47E-03	MCODE_4
KEGG_PATHWAY	hsa04728	Dopaminergic synapse	3	60.00	1.38E-03	2.47E-03	MCODE_4
KEGG_PATHWAY	hsa04915	Estrogen signaling pathway	3	60.00	1.49E-03	2.59E-03	MCODE_4
KEGG_PATHWAY	hsa05418	Fluid shear stress and atherosclerosis	3	60.00	1.53E-03	2.60E-03	MCODE_4
KEGG_PATHWAY	hsa04550	Signaling pathways regulating pluripotency of stem cells	3	60.00	1.62E-03	2.68E-03	MCODE_4
KEGG_PATHWAY	hsa05224	Breast cancer	3	60.00	1.71E-03	2.74E-03	MCODE_4
KEGG_PATHWAY	hsa04723	Retrograde endocannabinoid signaling	3	60.00	1.73E-03	2.74E-03	MCODE_4
KEGG_PATHWAY	hsa04261	Adrenergic signaling in cardiomyocytes	3	60.00	1.87E-03	2.84E-03	MCODE_4
KEGG_PATHWAY	hsa04932	Non-alcoholic fatty liver disease	3	60.00	1.90E-03	2.84E-03	MCODE_4
KEGG_PATHWAY	hsa04218	Cellular senescence	3	60.00	1.92E-03	2.84E-03	MCODE_4
KEGG_PATHWAY	hsa05152	Tuberculosis	3	60.00	2.55E-03	3.69E-03	MCODE_4
KEGG_PATHWAY	hsa04621	NOD-like receptor signaling pathway	3	60.00	2.72E-03	3.86E-03	MCODE_4
KEGG_PATHWAY	hsa04613	Neutrophil extracellular trap formation	3	60.00	2.87E-03	3.98E-03	MCODE_4
KEGG_PATHWAY	hsa05205	Proteoglycans in cancer	3	60.00	3.30E-03	4.48E-03	MCODE_4
KEGG_PATHWAY	hsa04015	Rap1 signaling pathway	3	60.00	3.46E-03	4.61E-03	MCODE_4
KEGG_PATHWAY	hsa05207	Chemical carcinogenesis - receptor activation	3	60.00	3.52E-03	4.61E-03	MCODE_4
KEGG_PATHWAY	hsa05163	Human cytomegalovirus infection	3	60.00	3.96E-03	5.08E-03	MCODE_4
KEGG_PATHWAY	hsa05131	Shigellosis	3	60.00	4.76E-03	5.99E-03	MCODE_4

KEGG_PATHWAY	hsa05020	Prion disease	3	60.00	5.75E-03	7.11E-03	MCODE_4
KEGG_PATHWAY	hsa05022	Pathways of neurodegeneration - multiple diseases	3	60.00	1.71E-02	2.07E-02	MCODE_4
KEGG_PATHWAY	hsa05200	Pathways in cancer	3	60.00	2.11E-02	2.51E-02	MCODE_4
KEGG_PATHWAY	hsa04929	GnRH secretion	2	40.00	2.95E-02	3.46E-02	MCODE_4
KEGG_PATHWAY	hsa05120	Epithelial cell signaling in Helicobacter pylori infection	2	40.00	3.22E-02	3.70E-02	MCODE_4
KEGG_PATHWAY	hsa04622	RIG-I-like receptor signaling pathway	2	40.00	3.27E-02	3.70E-02	MCODE_4
KEGG_PATHWAY	hsa04662	B cell receptor signaling pathway	2	40.00	3.86E-02	4.30E-02	MCODE_4
KEGG_PATHWAY	hsa05210	Colorectal cancer	2	40.00	3.95E-02	4.33E-02	MCODE_4
KEGG_PATHWAY	hsa04713	Circadian entrainment	2	40.00	4.44E-02	4.70E-02	MCODE_4
KEGG_PATHWAY	hsa04750	Inflammatory mediator regulation of TRP channels	2	40.00	4.49E-02	4.70E-02	MCODE_4
KEGG_PATHWAY	hsa05231	Choline metabolism in cancer	2	40.00	4.49E-02	4.70E-02	MCODE_4
KEGG_PATHWAY	hsa04928	Parathyroid hormone synthesis, secretion and action	2	40.00	4.85E-02	5.00E-02	MCODE_4
KEGG_PATHWAY	hsa04725	Cholinergic synapse	2	40.00	5.16E-02	5.24E-02	MCODE_4
KEGG_PATHWAY	hsa04670	Leukocyte transendothelial migration	2	40.00	5.25E-02	5.25E-02	MCODE_4
KEGG_PATHWAY	hsa04210	Apoptosis	2	40.00	6.19E-02	6.19E-02	MCODE_4
KEGG_PATHWAY	hsa04936	Alcoholic liver disease	2	40.00	6.45E-02	6.45E-02	MCODE_4
KEGG_PATHWAY	hsa04921	Oxytocin signaling pathway	2	40.00	6.98E-02	6.98E-02	MCODE_4
KEGG_PATHWAY	hsa05169	Epstein-Barr virus infection	2	40.00	9.09E-02	9.09E-02	MCODE_4

KEGG_PATHWAY	hsa05415	Diabetic cardiomyopathy	2	40.00	9.13E-02	9.13E-02	MCODE_4
KEGG_PATHWAY	hsa05166	Human T-cell leukemia virus 1 infection	2	40.00	9.95E-02	9.95E-02	MCODE_4
KEGG_PATHWAY	hsa04210	Apoptosis	9	42.86	1.23E-10	1.10E-08	MCODE_5
KEGG_PATHWAY	hsa05145	Toxoplasmosis	7	33.33	6.67E-08	2.97E-06	MCODE_5
KEGG_PATHWAY	hsa05152	Tuberculosis	7	33.33	1.18E-06	2.62E-05	MCODE_5
KEGG_PATHWAY	hsa04621	NOD-like receptor signaling pathway	7	33.33	1.43E-06	2.62E-05	MCODE_5
KEGG_PATHWAY	hsa04061	Viral protein interaction with cytokine and cytokine receptor	6	28.57	1.47E-06	2.62E-05	MCODE_5
KEGG_PATHWAY	hsa04064	NF-kappa B signaling pathway	6	28.57	1.79E-06	2.65E-05	MCODE_5
KEGG_PATHWAY	hsa04668	TNF signaling pathway	6	28.57	2.82E-06	3.59E-05	MCODE_5
KEGG_PATHWAY	hsa05163	Human cytomegalovirus infection	7	33.33	4.32E-06	4.81E-05	MCODE_5
KEGG_PATHWAY	hsa05418	Fluid shear stress and atherosclerosis	6	28.57	7.48E-06	7.40E-05	MCODE_5
KEGG_PATHWAY	hsa04140	Autophagy - animal	6	28.57	1.73E-05	1.54E-04	MCODE_5
KEGG_PATHWAY	hsa04060	Cytokine-cytokine receptor interaction	7	33.33	2.13E-05	1.72E-04	MCODE_5
KEGG_PATHWAY	hsa04657	IL-17 signaling pathway	5	23.81	3.67E-05	2.53E-04	MCODE_5
KEGG_PATHWAY	hsa04215	Apoptosis - multiple species	4	19.05	3.69E-05	2.53E-04	MCODE_5
KEGG_PATHWAY	hsa04933	AGE-RAGE signaling pathway in diabetic complications	5	23.81	4.68E-05	2.97E-04	MCODE_5
KEGG_PATHWAY	hsa05170	Human immunodeficiency virus 1 infection	6	28.57	5.77E-05	3.31E-04	MCODE_5
KEGG_PATHWAY	hsa05417	Lipid and atherosclerosis	6	28.57	6.17E-05	3.31E-04	MCODE_5

KEGG_PATHWAY	hsa04620	Toll-like receptor signaling pathway	5	23.81	6.32E-05	3.31E-04	MCODE_5
KEGG_PATHWAY	hsa05132	Salmonella infection	6	28.57	1.24E-04	6.12E-04	MCODE_5
KEGG_PATHWAY	hsa04142	Lysosome	5	23.81	1.38E-04	6.47E-04	MCODE_5
KEGG_PATHWAY	hsa05162	Measles	5	23.81	1.64E-04	7.30E-04	MCODE_5
KEGG_PATHWAY	hsa04936	Alcoholic liver disease	5	23.81	1.83E-04	7.77E-04	MCODE_5
KEGG_PATHWAY	hsa04217	Necroptosis	5	23.81	2.83E-04	1.14E-03	MCODE_5
KEGG_PATHWAY	hsa05161	Hepatitis B	5	23.81	3.04E-04	1.18E-03	MCODE_5
KEGG_PATHWAY	hsa05133	Pertussis	4	19.05	4.94E-04	1.83E-03	MCODE_5
KEGG_PATHWAY	hsa04612	Antigen processing and presentation	4	19.05	5.34E-04	1.90E-03	MCODE_5
KEGG_PATHWAY	hsa04062	Chemokine signaling pathway	5	23.81	5.80E-04	1.99E-03	MCODE_5
KEGG_PATHWAY	hsa05130	Pathogenic Escherichia coli infection	5	23.81	6.51E-04	2.15E-03	MCODE_5
KEGG_PATHWAY	hsa05169	Epstein-Barr virus infection	5	23.81	7.02E-04	2.23E-03	MCODE_5
KEGG_PATHWAY	hsa05146	Amoebiasis	4	19.05	1.17E-03	3.58E-03	MCODE_5
KEGG_PATHWAY	hsa05131	Shigellosis	5	23.81	1.49E-03	4.41E-03	MCODE_5
KEGG_PATHWAY	hsa04071	Sphingolipid signaling pathway	4	19.05	1.91E-03	5.48E-03	MCODE_5
KEGG_PATHWAY	hsa04380	Osteoclast differentiation	4	19.05	2.61E-03	7.26E-03	MCODE_5
KEGG_PATHWAY	hsa05135	Yersinia infection	4	19.05	2.72E-03	7.34E-03	MCODE_5
KEGG_PATHWAY	hsa04010	MAPK signaling pathway	5	23.81	3.07E-03	8.03E-03	MCODE_5
KEGG_PATHWAY	hsa04932	Non-alcoholic fatty liver disease	4	19.05	3.86E-03	9.81E-03	MCODE_5

KEGG_PATHWAY	hsa05134	Legionellosis	3	14.29	5.98E-03	1.48E-02	MCODE_5
KEGG_PATHWAY	hsa04613	Neutrophil extracellular trap formation	4	19.05	6.91E-03	1.66E-02	MCODE_5
KEGG_PATHWAY	hsa05167	Kaposi sarcoma-associated herpesvirus infection	4	19.05	7.22E-03	1.69E-02	MCODE_5
KEGG_PATHWAY	hsa05120	Epithelial cell signaling in Helicobacter pylori infection	3	14.29	9.21E-03	2.10E-02	MCODE_5
KEGG_PATHWAY	hsa04622	RIG-I-like receptor signaling pathway	3	14.29	9.47E-03	2.11E-02	MCODE_5
KEGG_PATHWAY	hsa01524	Platinum drug resistance	3	14.29	9.99E-03	2.17E-02	MCODE_5
KEGG_PATHWAY	hsa04623	Cytosolic DNA-sensing pathway	3	14.29	1.05E-02	2.23E-02	MCODE_5
KEGG_PATHWAY	hsa05140	Leishmaniasis	3	14.29	1.11E-02	2.29E-02	MCODE_5
KEGG_PATHWAY	hsa05171	Coronavirus disease - COVID-19	4	19.05	1.18E-02	2.38E-02	MCODE_5
KEGG_PATHWAY	hsa05210	Colorectal cancer	3	14.29	1.37E-02	2.70E-02	MCODE_5
KEGG_PATHWAY	hsa05022	Pathways of neurodegeneration - multiple diseases	5	23.81	1.53E-02	2.94E-02	MCODE_5
KEGG_PATHWAY	hsa05222	Small cell lung cancer	3	14.29	1.55E-02	2.94E-02	MCODE_5
KEGG_PATHWAY	hsa05323	Rheumatoid arthritis	3	14.29	1.59E-02	2.94E-02	MCODE_5
KEGG_PATHWAY	hsa05020	Prion disease	4	19.05	1.80E-02	3.28E-02	MCODE_5
KEGG_PATHWAY	hsa05142	Chagas disease	3	14.29	1.89E-02	3.35E-02	MCODE_5
KEGG_PATHWAY	hsa05168	Herpes simplex virus 1 infection	5	23.81	1.96E-02	3.35E-02	MCODE_5
KEGG_PATHWAY	hsa04625	C-type lectin receptor signaling pathway	3	14.29	1.96E-02	3.35E-02	MCODE_5
KEGG_PATHWAY	hsa04660	T cell receptor signaling pathway	3	14.29	2.60E-02	4.37E-02	MCODE_5

KEGG_PATHWAY	hsa05322	Systemic lupus erythematosus	3	14.29	3.27E-02	5.39E-02	MCODE_5
KEGG_PATHWAY	hsa04145	Phagosome	3	14.29	3.96E-02	6.40E-02	MCODE_5
KEGG_PATHWAY	hsa05010	Alzheimer disease	4	19.05	4.40E-02	6.99E-02	MCODE_5
KEGG_PATHWAY	hsa05164	Influenza A	3	14.29	4.90E-02	7.65E-02	MCODE_5
KEGG_PATHWAY	hsa01523	Antifolate resistance	2	9.52	6.11E-02	9.38E-02	MCODE_5
KEGG_PATHWAY	hsa04510	Focal adhesion	3	14.29	6.64E-02	1.00E-01	MCODE_5
KEGG_PATHWAY	hsa05205	Proteoglycans in cancer	3	14.29	6.76E-02	1.00E-01	MCODE_5
KEGG_PATHWAY	hsa05143	African trypanosomiasis	2	9.52	7.49E-02	1.09E-01	MCODE_5
KEGG_PATHWAY	hsa05166	Human T-cell leukemia virus 1 infection	3	14.29	7.76E-02	1.11E-01	MCODE_5
KEGG_PATHWAY	hsa05332	Graft-versus-host disease	2	9.52	8.46E-02	1.19E-01	MCODE_5
KEGG_PATHWAY	hsa04940	Type I diabetes mellitus	2	9.52	8.65E-02	1.20E-01	MCODE_5
KEGG_PATHWAY	hsa04930	Type II diabetes mellitus	2	9.52	9.23E-02	1.26E-01	MCODE_5
KEGG_PATHWAY	hsa05200	Pathways in cancer	4	19.05	9.61E-02	1.30E-01	MCODE_5
KEGG_PATHWAY	hsa05144	Malaria	2	9.52	9.99E-02	1.33E-01	MCODE_5
GOTERM_BP_DIRECT	GO:0007173	epidermal growth factor receptor signaling pathway	6	42.86	1.28E-10	6.89E-08	MCODE_1
GOTERM_BP_DIRECT	GO:0018108	peptidyl-tyrosine phosphorylation	6	42.86	2.49E-10	6.89E-08	MCODE_1
GOTERM_BP_DIRECT	GO:0051897	positive regulation of protein kinase B signaling	7	50.00	1.10E-09	2.04E-07	MCODE_1
GOTERM_BP_DIRECT	GO:0046777	protein autophosphorylation	6	42.86	8.20E-08	1.13E-05	MCODE_1

GOTERM_BP_DIRECT	GO:0070374	positive regulation of ERK1 and ERK2 cascade	6	42.86	2.63E-07	2.91E-05	MCODE_1
GOTERM_BP_DIRECT	GO:0007169	transmembrane receptor protein tyrosine kinase signaling pathway	5	35.71	1.60E-06	1.47E-04	MCODE_1
GOTERM_BP_DIRECT	GO:0031295	T cell costimulation	4	28.57	2.83E-06	2.24E-04	MCODE_1
GOTERM_BP_DIRECT	GO:0048146	positive regulation of fibroblast proliferation	4	28.57	6.32E-06	4.15E-04	MCODE_1
GOTERM_BP_DIRECT	GO:0006468	protein phosphorylation	6	42.86	6.76E-06	4.15E-04	MCODE_1
GOTERM_BP_DIRECT	GO:0001938	positive regulation of endothelial cell proliferation	4	28.57	1.72E-05	7.61E-04	MCODE_1
GOTERM_BP_DIRECT	GO:0043066	negative regulation of apoptotic process	6	42.86	1.76E-05	7.61E-04	MCODE_1
GOTERM_BP_DIRECT	GO:0014068	positive regulation of phosphatidylinositol 3-kinase signaling	4	28.57	1.79E-05	7.61E-04	MCODE_1
GOTERM_BP_DIRECT	GO:0001525	angiogenesis	5	35.71	1.88E-05	7.61E-04	MCODE_1
GOTERM_BP_DIRECT	GO:0043491	protein kinase B signaling	4	28.57	1.93E-05	7.61E-04	MCODE_1
GOTERM_BP_DIRECT	GO:0030335	positive regulation of cell migration	5	35.71	2.25E-05	8.28E-04	MCODE_1
GOTERM_BP_DIRECT	GO:0016477	cell migration	5	35.71	2.63E-05	9.10E-04	MCODE_1
GOTERM_BP_DIRECT	GO:0010629	negative regulation of gene expression	5	35.71	4.51E-05	1.47E-03	MCODE_1
GOTERM_BP_DIRECT	GO:0038084	vascular endothelial growth factor signaling pathway	3	21.43	5.57E-05	1.71E-03	MCODE_1
GOTERM_BP_DIRECT	GO:2000811	negative regulation of anoikis	3	21.43	7.00E-05	1.96E-03	MCODE_1
GOTERM_BP_DIRECT	GO:0030154	cell differentiation	6	42.86	7.10E-05	1.96E-03	MCODE_1
GOTERM_BP_DIRECT	GO:0045737	positive regulation of cyclin-dependent protein serine/threonine kinase activity	3	21.43	7.77E-05	2.05E-03	MCODE_1

GOTERM_BP_DIRECT	GO:0038083	peptidyl-tyrosine autophosphorylation	3	21.43	9.44E-05	2.37E-03	MCODE_1
GOTERM_BP_DIRECT	GO:0019221	cytokine-mediated signaling pathway	4	28.57	1.47E-04	3.53E-03	MCODE_1
GOTERM_BP_DIRECT	GO:0048010	vascular endothelial growth factor receptor signaling pathway	3	21.43	1.77E-04	4.09E-03	MCODE_1
GOTERM_BP_DIRECT	GO:0042127	regulation of cell proliferation	4	28.57	1.95E-04	4.30E-03	MCODE_1
GOTERM_BP_DIRECT	GO:0043552	positive regulation of phosphatidylinositol 3-kinase activity	3	21.43	2.02E-04	4.30E-03	MCODE_1
GOTERM_BP_DIRECT	GO:0048008	platelet-derived growth factor receptor signaling pathway	3	21.43	2.15E-04	4.40E-03	MCODE_1
GOTERM_BP_DIRECT	GO:0071276	cellular response to cadmium ion	3	21.43	2.56E-04	5.06E-03	MCODE_1
GOTERM_BP_DIRECT	GO:0014065	phosphatidylinositol 3-kinase signaling	3	21.43	2.86E-04	5.37E-03	MCODE_1
GOTERM_BP_DIRECT	GO:0042327	positive regulation of phosphorylation	3	21.43	3.01E-04	5.37E-03	MCODE_1
GOTERM_BP_DIRECT	GO:0010634	positive regulation of epithelial cell migration	3	21.43	3.01E-04	5.37E-03	MCODE_1
GOTERM_BP_DIRECT	GO:1900182	positive regulation of protein localization to nucleus	3	21.43	3.17E-04	5.47E-03	MCODE_1
GOTERM_BP_DIRECT	GO:0008284	positive regulation of cell proliferation	5	35.71	3.56E-04	5.96E-03	MCODE_1
GOTERM_BP_DIRECT	GO:0001934	positive regulation of protein phosphorylation	4	28.57	4.06E-04	6.60E-03	MCODE_1
GOTERM_BP_DIRECT	GO:0048015	phosphatidylinositol-mediated signaling	3	21.43	4.38E-04	6.92E-03	MCODE_1
GOTERM_BP_DIRECT	GO:0071364	cellular response to epidermal growth factor stimulus	3	21.43	4.57E-04	7.01E-03	MCODE_1
GOTERM_BP_DIRECT	GO:0048013	ephrin receptor signaling pathway	3	21.43	4.96E-04	7.41E-03	MCODE_1
GOTERM_BP_DIRECT	GO:0034614	cellular response to reactive oxygen species	3	21.43	5.57E-04	8.10E-03	MCODE_1
GOTERM_BP_DIRECT	GO:0048661	positive regulation of smooth muscle cell proliferation	3	21.43	6.90E-04	9.78E-03	MCODE_1

GOTERM_BP_DIRECT	GO:0010595	positive regulation of endothelial cell migration	3	21.43	9.15E-04	1.27E-02	MCODE_1
GOTERM_BP_DIRECT	GO:0008286	insulin receptor signaling pathway	3	21.43	9.70E-04	1.31E-02	MCODE_1
GOTERM_BP_DIRECT	GO:0033674	positive regulation of kinase activity	3	21.43	1.03E-03	1.35E-02	MCODE_1
GOTERM_BP_DIRECT	GO:0045893	positive regulation of transcription, DNA-templated	5	35.71	1.07E-03	1.38E-02	MCODE_1
GOTERM_BP_DIRECT	GO:0007165	signal transduction	6	42.86	1.12E-03	1.40E-02	MCODE_1
GOTERM_BP_DIRECT	GO:0043406	positive regulation of MAP kinase activity	3	21.43	1.17E-03	1.44E-02	MCODE_1
GOTERM_BP_DIRECT	GO:0030183	B cell differentiation	3	21.43	1.63E-03	1.96E-02	MCODE_1
GOTERM_BP_DIRECT	GO:0033138	positive regulation of peptidyl-serine phosphorylation	3	21.43	1.70E-03	2.00E-02	MCODE_1
GOTERM_BP_DIRECT	GO:0070141	response to UV-A	2	14.29	2.00E-03	2.31E-02	MCODE_1
GOTERM_BP_DIRECT	GO:0007179	transforming growth factor beta receptor signaling pathway	3	21.43	2.08E-03	2.35E-02	MCODE_1
GOTERM_BP_DIRECT	GO:0032869	cellular response to insulin stimulus	3	21.43	2.20E-03	2.44E-02	MCODE_1
GOTERM_BP_DIRECT	GO:0007229	integrin-mediated signaling pathway	3	21.43	2.50E-03	2.71E-02	MCODE_1
GOTERM_BP_DIRECT	GO:0042593	glucose homeostasis	3	21.43	2.95E-03	3.14E-02	MCODE_1
GOTERM_BP_DIRECT	GO:0035556	intracellular signal transduction	4	28.57	3.18E-03	3.32E-02	MCODE_1
GOTERM_BP_DIRECT	GO:0010632	regulation of epithelial cell migration	2	14.29	4.00E-03	4.04E-02	MCODE_1
GOTERM_BP_DIRECT	GO:0007275	multicellular organism development	3	21.43	4.02E-03	4.04E-02	MCODE_1
GOTERM_BP_DIRECT	GO:0043524	negative regulation of neuron apoptotic process	3	21.43	5.12E-03	5.05E-02	MCODE_1
GOTERM_BP_DIRECT	GO:0034405	response to fluid shear stress	2	14.29	5.33E-03	5.08E-02	MCODE_1

GOTERM_BP_DIRECT	GO:0007172	signal complex assembly	2	14.29	5.33E-03	5.08E-02	MCODE_1
GOTERM_CC_DIRECT	GO:0010907	positive regulation of glucose metabolic process	2	14.29	6.00E-03	5.62E-02	MCODE_1
GOTERM_CC_DIRECT	GO:0060397	JAK-STAT cascade involved in growth hormone signaling pathway	2	14.29	6.66E-03	6.04E-02	MCODE_1
GOTERM_CC_DIRECT	GO:0010863	positive regulation of phospholipase C activity	2	14.29	6.66E-03	6.04E-02	MCODE_1
GOTERM_CC_DIRECT	GO:0045087	innate immune response	4	28.57	7.74E-03	6.91E-02	MCODE_1
GOTERM_CC_DIRECT	GO:0043276	anoikis	2	14.29	7.99E-03	7.01E-02	MCODE_1
GOTERM_CC_DIRECT	GO:0033628	regulation of cell adhesion mediated by integrin	2	14.29	8.65E-03	7.48E-02	MCODE_1
GOTERM_CC_DIRECT	GO:0048011	neurotrophin TRK receptor signaling pathway	2	14.29	9.31E-03	7.80E-02	MCODE_1
GOTERM_CC_DIRECT	GO:0061029	eyelid development in camera-type eye	2	14.29	9.31E-03	7.80E-02	MCODE_1
GOTERM_CC_DIRECT	GO:0010763	positive regulation of fibroblast migration	2	14.29	9.98E-03	8.23E-02	MCODE_1
GOTERM_CC_DIRECT	GO:0016242	negative regulation of macroautophagy	2	14.29	1.13E-02	9.07E-02	MCODE_1
GOTERM_CC_DIRECT	GO:0042981	regulation of apoptotic process	3	21.43	1.13E-02	9.07E-02	MCODE_1
GOTERM_CC_DIRECT	GO:0060065	uterus development	2	14.29	1.20E-02	9.32E-02	MCODE_1
GOTERM_CC_DIRECT	GO:0051770	positive regulation of nitric-oxide synthase biosynthetic process	2	14.29	1.20E-02	9.32E-02	MCODE_1
GOTERM_CC_DIRECT	GO:0035994	response to muscle stretch	2	14.29	1.33E-02	1.01E-01	MCODE_1
GOTERM_CC_DIRECT	GO:2000573	positive regulation of DNA biosynthetic process	2	14.29	1.33E-02	1.01E-01	MCODE_1
GOTERM_CC_DIRECT	GO:0038096	Fc-gamma receptor signaling pathway involved in phagocytosis	2	14.29	1.39E-02	1.04E-01	MCODE_1
GOTERM_CC_DIRECT	GO:0002042	cell migration involved in sprouting angiogenesis	2	14.29	1.46E-02	1.05E-01	MCODE_1

GOTERM_CC_DIRECT	GO:0046628	positive regulation of insulin receptor signaling pathway	2	14.29	1.46E-02	1.05E-01	MCODE_1
GOTERM_CC_DIRECT	GO:0036092	phosphatidylinositol-3-phosphate biosynthetic process	2	14.29	1.46E-02	1.05E-01	MCODE_1
GOTERM_CC_DIRECT	GO:0042059	negative regulation of epidermal growth factor receptor signaling pathway	2	14.29	1.53E-02	1.07E-01	MCODE_1
GOTERM_CC_DIRECT	GO:0014911	positive regulation of smooth muscle cell migration	2	14.29	1.53E-02	1.07E-01	MCODE_1
GOTERM_CC_DIRECT	GO:0016310	phosphorylation	3	21.43	1.56E-02	1.08E-01	MCODE_1
GOTERM_CC_DIRECT	GO:0036120	cellular response to platelet-derived growth factor stimulus	2	14.29	1.66E-02	1.12E-01	MCODE_1
GOTERM_CC_DIRECT	GO:0051000	positive regulation of nitric-oxide synthase activity	2	14.29	1.66E-02	1.12E-01	MCODE_1
GOTERM_CC_DIRECT	GO:2001243	negative regulation of intrinsic apoptotic signaling pathway	2	14.29	1.85E-02	1.24E-01	MCODE_1
GOTERM_CC_DIRECT	GO:0035335	peptidyl-tyrosine dephosphorylation	2	14.29	1.99E-02	1.31E-01	MCODE_1
GOTERM_CC_DIRECT	GO:0048009	insulin-like growth factor receptor signaling pathway	2	14.29	2.05E-02	1.32E-01	MCODE_1
GOTERM_CC_DIRECT	GO:0043065	positive regulation of apoptotic process	3	21.43	2.06E-02	1.32E-01	MCODE_1
GOTERM_CC_DIRECT	GO:0035924	cellular response to vascular endothelial growth factor stimulus	2	14.29	2.25E-02	1.43E-01	MCODE_1
GOTERM_CC_DIRECT	GO:0046326	positive regulation of glucose import	2	14.29	2.44E-02	1.54E-01	MCODE_1
GOTERM_CC_DIRECT	GO:0071392	cellular response to estradiol stimulus	2	14.29	2.64E-02	1.64E-01	MCODE_1
GOTERM_CC_DIRECT	GO:0030217	T cell differentiation	2	14.29	2.77E-02	1.70E-01	MCODE_1
GOTERM_CC_DIRECT	GO:0007259	JAK-STAT cascade	2	14.29	2.90E-02	1.76E-01	MCODE_1
GOTERM_CC_DIRECT	GO:0045429	positive regulation of nitric oxide biosynthetic process	2	14.29	3.10E-02	1.84E-01	MCODE_1

GOTERM_CC_DIRECT	GO:0050673	epithelial cell proliferation	2	14.29	3.10E-02	1.84E-01	MCODE_1
GOTERM_CC_DIRECT	GO:0043536	positive regulation of blood vessel endothelial cell migration	2	14.29	3.23E-02	1.90E-01	MCODE_1
GOTERM_CC_DIRECT	GO:1900087	positive regulation of G1/S transition of mitotic cell cycle	2	14.29	3.35E-02	1.93E-01	MCODE_1
GOTERM_CC_DIRECT	GO:0048839	inner ear development	2	14.29	3.35E-02	1.93E-01	MCODE_1
GOTERM_CC_DIRECT	GO:1904263	positive regulation of TORC1 signaling	2	14.29	3.42E-02	1.95E-01	MCODE_1
GOTERM_CC_DIRECT	GO:1902895	positive regulation of pri-miRNA transcription from RNA polymerase II promoter	2	14.29	3.48E-02	1.97E-01	MCODE_1
GOTERM_CC_DIRECT	GO:1904707	positive regulation of vascular smooth muscle cell proliferation	2	14.29	3.61E-02	1.97E-01	MCODE_1
GOTERM_CC_DIRECT	GO:0046854	phosphatidylinositol phosphorylation	2	14.29	3.61E-02	1.97E-01	MCODE_1
GOTERM_CC_DIRECT	GO:0030218	erythrocyte differentiation	2	14.29	3.61E-02	1.97E-01	MCODE_1
GOTERM_CC_DIRECT	GO:0043154	negative regulation of cysteine-type endopeptidase activity involved in apoptotic process	2	14.29	3.68E-02	1.97E-01	MCODE_1
GOTERM_CC_DIRECT	GO:1903078	positive regulation of protein localization to plasma membrane	2	14.29	3.68E-02	1.97E-01	MCODE_1
GOTERM_CC_DIRECT	GO:0043627	response to estrogen	2	14.29	4.13E-02	2.20E-01	MCODE_1
GOTERM_CC_DIRECT	GO:0006006	glucose metabolic process	2	14.29	4.26E-02	2.24E-01	MCODE_1
GOTERM_CC_DIRECT	GO:0042102	positive regulation of T cell proliferation	2	14.29	4.45E-02	2.29E-01	MCODE_1
GOTERM_CC_DIRECT	GO:0007611	learning or memory	2	14.29	4.45E-02	2.29E-01	MCODE_1
GOTERM_CC_DIRECT	GO:0045944	positive regulation of transcription from RNA polymerase II promoter	4	28.57	4.49E-02	2.29E-01	MCODE_1
GOTERM_CC_DIRECT	GO:0030168	platelet activation	2	14.29	4.51E-02	2.29E-01	MCODE_1

GOTERM_CC_DIRECT	GO:0001819	positive regulation of cytokine production	2	14.29	4.58E-02	2.30E-01	MCODE_1
GOTERM_CC_DIRECT	GO:0001889	liver development	2	14.29	5.41E-02	2.69E-01	MCODE_1
GOTERM_CC_DIRECT	GO:0030307	positive regulation of cell growth	2	14.29	5.98E-02	2.95E-01	MCODE_1
GOTERM_CC_DIRECT	GO:0050731	positive regulation of peptidyl-tyrosine phosphorylation	2	14.29	6.23E-02	3.05E-01	MCODE_1
GOTERM_CC_DIRECT	GO:0032355	response to estradiol	2	14.29	6.54E-02	3.17E-01	MCODE_1
GOTERM_CC_DIRECT	GO:0008584	male gonad development	2	14.29	7.23E-02	3.48E-01	MCODE_1
GOTERM_CC_DIRECT	GO:0051091	positive regulation of sequence-specific DNA binding transcription factor activity	2	14.29	7.60E-02	3.62E-01	MCODE_1
GOTERM_CC_DIRECT	GO:0090263	positive regulation of canonical Wnt signaling pathway	2	14.29	8.53E-02	4.03E-01	MCODE_1
GOTERM_CC_DIRECT	GO:0050852	T cell receptor signaling pathway	2	14.29	9.08E-02	4.26E-01	MCODE_1
GOTERM_CC_DIRECT	GO:0008360	regulation of cell shape	2	14.29	9.63E-02	4.48E-01	MCODE_1
GOTERM_CC_DIRECT	GO:0007204	positive regulation of cytosolic calcium ion concentration	2	14.29	9.88E-02	4.55E-01	MCODE_1
GOTERM_CC_DIRECT	GO:0042759	long-chain fatty acid biosynthetic process	3	50.00	1.11E-05	1.42E-03	MCODE_2
GOTERM_CC_DIRECT	GO:0001676	long-chain fatty acid metabolic process	3	50.00	2.95E-05	1.89E-03	MCODE_2
GOTERM_CC_DIRECT	GO:0050727	regulation of inflammatory response	3	50.00	2.75E-04	1.17E-02	MCODE_2
GOTERM_CC_DIRECT	GO:0016098	monoterpenoid metabolic process	2	33.33	1.80E-03	3.70E-02	MCODE_2
GOTERM_CC_DIRECT	GO:0034440	lipid oxidation	2	33.33	1.80E-03	3.70E-02	MCODE_2
GOTERM_CC_DIRECT	GO:0032787	monocarboxylic acid metabolic process	2	33.33	2.31E-03	3.70E-02	MCODE_2

GOTERM_CC_DIRECT	GO:0019371	cyclooxygenase pathway	2	33.33	2.31E-03	3.70E-02	MCODE_2
GOTERM_CC_DIRECT	GO:0051122	hepoxilin biosynthetic process	2	33.33	2.31E-03	3.70E-02	MCODE_2
GOTERM_CC_DIRECT	GO:0019372	lipoxygenase pathway	2	33.33	2.82E-03	3.94E-02	MCODE_2
GOTERM_CC_DIRECT	GO:0097267	omega-hydroxylase P450 pathway	2	33.33	3.08E-03	3.94E-02	MCODE_2
GOTERM_CC_DIRECT	GO:0006954	inflammatory response	3	50.00	4.79E-03	5.03E-02	MCODE_2
GOTERM_CC_DIRECT	GO:0001516	prostaglandin biosynthetic process	2	33.33	5.13E-03	5.03E-02	MCODE_2
GOTERM_CC_DIRECT	GO:0043651	linoleic acid metabolic process	2	33.33	5.38E-03	5.03E-02	MCODE_2
GOTERM_CC_DIRECT	GO:0019373	epoxygenase P450 pathway	2	33.33	5.64E-03	5.03E-02	MCODE_2
GOTERM_CC_DIRECT	GO:0042178	xenobiotic catabolic process	2	33.33	5.90E-03	5.03E-02	MCODE_2
GOTERM_CC_DIRECT	GO:0006082	organic acid metabolic process	2	33.33	8.71E-03	6.94E-02	MCODE_2
GOTERM_CC_DIRECT	GO:0019369	arachidonic acid metabolic process	2	33.33	9.22E-03	6.94E-02	MCODE_2
GOTERM_CC_DIRECT	GO:0030282	bone mineralization	2	33.33	1.41E-02	9.81E-02	MCODE_2
GOTERM_CC_DIRECT	GO:0008202	steroid metabolic process	2	33.33	1.46E-02	9.81E-02	MCODE_2
GOTERM_CC_DIRECT	GO:0098869	cellular oxidant detoxification	2	33.33	1.94E-02	1.24E-01	MCODE_2
GOTERM_CC_DIRECT	GO:0008217	regulation of blood pressure	2	33.33	2.11E-02	1.29E-01	MCODE_2
GOTERM_CC_DIRECT	GO:0006805	xenobiotic metabolic process	2	33.33	3.00E-02	1.74E-01	MCODE_2
GOTERM_CC_DIRECT	GO:0006979	response to oxidative stress	2	33.33	3.17E-02	1.76E-01	MCODE_2
GOTERM_CC_DIRECT	GO:0032264	IMP salvage	3	50.00	5.28E-07	1.32E-05	MCODE_3
GOTERM_CC_DIRECT	GO:0032263	GMP salvage	3	50.00	5.28E-07	1.32E-05	MCODE_3

GOTERM_CC_DIRECT	GO:0006164	purine nucleotide biosynthetic process	3	50.00	8.07E-06	1.34E-04	MCODE_3
GOTERM_CC_DIRECT	GO:0044209	AMP salvage	2	33.33	1.28E-03	1.28E-02	MCODE_3
GOTERM_CC_DIRECT	GO:0046651	lymphocyte proliferation	2	33.33	1.54E-03	1.28E-02	MCODE_3
GOTERM_CC_DIRECT	GO:0097294	'de novo' XMP biosynthetic process	2	33.33	1.80E-03	1.28E-02	MCODE_3
GOTERM_CC_DIRECT	GO:0006166	purine ribonucleoside salvage	2	33.33	1.80E-03	1.28E-02	MCODE_3
GOTERM_CC_DIRECT	GO:0006177	GMP biosynthetic process	2	33.33	2.31E-03	1.44E-02	MCODE_3
GOTERM_CC_DIRECT	GO:0007625	grooming behavior	2	33.33	3.85E-03	2.14E-02	MCODE_3
GOTERM_CC_DIRECT	GO:0009116	nucleoside metabolic process	2	33.33	5.13E-03	2.56E-02	MCODE_3
GOTERM_CC_DIRECT	GO:0071356	cellular response to tumor necrosis factor	3	60.00	3.05E-04	4.21E-02	MCODE_4
GOTERM_CC_DIRECT	GO:0018105	peptidyl-serine phosphorylation	3	60.00	5.27E-04	4.21E-02	MCODE_4
GOTERM_CC_DIRECT	GO:0007165	signal transduction	4	80.00	1.16E-03	6.17E-02	MCODE_4
GOTERM_CC_DIRECT	GO:0006357	regulation of transcription from RNA polymerase II promoter	4	80.00	2.65E-03	6.57E-02	MCODE_4
GOTERM_CC_DIRECT	GO:0051149	positive regulation of muscle cell differentiation	2	40.00	3.08E-03	6.57E-02	MCODE_4
GOTERM_CC_DIRECT	GO:0035556	intracellular signal transduction	3	60.00	3.27E-03	6.57E-02	MCODE_4
GOTERM_CC_DIRECT	GO:0030278	regulation of ossification	2	40.00	3.28E-03	6.57E-02	MCODE_4
GOTERM_CC_DIRECT	GO:0042770	signal transduction in response to DNA damage	2	40.00	3.49E-03	6.57E-02	MCODE_4
GOTERM_CC_DIRECT	GO:0051403	stress-activated MAPK cascade	2	40.00	3.90E-03	6.57E-02	MCODE_4
GOTERM_CC_DIRECT	GO:0035994	response to muscle stretch	2	40.00	4.10E-03	6.57E-02	MCODE_4

GOTERM_CC_DIRECT	GO:0071276	cellular response to cadmium ion	2	40.00	7.38E-03	1.07E-01	MCODE_4
GOTERM_CC_DIRECT	GO:0031663	lipopolysaccharide-mediated signaling pathway	2	40.00	8.60E-03	1.13E-01	MCODE_4
GOTERM_CC_DIRECT	GO:0030316	osteoclast differentiation	2	40.00	9.22E-03	1.13E-01	MCODE_4
GOTERM_CC_DIRECT	GO:0034614	cellular response to reactive oxygen species	2	40.00	1.08E-02	1.24E-01	MCODE_4
GOTERM_CC_DIRECT	GO:0032868	response to insulin	2	40.00	1.57E-02	1.68E-01	MCODE_4
GOTERM_CC_DIRECT	GO:0045944	positive regulation of transcription from RNA polymerase II promoter	3	60.00	2.20E-02	2.20E-01	MCODE_4
GOTERM_CC_DIRECT	GO:0006935	chemotaxis	2	40.00	2.54E-02	2.39E-01	MCODE_4
GOTERM_CC_DIRECT	GO:0006366	transcription from RNA polymerase II promoter	2	40.00	4.86E-02	4.32E-01	MCODE_4
GOTERM_CC_DIRECT	GO:0007166	cell surface receptor signaling pathway	2	40.00	6.59E-02	5.55E-01	MCODE_4
GOTERM_CC_DIRECT	GO:0007049	cell cycle	2	40.00	7.39E-02	5.91E-01	MCODE_4
GOTERM_CC_DIRECT	GO:0006955	immune response	12	57.14	5.09E-13	3.06E-10	MCODE_5
GOTERM_CC_DIRECT	GO:0006954	inflammatory response	8	38.10	1.63E-07	4.54E-05	MCODE_5
GOTERM_CC_DIRECT	GO:0006508	proteolysis	8	38.10	2.27E-07	4.54E-05	MCODE_5
GOTERM_CC_DIRECT	GO:0002407	dendritic cell chemotaxis	4	19.05	6.25E-07	8.54E-05	MCODE_5
GOTERM_CC_DIRECT	GO:0070098	chemokine-mediated signaling pathway	5	23.81	7.12E-07	8.54E-05	MCODE_5
GOTERM_CC_DIRECT	GO:0071222	cellular response to lipopolysaccharide	6	28.57	1.42E-06	1.42E-04	MCODE_5
GOTERM_CC_DIRECT	GO:0019722	calcium-mediated signaling	5	23.81	2.14E-06	1.83E-04	MCODE_5
GOTERM_CC_DIRECT	GO:0043066	negative regulation of apoptotic process	7	33.33	1.26E-05	9.46E-04	MCODE_5

GOTERM_CC_DIRECT	GO:0007254	JNK cascade	4	19.05	3.06E-05	2.04E-03	MCODE_5
GOTERM_CC_DIRECT	GO:0006959	humoral immune response	4	19.05	3.72E-05	2.23E-03	MCODE_5
GOTERM_CC_DIRECT	GO:0043123	positive regulation of I-kappaB kinase/NF-kappaB signaling	5	23.81	5.07E-05	2.77E-03	MCODE_5
GOTERM_CC_DIRECT	GO:0060326	cell chemotaxis	4	19.05	6.22E-05	3.11E-03	MCODE_5
GOTERM_CC_DIRECT	GO:0097067	cellular response to thyroid hormone stimulus	3	14.29	1.05E-04	4.82E-03	MCODE_5
GOTERM_CC_DIRECT	GO:0006935	chemotaxis	4	19.05	2.72E-04	1.17E-02	MCODE_5
GOTERM_CC_DIRECT	GO:0043065	positive regulation of apoptotic process	5	23.81	3.44E-04	1.37E-02	MCODE_5
GOTERM_CC_DIRECT	GO:0050830	defense response to Gram-positive bacterium	4	19.05	3.72E-04	1.39E-02	MCODE_5
GOTERM_CC_DIRECT	GO:0032496	response to lipopolysaccharide	4	19.05	4.56E-04	1.52E-02	MCODE_5
GOTERM_CC_DIRECT	GO:0019886	antigen processing and presentation of exogenous peptide antigen via MHC class II	3	14.29	4.89E-04	1.52E-02	MCODE_5
GOTERM_CC_DIRECT	GO:0007204	positive regulation of cytosolic calcium ion concentration	4	19.05	5.11E-04	1.52E-02	MCODE_5
GOTERM_CC_DIRECT	GO:0051603	proteolysis involved in cellular protein catabolic process	3	14.29	5.20E-04	1.52E-02	MCODE_5
GOTERM_CC_DIRECT	GO:0000165	MAPK cascade	4	19.05	5.31E-04	1.52E-02	MCODE_5
GOTERM_CC_DIRECT	GO:0019221	cytokine-mediated signaling pathway	4	19.05	5.61E-04	1.53E-02	MCODE_5
GOTERM_CC_DIRECT	GO:0051092	positive regulation of NF-kappaB transcription factor activity	4	19.05	6.03E-04	1.57E-02	MCODE_5
GOTERM_CC_DIRECT	GO:0032743	positive regulation of interleukin-2 production	3	14.29	6.54E-04	1.57E-02	MCODE_5
GOTERM_CC_DIRECT	GO:2001240	negative regulation of extrinsic apoptotic signaling pathway in absence of ligand	3	14.29	6.54E-04	1.57E-02	MCODE_5

GOTERM_CC_DIRECT	GO:0030574	collagen catabolic process	3	14.29	8.03E-04	1.85E-02	MCODE_5
GOTERM_CC_DIRECT	GO:0031663	lipopolysaccharide-mediated signaling pathway	3	14.29	8.43E-04	1.87E-02	MCODE_5
GOTERM_CC_DIRECT	GO:0002548	monocyte chemotaxis	3	14.29	9.67E-04	2.02E-02	MCODE_5
GOTERM_CC_DIRECT	GO:0022617	extracellular matrix disassembly	3	14.29	1.01E-03	2.02E-02	MCODE_5
GOTERM_CC_DIRECT	GO:0009411	response to UV	3	14.29	1.01E-03	2.02E-02	MCODE_5
GOTERM_CC_DIRECT	GO:0006968	cellular defense response	3	14.29	1.49E-03	2.89E-02	MCODE_5
GOTERM_CC_DIRECT	GO:0007267	cell-cell signaling	4	19.05	1.68E-03	3.12E-02	MCODE_5
GOTERM_CC_DIRECT	GO:0051384	response to glucocorticoid	3	14.29	1.71E-03	3.12E-02	MCODE_5
GOTERM_CC_DIRECT	GO:0007249	I-kappaB kinase/NF-kappaB signaling	3	14.29	1.95E-03	3.42E-02	MCODE_5
GOTERM_CC_DIRECT	GO:0032757	positive regulation of interleukin-8 production	3	14.29	2.01E-03	3.42E-02	MCODE_5
GOTERM_CC_DIRECT	GO:1990268	response to gold nanoparticle	2	9.52	2.05E-03	3.42E-02	MCODE_5
GOTERM_CC_DIRECT	GO:0042742	defense response to bacterium	4	19.05	2.30E-03	3.72E-02	MCODE_5
GOTERM_CC_DIRECT	GO:0009611	response to wounding	3	14.29	2.73E-03	4.20E-02	MCODE_5
GOTERM_CC_DIRECT	GO:0019731	antibacterial humoral response	3	14.29	2.73E-03	4.20E-02	MCODE_5
GOTERM_CC_DIRECT	GO:0043406	positive regulation of MAP kinase activity	3	14.29	2.80E-03	4.20E-02	MCODE_5
GOTERM_CC_DIRECT	GO:0032729	positive regulation of interferon-gamma production	3	14.29	2.95E-03	4.29E-02	MCODE_5
GOTERM_CC_DIRECT	GO:0048002	antigen processing and presentation of peptide antigen	2	9.52	3.08E-03	4.29E-02	MCODE_5
GOTERM_CC_DIRECT	GO:0060559	positive regulation of calcidiol 1-monooxygenase activity	2	9.52	3.08E-03	4.29E-02	MCODE_5

GOTERM_CC_DIRECT	GO:0051402	neuron apoptotic process	3	14.29	3.17E-03	4.29E-02	MCODE_5
GOTERM_CC_DIRECT	GO:0030593	neutrophil chemotaxis	3	14.29	3.25E-03	4.29E-02	MCODE_5
GOTERM_CC_DIRECT	GO:0006915	apoptotic process	5	23.81	3.29E-03	4.29E-02	MCODE_5
GOTERM_CC_DIRECT	GO:0016485	protein processing	3	14.29	3.48E-03	4.45E-02	MCODE_5
GOTERM_CC_DIRECT	GO:0030730	sequestering of triglyceride	2	9.52	4.10E-03	5.13E-02	MCODE_5
GOTERM_CC_DIRECT	GO:0050829	defense response to Gram-negative bacterium	3	14.29	4.23E-03	5.18E-02	MCODE_5
GOTERM_CC_DIRECT	GO:0007166	cell surface receptor signaling pathway	4	19.05	4.41E-03	5.29E-02	MCODE_5
GOTERM_CC_DIRECT	GO:0046330	positive regulation of JNK cascade	3	14.29	4.58E-03	5.39E-02	MCODE_5
GOTERM_CC_DIRECT	GO:0046718	viral entry into host cell	3	14.29	4.95E-03	5.71E-02	MCODE_5
GOTERM_CC_DIRECT	GO:0031622	positive regulation of fever generation	2	9.52	5.13E-03	5.81E-02	MCODE_5
GOTERM_CC_DIRECT	GO:0050729	positive regulation of inflammatory response	3	14.29	6.03E-03	6.58E-02	MCODE_5
GOTERM_CC_DIRECT	GO:0006874	cellular calcium ion homeostasis	3	14.29	6.03E-03	6.58E-02	MCODE_5
GOTERM_CC_DIRECT	GO:0090594	inflammatory response to wounding	2	9.52	7.17E-03	7.55E-02	MCODE_5
GOTERM_CC_DIRECT	GO:0010573	vascular endothelial growth factor production	2	9.52	7.17E-03	7.55E-02	MCODE_5
GOTERM_CC_DIRECT	GO:0002438	acute inflammatory response to antigenic stimulus	2	9.52	8.19E-03	8.48E-02	MCODE_5
GOTERM_CC_DIRECT	GO:1903140	regulation of establishment of endothelial barrier	2	9.52	9.21E-03	9.37E-02	MCODE_5
GOTERM_CC_DIRECT	GO:0010952	positive regulation of peptidase activity	2	9.52	1.02E-02	1.02E-01	MCODE_5
GOTERM_CC_DIRECT	GO:1903238	positive regulation of leukocyte tethering or rolling	2	9.52	1.33E-02	1.26E-01	MCODE_5

GOTERM_CC_DIRECT	GO:0002523	leukocyte migration involved in inflammatory response	2	9.52	1.33E-02	1.26E-01	MCODE_5
GOTERM_CC_DIRECT	GO:0034138	toll-like receptor 3 signaling pathway	2	9.52	1.33E-02	1.26E-01	MCODE_5
GOTERM_CC_DIRECT	GO:0001666	response to hypoxia	3	14.29	1.40E-02	1.32E-01	MCODE_5
GOTERM_CC_DIRECT	GO:0001878	response to yeast	2	9.52	1.43E-02	1.32E-01	MCODE_5
GOTERM_CC_DIRECT	GO:0034116	positive regulation of heterotypic cell-cell adhesion	2	9.52	1.53E-02	1.39E-01	MCODE_5
GOTERM_CC_DIRECT	GO:0042119	neutrophil activation	2	9.52	1.73E-02	1.53E-01	MCODE_5
GOTERM_CC_DIRECT	GO:0048143	astrocyte activation	2	9.52	1.73E-02	1.53E-01	MCODE_5
GOTERM_CC_DIRECT	GO:0051044	positive regulation of membrane protein ectodomain proteolysis	2	9.52	1.83E-02	1.57E-01	MCODE_5
GOTERM_CC_DIRECT	GO:0050995	negative regulation of lipid catabolic process	2	9.52	1.83E-02	1.57E-01	MCODE_5
GOTERM_CC_DIRECT	GO:1904019	epithelial cell apoptotic process	2	9.52	1.94E-02	1.61E-01	MCODE_5
GOTERM_CC_DIRECT	GO:0051403	stress-activated MAPK cascade	2	9.52	1.94E-02	1.61E-01	MCODE_5
GOTERM_CC_DIRECT	GO:1902004	positive regulation of beta-amyloid formation	2	9.52	2.24E-02	1.81E-01	MCODE_5
GOTERM_CC_DIRECT	GO:0060252	positive regulation of glial cell proliferation	2	9.52	2.24E-02	1.81E-01	MCODE_5
GOTERM_CC_DIRECT	GO:0070374	positive regulation of ERK1 and ERK2 cascade	3	14.29	2.30E-02	1.84E-01	MCODE_5
GOTERM_CC_DIRECT	GO:0090026	positive regulation of monocyte chemotaxis	2	9.52	2.34E-02	1.85E-01	MCODE_5
GOTERM_CC_DIRECT	GO:0071310	cellular response to organic substance	2	9.52	2.44E-02	1.88E-01	MCODE_5
GOTERM_CC_DIRECT	GO:0050768	negative regulation of neurogenesis	2	9.52	2.44E-02	1.88E-01	MCODE_5
GOTERM_CC_DIRECT	GO:0001782	B cell homeostasis	2	9.52	2.64E-02	1.98E-01	MCODE_5

GOTERM_CC_DIRECT	GO:0009314	response to radiation	2	9.52	2.64E-02	1.98E-01	MCODE_5
GOTERM_CC_DIRECT	GO:0045672	positive regulation of osteoclast differentiation	2	9.52	2.74E-02	2.03E-01	MCODE_5
GOTERM_CC_DIRECT	GO:0043200	response to amino acid	2	9.52	2.84E-02	2.05E-01	MCODE_5
GOTERM_CC_DIRECT	GO:0051928	positive regulation of calcium ion transport	2	9.52	2.84E-02	2.05E-01	MCODE_5
GOTERM_CC_DIRECT	GO:0045840	positive regulation of mitotic nuclear division	2	9.52	3.04E-02	2.17E-01	MCODE_5
GOTERM_CC_DIRECT	GO:0048873	homeostasis of number of cells within a tissue	2	9.52	3.14E-02	2.22E-01	MCODE_5
GOTERM_CC_DIRECT	GO:0009410	response to xenobiotic stimulus	3	14.29	3.24E-02	2.26E-01	MCODE_5
GOTERM_CC_DIRECT	GO:2001234	negative regulation of apoptotic signaling pathway	2	9.52	3.44E-02	2.34E-01	MCODE_5
GOTERM_CC_DIRECT	GO:0070269	pyroptosis	2	9.52	3.44E-02	2.34E-01	MCODE_5
GOTERM_CC_DIRECT	GO:0097192	extrinsic apoptotic signaling pathway in absence of ligand	2	9.52	3.54E-02	2.38E-01	MCODE_5
GOTERM_CC_DIRECT	GO:0050832	defense response to fungus	2	9.52	3.64E-02	2.38E-01	MCODE_5
GOTERM_CC_DIRECT	GO:2001235	positive regulation of apoptotic signaling pathway	2	9.52	3.64E-02	2.38E-01	MCODE_5
GOTERM_CC_DIRECT	GO:0006974	cellular response to DNA damage stimulus	3	14.29	3.70E-02	2.38E-01	MCODE_5
GOTERM_CC_DIRECT	GO:0043122	regulation of I-kappaB kinase/NF-kappaB signaling	2	9.52	3.74E-02	2.38E-01	MCODE_5
GOTERM_CC_DIRECT	GO:0050778	positive regulation of immune response	2	9.52	3.74E-02	2.38E-01	MCODE_5
GOTERM_CC_DIRECT	GO:0043029	T cell homeostasis	2	9.52	3.84E-02	2.42E-01	MCODE_5
GOTERM_CC_DIRECT	GO:0032717	negative regulation of interleukin-8 production	2	9.52	3.93E-02	2.46E-01	MCODE_5
GOTERM_CC_DIRECT	GO:0008625	extrinsic apoptotic signaling pathway via death domain receptors	2	9.52	4.03E-02	2.49E-01	MCODE_5

GOTERM_CC_DIRECT	GO:0035094	response to nicotine	2	9.52	4.13E-02	2.51E-01	MCODE_5
GOTERM_CC_DIRECT	GO:0007165	signal transduction	5	23.81	4.16E-02	2.51E-01	MCODE_5
GOTERM_CC_DIRECT	GO:0010629	negative regulation of gene expression	3	14.29	4.19E-02	2.51E-01	MCODE_5
GOTERM_CC_DIRECT	GO:0043507	positive regulation of JUN kinase activity	2	9.52	4.33E-02	2.57E-01	MCODE_5
GOTERM_CC_DIRECT	GO:0045071	negative regulation of viral genome replication	2	9.52	4.53E-02	2.61E-01	MCODE_5
GOTERM_CC_DIRECT	GO:0042542	response to hydrogen peroxide	2	9.52	4.53E-02	2.61E-01	MCODE_5
GOTERM_CC_DIRECT	GO:0019882	antigen processing and presentation	2	9.52	4.53E-02	2.61E-01	MCODE_5
GOTERM_CC_DIRECT	GO:0070498	interleukin-1-mediated signaling pathway	2	9.52	4.62E-02	2.62E-01	MCODE_5
GOTERM_CC_DIRECT	GO:0050796	regulation of insulin secretion	2	9.52	4.62E-02	2.62E-01	MCODE_5
GOTERM_CC_DIRECT	GO:0045429	positive regulation of nitric oxide biosynthetic process	2	9.52	4.72E-02	2.65E-01	MCODE_5
GOTERM_CC_DIRECT	GO:0038095	Fc-epsilon receptor signaling pathway	2	9.52	4.82E-02	2.68E-01	MCODE_5
GOTERM_CC_DIRECT	GO:0071407	cellular response to organic cyclic compound	2	9.52	4.92E-02	2.71E-01	MCODE_5
GOTERM_CC_DIRECT	GO:0050766	positive regulation of phagocytosis	2	9.52	5.02E-02	2.74E-01	MCODE_5
GOTERM_CC_DIRECT	GO:0043280	positive regulation of cysteine-type endopeptidase activity involved in apoptotic process	2	9.52	5.21E-02	2.79E-01	MCODE_5
GOTERM_CC_DIRECT	GO:0008630	intrinsic apoptotic signaling pathway in response to DNA damage	2	9.52	5.21E-02	2.79E-01	MCODE_5
GOTERM_CC_DIRECT	GO:1904707	positive regulation of vascular smooth muscle cell proliferation	2	9.52	5.51E-02	2.92E-01	MCODE_5
GOTERM_CC_DIRECT	GO:0043154	negative regulation of cysteine-type endopeptidase activity involved in apoptotic process	2	9.52	5.60E-02	2.95E-01	MCODE_5

GOTERM_CC_DIRECT	GO:0016241	regulation of macroautophagy	2	9.52	5.70E-02	2.97E-01	MCODE_5
GOTERM_CC_DIRECT	GO:0048661	positive regulation of smooth muscle cell proliferation	2	9.52	5.89E-02	3.02E-01	MCODE_5
GOTERM_CC_DIRECT	GO:0009612	response to mechanical stimulus	2	9.52	5.89E-02	3.02E-01	MCODE_5
GOTERM_CC_DIRECT	GO:0043525	positive regulation of neuron apoptotic process	2	9.52	6.19E-02	3.14E-01	MCODE_5
GOTERM_CC_DIRECT	GO:0001819	positive regulation of cytokine production	2	9.52	6.96E-02	3.51E-01	MCODE_5
GOTERM_CC_DIRECT	GO:0032715	negative regulation of interleukin-6 production	2	9.52	7.05E-02	3.52E-01	MCODE_5
GOTERM_CC_DIRECT	GO:1901224	positive regulation of NIK/NF-kappaB signaling	2	9.52	7.15E-02	3.52E-01	MCODE_5
GOTERM_CC_DIRECT	GO:0018107	peptidyl-threonine phosphorylation	2	9.52	7.15E-02	3.52E-01	MCODE_5
GOTERM_CC_DIRECT	GO:0030163	protein catabolic process	2	9.52	7.44E-02	3.63E-01	MCODE_5
GOTERM_CC_DIRECT	GO:0071260	cellular response to mechanical stimulus	2	9.52	7.72E-02	3.74E-01	MCODE_5
GOTERM_CC_DIRECT	GO:0043491	protein kinase B signaling	2	9.52	8.01E-02	3.81E-01	MCODE_5
GOTERM_CC_DIRECT	GO:0009636	response to toxic substance	2	9.52	8.01E-02	3.81E-01	MCODE_5
GOTERM_CC_DIRECT	GO:0001503	ossification	2	9.52	8.67E-02	4.10E-01	MCODE_5
GOTERM_CC_DIRECT	GO:0006816	calcium ion transport	2	9.52	8.95E-02	4.20E-01	MCODE_5
GOTERM_CC_DIRECT	GO:0033138	positive regulation of peptidyl-serine phosphorylation	2	9.52	9.14E-02	4.25E-01	MCODE_5
GOTERM_CC_DIRECT	GO:0031647	regulation of protein stability	2	9.52	9.33E-02	4.27E-01	MCODE_5
GOTERM_CC_DIRECT	GO:0032880	regulation of protein localization	2	9.52	9.33E-02	4.27E-01	MCODE_5
GOTERM_CC_DIRECT	GO:0071346	cellular response to interferon-gamma	2	9.52	9.79E-02	4.44E-01	MCODE_5

GOTERM_CC_DIRECT	GO:0010628	positive regulation of gene expression	3	14.29	9.84E-02	4.44E-01	MCODE_5
GOTERM_MF_DIRECT	GO:0005886	plasma membrane	13	92.86	1.13E-06	8.79E-05	MCODE_1
GOTERM_MF_DIRECT	GO:0031234	extrinsic component of cytoplasmic side of plasma membrane	4	28.57	1.23E-05	4.79E-04	MCODE_1
GOTERM_MF_DIRECT	GO:0005737	cytoplasm	12	85.71	3.63E-05	9.43E-04	MCODE_1
GOTERM_MF_DIRECT	GO:0005829	cytosol	10	71.43	1.93E-03	3.51E-02	MCODE_1
GOTERM_MF_DIRECT	GO:0005925	focal adhesion	4	28.57	2.25E-03	3.51E-02	MCODE_1
GOTERM_MF_DIRECT	GO:0005634	nucleus	10	71.43	3.59E-03	4.04E-02	MCODE_1
GOTERM_MF_DIRECT	GO:0016020	membrane	8	57.14	3.63E-03	4.04E-02	MCODE_1
GOTERM_MF_DIRECT	GO:0005943	phosphatidylinositol 3-kinase complex, class IA	2	14.29	5.65E-03	5.51E-02	MCODE_1
GOTERM_MF_DIRECT	GO:0043235	receptor complex	3	21.43	8.51E-03	6.32E-02	MCODE_1
GOTERM_MF_DIRECT	GO:0032991	macromolecular complex	4	28.57	8.66E-03	6.32E-02	MCODE_1
GOTERM_MF_DIRECT	GO:0030054	cell junction	3	21.43	9.48E-03	6.32E-02	MCODE_1
GOTERM_MF_DIRECT	GO:0045121	membrane raft	3	21.43	1.03E-02	6.32E-02	MCODE_1
GOTERM_MF_DIRECT	GO:0048471	perinuclear region of cytoplasm	4	28.57	1.05E-02	6.32E-02	MCODE_1
GOTERM_MF_DIRECT	GO:0005942	phosphatidylinositol 3-kinase complex	2	14.29	1.38E-02	7.67E-02	MCODE_1
GOTERM_MF_DIRECT	GO:0005768	endosome	3	21.43	1.71E-02	8.87E-02	MCODE_1
GOTERM_MF_DIRECT	GO:0019897	extrinsic component of plasma membrane	2	14.29	2.24E-02	1.09E-01	MCODE_1
GOTERM_MF_DIRECT	GO:0000791	euchromatin	2	14.29	3.83E-02	1.76E-01	MCODE_1

GOTERM_MF_DIRECT	GO:0005856	cytoskeleton	3	21.43	4.66E-02	2.02E-01	MCODE_1
GOTERM_MF_DIRECT	GO:0032587	ruffle membrane	2	14.29	6.17E-02	2.53E-01	MCODE_1
GOTERM_MF_DIRECT	GO:0005654	nucleoplasm	6	42.86	9.45E-02	3.69E-01	MCODE_1
GOTERM_MF_DIRECT	GO:0005789	endoplasmic reticulum membrane	4	66.67	1.52E-03	2.52E-02	MCODE_2
GOTERM_MF_DIRECT	GO:0005737	cytoplasm	6	100.00	1.74E-03	2.52E-02	MCODE_2
GOTERM_MF_DIRECT	GO:0043231	intracellular membrane-bounded organelle	3	50.00	2.18E-02	2.11E-01	MCODE_2
GOTERM_MF_DIRECT	GO:0043005	neuron projection	2	33.33	8.75E-02	6.35E-01	MCODE_2
GOTERM_MF_DIRECT	GO:0005829	cytosol	6	100.00	1.50E-03	1.80E-02	MCODE_3
GOTERM_MF_DIRECT	GO:0070062	extracellular exosome	4	66.67	1.08E-02	6.45E-02	MCODE_3
GOTERM_MF_DIRECT	GO:0034774	secretory granule lumen	2	33.33	2.78E-02	1.11E-01	MCODE_3
GOTERM_MF_DIRECT	GO:0005739	mitochondrion	4	80.00	1.35E-03	2.35E-02	MCODE_4
GOTERM_MF_DIRECT	GO:0005654	nucleoplasm	5	100.00	1.51E-03	2.35E-02	MCODE_4
GOTERM_MF_DIRECT	GO:0005634	nucleus	5	100.00	7.62E-03	7.88E-02	MCODE_4
GOTERM_MF_DIRECT	GO:1904813	ficolin-1-rich granule lumen	2	40.00	2.40E-02	1.86E-01	MCODE_4
GOTERM_MF_DIRECT	GO:0005829	cytosol	4	80.00	6.44E-02	3.99E-01	MCODE_4
GOTERM_MF_DIRECT	GO:0005764	lysosome	7	33.33	3.88E-07	1.28E-05	MCODE_5
GOTERM_MF_DIRECT	GO:0005615	extracellular space	12	57.14	4.73E-07	1.28E-05	MCODE_5
GOTERM_MF_DIRECT	GO:0005576	extracellular region	12	57.14	1.39E-06	2.50E-05	MCODE_5
GOTERM_MF_DIRECT	GO:0030141	secretory granule	5	23.81	2.09E-06	2.82E-05	MCODE_5

GOTERM_MF_DIRECT	GO:0036021	endolysosome lumen	3	14.29	8.89E-06	9.60E-05	MCODE_5
GOTERM_MF_DIRECT	GO:0035580	specific granule lumen	4	19.05	2.97E-05	2.67E-04	MCODE_5
GOTERM_MF_DIRECT	GO:0009897	external side of plasma membrane	6	28.57	8.01E-05	6.03E-04	MCODE_5
GOTERM_MF_DIRECT	GO:0035578	azurophil granule lumen	4	19.05	8.93E-05	6.03E-04	MCODE_5
GOTERM_MF_DIRECT	GO:0009986	cell surface	6	28.57	3.36E-04	2.02E-03	MCODE_5
GOTERM_MF_DIRECT	GO:1904724	tertiary granule lumen	3	14.29	1.33E-03	7.18E-03	MCODE_5
GOTERM_MF_DIRECT	GO:0070062	extracellular exosome	8	38.10	3.77E-03	1.80E-02	MCODE_5
GOTERM_MF_DIRECT	GO:0043202	lysosomal lumen	3	14.29	4.00E-03	1.80E-02	MCODE_5
GOTERM_MF_DIRECT	GO:0097013	phagocytic vesicle lumen	2	9.52	4.83E-03	2.01E-02	MCODE_5
GOTERM_MF_DIRECT	GO:1904813	ficolin-1-rich granule lumen	3	14.29	6.43E-03	2.48E-02	MCODE_5
GOTERM_MF_DIRECT	GO:0005737	cytoplasm	12	57.14	1.01E-02	3.64E-02	MCODE_5
GOTERM_MF_DIRECT	GO:0043231	intracellular membrane-bounded organelle	5	23.81	1.49E-02	5.04E-02	MCODE_5
GOTERM_MF_DIRECT	GO:0045121	membrane raft	3	14.29	2.38E-02	7.57E-02	MCODE_5
GOTERM_MF_DIRECT	GO:0005887	integral component of plasma membrane	5	23.81	4.79E-02	1.44E-01	MCODE_5
GOTERM_MF_DIRECT	GO:0043025	neuronal cell body	3	14.29	5.23E-02	1.49E-01	MCODE_5
GOTERM_MF_DIRECT	GO:0005886	plasma membrane	10	47.62	5.64E-02	1.52E-01	MCODE_5
GOTERM_MF_DIRECT	GO:0045335	phagocytic vesicle	2	9.52	6.75E-02	1.73E-01	MCODE_5
GOTERM_MF_DIRECT	GO:0010494	cytoplasmic stress granule	2	9.52	8.81E-02	2.16E-01	MCODE_5

GOTERM_MF_DIRECT	GO:0042470	melanosome	2	9.52	9.52E-02	2.23E-01	MCODE_5
GOTERM_MF_DIRECT	GO:0004713	Protein tyrosine kinase activity	7	50.00	6.83E-11	4.44E-09	MCODE_1
GOTERM_MF_DIRECT	GO:0004712	Protein serine/threonine/tyrosine kinase activity	9	64.29	9.98E-11	4.44E-09	MCODE_1
GOTERM_MF_DIRECT	GO:0005524	ATP binding	10	71.43	7.95E-08	2.36E-06	MCODE_1
GOTERM_MF_DIRECT	GO:0004715	Non-membrane spanning protein tyrosine kinase activity	4	28.57	3.75E-06	6.97E-05	MCODE_1
GOTERM_MF_DIRECT	GO:0019899	Enzyme binding	6	42.86	3.92E-06	6.97E-05	MCODE_1
GOTERM_MF_DIRECT	GO:0030235	Nitric-oxide synthase regulator activity	3	21.43	1.21E-05	1.80E-04	MCODE_1
GOTERM_MF_DIRECT	GO:0019901	Protein kinase binding	6	42.86	1.67E-05	2.13E-04	MCODE_1
GOTERM_MF_DIRECT	GO:0004672	Protein kinase activity	5	35.71	1.02E-04	1.14E-03	MCODE_1
GOTERM_MF_DIRECT	GO:0005178	Integrin binding	4	28.57	1.58E-04	1.56E-03	MCODE_1
GOTERM_MF_DIRECT	GO:0016301	Kinase activity	4	28.57	5.33E-04	4.74E-03	MCODE_1
GOTERM_MF_DIRECT	GO:0004714	Transmembrane receptor protein tyrosine kinase activity	3	21.43	6.08E-04	4.92E-03	MCODE_1
GOTERM_MF_DIRECT	GO:0045296	Cadherin binding	4	28.57	1.20E-03	8.89E-03	MCODE_1
GOTERM_MF_DIRECT	GO:0051117	ATPase binding	3	21.43	1.53E-03	1.05E-02	MCODE_1
GOTERM_MF_DIRECT	GO:0019903	Protein phosphatase binding	3	21.43	1.71E-03	1.09E-02	MCODE_1
GOTERM_MF_DIRECT	GO:0004725	Protein tyrosine phosphatase activity	3	21.43	2.45E-03	1.45E-02	MCODE_1
GOTERM_MF_DIRECT	GO:0038085	Vascular endothelial growth factor binding	2	14.29	3.42E-03	1.79E-02	MCODE_1
GOTERM_MF_DIRECT	GO:0035004	Phosphatidylinositol 3-kinase activity	2	14.29	3.42E-03	1.79E-02	MCODE_1

GOTERM_MF_DIRECT	GO:0035005	1-phosphatidylinositol-4-phosphate 3-kinase activity	2	14.29	4.79E-03	2.24E-02	MCODE_1
GOTERM_MF_DIRECT	GO:0046934	Phosphatidylinositol-4,5-bisphosphate 3-kinase activity	2	14.29	4.79E-03	2.24E-02	MCODE_1
GOTERM_MF_DIRECT	GO:0005515	Protein binding	14	100.00	5.16E-03	2.30E-02	MCODE_1
GOTERM_MF_DIRECT	GO:0052812	Phosphatidylinositol-3,4-bisphosphate 5-kinase activity	2	14.29	6.15E-03	2.61E-02	MCODE_1
GOTERM_MF_DIRECT	GO:0016303	1-phosphatidylinositol-3-kinase activity	2	14.29	6.83E-03	2.76E-02	MCODE_1
GOTERM_MF_DIRECT	GO:0005131	Growth hormone receptor binding	2	14.29	7.51E-03	2.91E-02	MCODE_1
GOTERM_MF_DIRECT	GO:0004726	Non-membrane spanning protein tyrosine phosphatase activity	2	14.29	8.87E-03	3.29E-02	MCODE_1
GOTERM_MF_DIRECT	GO:0052742	Phosphatidylinositol kinase activity	2	14.29	1.09E-02	3.88E-02	MCODE_1
GOTERM_MF_DIRECT	GO:0005158	Insulin receptor binding	2	14.29	1.56E-02	5.36E-02	MCODE_1
GOTERM_MF_DIRECT	GO:0071889	14-3-3 protein binding	2	14.29	2.31E-02	7.60E-02	MCODE_1
GOTERM_MF_DIRECT	GO:0042802	Identical protein binding	5	35.71	2.55E-02	8.11E-02	MCODE_1
GOTERM_MF_DIRECT	GO:0019838	Growth factor binding	2	14.29	2.71E-02	8.15E-02	MCODE_1
GOTERM_MF_DIRECT	GO:0042169	SH2 domain binding	2	14.29	2.77E-02	8.15E-02	MCODE_1
GOTERM_MF_DIRECT	GO:0030331	Estrogen receptor binding	2	14.29	2.84E-02	8.15E-02	MCODE_1
GOTERM_MF_DIRECT	GO:0005102	Receptor binding	3	21.43	3.13E-02	8.71E-02	MCODE_1
GOTERM_MF_DIRECT	GO:0030971	Receptor tyrosine kinase binding	2	14.29	4.43E-02	1.18E-01	MCODE_1
GOTERM_MF_DIRECT	GO:0003682	Chromatin binding	3	21.43	4.49E-02	1.18E-01	MCODE_1
GOTERM_MF_DIRECT	GO:0019900	Kinase binding	2	14.29	7.03E-02	1.79E-01	MCODE_1

GOTERM_MF_DIRECT	GO:0003690	Double-stranded DNA binding	2	14.29	7.79E-02	1.93E-01	MCODE_1
GOTERM_MF_DIRECT	GO:0016702	Oxidoreductase activity, acting on single donors with incorporation of molecular oxygen, incorporation of two atoms of oxygen	4	66.67	3.19E-09	9.90E-08	MCODE_2
GOTERM_MF_DIRECT	GO:0005506	Iron ion binding	4	66.67	4.32E-06	5.46E-05	MCODE_2
GOTERM_MF_DIRECT	GO:0020037	Heme binding	4	66.67	5.28E-06	5.46E-05	MCODE_2
GOTERM_MF_DIRECT	GO:0018676	(S)-limonene 7-monooxygenase activity	2	33.33	5.27E-04	2.33E-03	MCODE_2
GOTERM_MF_DIRECT	GO:0004666	Prostaglandin-endoperoxide synthase activity	2	33.33	5.27E-04	2.33E-03	MCODE_2
GOTERM_MF_DIRECT	GO:0018675	(S)-limonene 6-monooxygenase activity	2	33.33	5.27E-04	2.33E-03	MCODE_2
GOTERM_MF_DIRECT	GO:0052741	(R)-limonene 6-monooxygenase activity	2	33.33	5.27E-04	2.33E-03	MCODE_2
GOTERM_MF_DIRECT	GO:0004052	Arachidonate 12-lipoxygenase activity	2	33.33	1.05E-03	4.08E-03	MCODE_2
GOTERM_MF_DIRECT	GO:0008392	Arachidonic acid epoxygenase activity	2	33.33	5.00E-03	1.72E-02	MCODE_2
GOTERM_MF_DIRECT	GO:0016712	Oxidoreductase activity, acting on paired donors, with incorporation or reduction of molecular oxygen, reduced flavin or flavoprotein as one donor, and incorporation of one atom of oxygen	2	33.33	6.31E-03	1.96E-02	MCODE_2
GOTERM_MF_DIRECT	GO:0070330	Aromatase activity	2	33.33	8.67E-03	2.31E-02	MCODE_2
GOTERM_MF_DIRECT	GO:0008395	Steroid hydroxylase activity	2	33.33	8.93E-03	2.31E-02	MCODE_2
GOTERM_MF_DIRECT	GO:0004601	Peroxidase activity	2	33.33	9.71E-03	2.32E-02	MCODE_2

GOTERM_MF_DIRECT	GO:0016705	Oxidoreductase activity, acting on paired donors, with incorporation or reduction of molecular oxygen	2	33.33	1.60E-02	3.54E-02	MCODE_2
GOTERM_MF_DIRECT	GO:0004497	Monooxygenase activity	2	33.33	2.06E-02	4.27E-02	MCODE_2
GOTERM_MF_DIRECT	GO:0016491	Oxidoreductase activity	2	33.33	5.99E-02	1.16E-01	MCODE_2
GOTERM_MF_DIRECT	GO:0019899	Enzyme binding	2	33.33	9.81E-02	1.79E-01	MCODE_2
GOTERM_MF_DIRECT	GO:0046872	Metal ion binding	4	66.67	2.37E-02	2.50E-01	MCODE_3
GOTERM_MF_DIRECT	GO:0000166	Nucleotide binding	2	33.33	2.50E-02	2.50E-01	MCODE_3
GOTERM_MF_DIRECT	GO:0042802	Identical protein binding	3	50.00	6.95E-02	4.63E-01	MCODE_3
GOTERM_MF_DIRECT	GO:0004707	MAP kinase activity	3	60.00	4.00E-06	1.40E-04	MCODE_4
GOTERM_MF_DIRECT	GO:0004674	Protein serine/threonine kinase activity	3	60.00	2.62E-03	3.50E-02	MCODE_4
GOTERM_MF_DIRECT	GO:0004712	Protein serine/threonine/tyrosine kinase activity	3	60.00	3.19E-03	3.50E-02	MCODE_4
GOTERM_MF_DIRECT	GO:0004708	MAP kinase kinase activity	2	40.00	4.00E-03	3.50E-02	MCODE_4
GOTERM_MF_DIRECT	GO:0005524	ATP binding	3	60.00	3.54E-02	2.48E-01	MCODE_4
GOTERM_MF_DIRECT	GO:0043565	Sequence-specific DNA binding	2	40.00	7.10E-02	3.47E-01	MCODE_4
GOTERM_MF_DIRECT	GO:0004672	Protein kinase activity	2	40.00	7.85E-02	3.47E-01	MCODE_4
GOTERM_MF_DIRECT	GO:0019899	Enzyme binding	2	40.00	7.93E-02	3.47E-01	MCODE_4
GOTERM_MF_DIRECT	GO:0016493	C-C chemokine receptor activity	4	19.05	1.75E-06	9.89E-05	MCODE_5
GOTERM_MF_DIRECT	GO:0019957	C-C chemokine binding	4	19.05	2.00E-06	9.89E-05	MCODE_5
GOTERM_MF_DIRECT	GO:0004197	Cysteine-type endopeptidase activity	5	23.81	3.17E-06	9.91E-05	MCODE_5

GOTERM_MF_DIRECT	GO:0008233	Peptidase activity	5	23.81	4.01E-06	9.91E-05	MCODE_5
GOTERM_MF_DIRECT	GO:0043394	Proteoglycan binding	3	14.29	9.53E-05	1.89E-03	MCODE_5
GOTERM_MF_DIRECT	GO:0004950	Chemokine receptor activity	3	14.29	1.79E-04	2.95E-03	MCODE_5
GOTERM_MF_DIRECT	GO:0002020	Protease binding	4	19.05	2.18E-04	3.08E-03	MCODE_5
GOTERM_MF_DIRECT	GO:0008234	Cysteine-type peptidase activity	3	14.29	7.25E-04	8.97E-03	MCODE_5
GOTERM_MF_DIRECT	GO:0008201	Heparin binding	4	19.05	8.36E-04	9.20E-03	MCODE_5
GOTERM_MF_DIRECT	GO:0004252	Serine-type endopeptidase activity	4	19.05	9.49E-04	9.39E-03	MCODE_5
GOTERM_MF_DIRECT	GO:0035717	Chemokine (C-C motif) ligand 7 binding	2	9.52	2.11E-03	1.74E-02	MCODE_5
GOTERM_MF_DIRECT	GO:0071791	Chemokine (C-C motif) ligand 5 binding	2	9.52	2.11E-03	1.74E-02	MCODE_5
GOTERM_MF_DIRECT	GO:0005518	Collagen binding	3	14.29	2.31E-03	1.76E-02	MCODE_5
GOTERM_MF_DIRECT	GO:0042802	Identical protein binding	7	33.33	7.34E-03	5.19E-02	MCODE_5
GOTERM_MF_DIRECT	GO:0004707	MAP kinase activity	2	9.52	1.67E-02	1.03E-01	MCODE_5
GOTERM_MF_DIRECT	GO:0005125	Cytokine activity	3	14.29	1.72E-02	1.03E-01	MCODE_5
GOTERM_MF_DIRECT	GO:0005515	Protein binding	19	90.48	1.77E-02	1.03E-01	MCODE_5
GOTERM_MF_DIRECT	GO:0008656	Cysteine-type endopeptidase activator activity involved in apoptotic process	2	9.52	2.50E-02	1.38E-01	MCODE_5
GOTERM_MF_DIRECT	GO:0004435	Phosphatidylinositol phospholipase C activity	2	9.52	2.71E-02	1.41E-01	MCODE_5
GOTERM_MF_DIRECT	GO:0001968	Fibronectin binding	2	9.52	3.32E-02	1.64E-01	MCODE_5
GOTERM_MF_DIRECT	GO:0004869	Cysteine-type endopeptidase inhibitor activity	2	9.52	3.83E-02	1.81E-01	MCODE_5

GOTERM_MF_DIRECT	GO:0001530	Lipopolysaccharide binding	2	9.52	4.13E-02	1.82E-01	MCODE_5
GOTERM_MF_DIRECT	GO:0004190	Aspartic-type endopeptidase activity	2	9.52	4.24E-02	1.82E-01	MCODE_5

Table S10 Response stability in QC sample

NO.	Ion Mode	Retention Time（min）	*m/z*	Area RSD（%）
IS1	Positive Mode	0.49	127.0801	1.83
IS2	Positive Mode	2.73	127.1425	1.76
IS3	Positive Mode	3.64	110.108	1.09
IS4	Negative Mode	2.21	92.0345	3.30
IS5	Negative Mode	2.66	133.1061	0.68
IS6	Negative Mode	3.77	121.0447	0.95

Table S11 Summary of PLS-DA model parameters for evaluating model quality by 200 permutation tests of corresponding validation plots

Group	A	N	R^2X（cum）	R^2Y（cum）	Q^2（cum）
NC vs CIA	1+1+0	16	0.244	0.977	0.591
CIA vs CM	1+1+0	16	0.293	0.991	0.753

Table S12 Identification of potential differential metabolites in different group

NO	Metabolites	Retention time(min)	*m*/*z*	CIA vs NC			CIA vs CM		
				VIP	FC	Change trend	VIP	FC	Change trend
1	L-Tryptophan	2.63	205.0970	1.41	-0.68	↑*	1.77	1.69	↓**
2	Phosphorylcholine	1.88	184.0730	1.74	-0.8	↑*	1.88	1.28	↓**
3	SM(d16:1/24:1(15Z))	1.78	785.6495	1.74	-0.79	↑*	1.5	1.47	↓**
4	5,6-Dihydrouridine	1.38	247.0917	1.46	-0.82	↑*	2.1	1.53	↓**
5	5-Aminopentanal	2.53	102.0913	1.64	-0.56	↑*	1.8	1.82	↓**
6	L-Asparagine	3.69	133.0605	1.58	-0.72	↑*	2.02	1.98	↓**
7	L-Allothreonine	4.17	120.0653	1.74	-0.68	↑*	1.86	1.8	↓**
8	4-Acetamidobutanoic acid	2.56	146.0809	1.55	-0.65	↑*	2.07	2.16	↓**
9	1-Pyrroline-5-carboxylic acid	3.19	114.0549	1.2	-0.72	↑*	1.52	1.4	↓**
10	Glyceric acid	3.04	105.0195	1.87	-0.69	↑*	1.41	1.37	↓*
11	2,6-Dimethylaniline	0.27	122.0963	1.51	-0.72	↑*	2.35	3.21	↓**
12	PC(22:1(13Z)/14:1(9Z))	0.34	786.5955	1.43	-0.88	↑*	1.45	1.2	↓*
13	N,N-Dimethylformamide	3.46	74.0600	1.67	-0.64	↑*	1.9	2.34	↓*
14	cis-4-Hydroxyequol	1.47	259.0918	1.59	-0.64	↑*	2.19	3.43	↓**
15	N-a-Acetylcitrulline	3.84	218.1137	1.47	-0.65	↑*	1.67	1.85	↓**
16	SM(d18:1/18:1(9Z))	1.82	729.5903	1.94	-0.74	↑**	1.86	2.48	↓*
17	3-Hydroxyisovalerylcarnitine	3.07	262.1643	1.55	-0.76	↑*	1.97	1.6	↓**
18	Glaucarubin	2.16	479.2293	1.81	-0.61	↑**	1.62	2.15	↓*
19	LysoPC(16:0)	5.64	496.3385	2.11	-0.48	↑*	1.86	1.89	↓**
20	2-(4-Methyl-5-thiazolyl)ethyl decanoate	2.69	298.1858	1.69	-0.65	↑*	1.36	1.22	↓*
